# Supplementary material for: Smartphone-delivered self-management for first-episode psychosis: the ARIES feasibility randomised controlled trial
Source: BMJ Open. 2020 Aug 26;10(8):e034927. doi: 10.1136/bmjopen-2019-034927 (PMC7451533; doi:10.1136/bmjopen-2019-034927)
Supplement: Supplementary data [file bmjopen-2019-034927supp002.pdf]

Service user participant consent form

Study Title: App to support Recovery In Early intervention Services (the ARIES study): Pilot randomised controlled trial of a self-management smartphone application

Principal Investigators: Professor Sonia Johnson and Professor David Osborn

Please Initial Each Box

1. I confirm that I have read and understood the Participant Information Sheet V5 dated 29/05/2017 for the above study and have had the opportunity to ask questions about the study. ☐
2. I understand that my participation is voluntary and that I am free to withhold personal information or to withdraw my participation at any time, without giving any reason, and without my medical care or legal rights being affected. ☐
3. I understand that if I choose to withdraw from the study that any data that I have already provided for the purposes of the research will be kept and used by the research team. ☐
4. I give permission for my General Practitioner (GP) and my Early Intervention team to be told I am participating in this study. ☐
5. I understand that relevant sections of my medical notes and data collected during the study may be looked at by regulatory authorities or from the NHS Trust, where it is relevant to my taking part in this research. I give permission for these individuals to have access to my records. ☐
6. I understand that I will be given a £20 gift as cash for my participation in each study assessment. ☐
7. I agree to the research team consulting NHS electronic records to investigate my diagnosis, medication, and mental health service use, and give them permission to do so even if I choose to no longer participate in the intervention, or they are not able to carry out further study interviews with me. ☐
8. I understand that in the event that I disclose information which may indicate new risk to myself or others, the researcher will be obliged to follow NHS Trust risk procedures that may require release of my personal data. ☐
9. I give permission for findings from the study to be written up for publication. Any publication will not identify me. ☐
10. I give permission to be audio recorded where required for the purposes of the study. I understand these audio-recordings will be transcribed and anonymised and audio recordings destroyed after the study. I give permission for direct quotations taken from this interview to be included in papers written for publication. Any quotation would not identify me. ☐
11. I give permission for the research team to collect data from the My Journey 3 app regarding the frequency, duration, and pattern of my use of it. I understand that no personal information will be collected from the app. ☐
12. I give permission for non-identifiable data to be shared with other research teams for research purposes. ☐

Steare T, *et al. BMJ Open* 2020; 10:e034927. doi: 10.1136/bmjopen-2019-034927

13. I agree to take part in this study. ☐

_______________________ _______________ _______________________
Name of participant Date Signature

_______________________ _______________ _______________________
Name of Researcher taking consent Date Signature
