# Supplementary material for: Smartphone-delivered self-management for first-episode psychosis: the ARIES feasibility randomised controlled trial
Source: BMJ Open. 2020 Aug 26;10(8):e034927. doi: 10.1136/bmjopen-2019-034927 (PMC7451533; doi:10.1136/bmjopen-2019-034927)
Supplement: Author's manuscript [file bmjopen-2019-034927.draft_revisions.pdf]

BMJ Open is committed to open peer review. As part of this commitment we make the peer review history of every article we publish publicly available.

When an article is published we post the peer reviewers' comments and the authors' responses online. We also post the versions of the paper that were used during peer review. These are the versions that the peer review comments apply to.

The versions of the paper that follow are the versions that were submitted during the peer review process. They are not the versions of record or the final published versions. They should not be cited or distributed as the published version of this manuscript.

BMJ Open is an open access journal and the full, final, typeset and author-corrected version of record of the manuscript is available on our site with no access controls, subscription charges or pay-per-view fees (http://bmjopen.bmj.com).

If you have any questions on BMJ Open's open peer review process please email info.bmjopen@bmj.com

Smartphone-delivered self-management for first-episode psychosis: the ARIES feasibility randomised controlled trial

Journal:	*BMJ Open*
Manuscript ID	bmjopen-2019-034927
Article Type:	Original research
Date Submitted by the Author:	11-Oct-2019

Complete List of Authors:	Steare, Thomas; University College London, Division of Psychiatry<br>O'Hanlon, Puffin; University College London, Division of Psychiatry<br>Eskinazi, Michelle; University College London, Division of Psychiatry<br>Osborn, David; University College London, Division of Psychiatry; Camden and Islington NHS Foundation Trust<br>Lloyd-Evans, Brynmor ; University College London, Division of Psychiatry; Camden and Islington NHS Foundation Trust<br>Jones, Rebecca; University College London, Division of Psychiatry<br>Rostill, Helen; University of Surrey; Surrey and Borders Partnership NHS Foundation Trust<br>Amani, Sarah; Early Intervention in Psychosis Programme (South of England)<br>Johnson, Sonia; University College London, Division of Psychiatry; Camden and Islington NHS Foundation Trust

Keywords:	Schizophrenia & psychotic disorders < PSYCHIATRY, MENTAL HEALTH, Clinical trials < THERAPEUTICS

SCHOLARONE™
Manuscripts

I, the Submitting Author has the right to grant and does grant on behalf of all authors of the Work (as defined in the below author licence), an exclusive licence and/or a non-exclusive licence for contributions from authors who are: i) UK Crown employees; ii) where BMJ has agreed a CC-BY licence shall apply, and/or iii) in accordance with the terms applicable for US Federal Government officers or employees acting as part of their official duties; on a worldwide, perpetual, irrevocable, royalty-free basis to BMJ Publishing Group Ltd ("BMJ") its licensees and where the relevant Journal is co-owned by BMJ to the co-owners of the Journal, to publish the Work in this journal and any other BMJ products and to exploit all rights, as set out in our licence.

The Submitting Author accepts and understands that any supply made under these terms is made by BMJ to the Submitting Author unless you are acting as an employee on behalf of your employer or a postgraduate student of an affiliated institution which is paying any applicable article publishing charge ("APC") for Open Access articles. Where the Submitting Author wishes to make the Work available on an Open Access basis (and intends to pay the relevant APC), the terms of reuse of such Open Access shall be governed by a Creative Commons licence – details of these licences and which Creative Commons licence will apply to this Work are set out in our licence referred to above.

Other than as permitted in any relevant BMJ Author's Self Archiving Policies, I confirm this Work has not been accepted for publication elsewhere, is not being considered for publication elsewhere and does not duplicate material already published. I confirm all authors consent to publication of this Work and authorise the granting of this licence.

For peer review only

TITLE PAGE

Title: Smartphone-delivered self-management for first-episode psychosis: the ARIES feasibility randomised controlled trial

Authors:

Thomas Steare

Division of Psychiatry, University College London, London, UK

Puffin O'Hanlon

Division of Psychiatry, University College London, London, UK

Michelle Eskinazi

Division of Psychiatry, University College London, London, UK

David Osborn

Division of Psychiatry, University College London, London, UK

R&D Department, Camden and Islington NHS Foundation Trust, London, UK

Brynmor Lloyd-Evans

Division of Psychiatry, University College London, London, UK

R&D Department, Camden and Islington NHS Foundation Trust, London, UK

Rebecca Jones

Division of Psychiatry, University College London, London, UK

Helen Rostill

University of Surrey, UK

Surrey and Borders Partnership NHS Foundation Trust, Leatherhead, Surrey, UK

Sarah Amani

EIP Programme (South of England), NHS England, Oxford, Oxfordshire, UK

For peer review only

Sonia Johnson (corresponding author)

Address: UCL Division of Psychiatry, 6th Floor, Wing B, Maple House, 149 Tottenham Court Road, London W1T 7NF

Division of Psychiatry, University College London, London, UK

R&D Department, Camden and Islington NHS Foundation Trust, London, UK

s.johnson@ucl.ac.uk

02076799453

Word count: 5,351

Keywords: Psychosis, Self-management, Smartphone, Feasibility study, Randomised controlled trial

For peer review only

ABSTRACT

Objectives: To test the feasibility and acceptability of a randomised controlled trial (RCT) to evaluate a Smartphone-based self-management tool in Early Intervention in Psychosis (EIP) services.

Design: A two-arm unblinded feasibility RCT.

Setting: Three NHS EIP services in England.

Participants: Adults using EIP services that own an Android Smartphone. Participants were recruited until the recruitment target was met (n=40).

Interventions: Participants were randomised with a 1:1 allocation to one of two conditions: (1) treatment as usual from EIP services (TAU) or (2) TAU plus access to My Journey 3 on their own Smartphone. My Journey 3 features a range of self-management components including access to digital recovery and relapse prevention plans, medication tracking and symptom monitoring. My Journey 3 use was at the users' discretion, and was supported by EIP service clinicians. Participants had access for a median of 38.1 weeks.

Primary and secondary outcome measures: Feasibility outcomes included recruitment, follow-up rates and intervention engagement. Participant data on mental health outcomes were collected from clinical records and from research assessments at baseline, 4 months and 12 months.

Results: 83% and 75% of participants were retained in the trial at the 4- and 12-month assessments. All treatment group participants had access to My Journey 3 during the trial, but technical difficulties caused delays in ensuring timely access to the intervention. The median

For peer review only

number of My Journey 3 uses was 16.5 (IQR 8.5 to 23) and median total minutes spent using My Journey 3 was 26.8 (IQR 18.3 to 57.3). No serious adverse events were reported.

Conclusions: Recruitment and retention were feasible. Within a trial context My Journey 3 could be successfully delivered to adults using EIP services, but with relatively low usage rates. Further evaluation of the intervention in a larger trial may be warranted, but should include attention to implementation.

Trial Registration: ISRCTN10004994

ARTICLE SUMMARY

Strengths and limitations of this study:

- We completed a feasibility trial to assess the feasibility of conducting a large scale trial and the acceptability of the intervention
- The intervention has been through substantial development including stakeholder input and refinement of the design and content based on feedback from lab and field testing
- We were not able to blind researchers or participants to their treatment allocation
- This is a feasibility study, and therefore we cannot draw conclusions regarding the effectiveness of the intervention.

For peer review only

INTRODUCTION

Early Intervention in Psychosis (EIP) services have been established across the United Kingdom to provide care to adults during the three years following an initial episode of psychosis. There is evidence that such services are effective and cost-effective,[1,2] resulting in improvement in a range of outcomes yet challenges remain. Relapse rates for EIP service users are high [3] particularly after discharge [4,5] and limited adherence with antipsychotic medication is common [6]. There are also difficulties accessing psychosocial interventions,[7] including supported self-management.

Illness self-management is an approach designed to support people to manage long-term health conditions by developing their ability to recognise and monitor symptoms and early warning signs of relapse, identify and avoid stressors, make plans for achieving their own recovery plans and to effectively use coping strategies.[8] For people with psychosis, self-management tools have been shown to reduce psychological distress, improve medication adherence and reduce the likelihood of future hospital admissions.[9-11] In a recent meta-analysis, self-management interventions for severe mental illness were also found to have a significant benefit on patient-valued outcomes of personal recovery, hope and self-efficacy.[12] Despite self-management programmes being mandated in current treatment guidelines for first-episode psychosis,[13] there is a lack of well-evaluated tools to support delivery within EIP services. There is a clear need to overcome implementation barriers affecting the delivery of self-management to those likely to benefit from it.[12] A potentially convenient and economical way of achieving this is via the use of digital technology such as Smartphones.[14]

Smartphones can run advanced software known as apps that hold promise as an effective tool to assist the monitoring and treatment of mental health problems. Smartphone ownership is

For peer review only

rapidly growing world-wide [15] with a significant number of developed countries with ownership rates of more than 80%.[16] Adults with severe mental health problems have comparable Smartphone ownership rates to the general population.[17-19] Smartphones also provide high accessibility to the internet and are commonly carried on the person, meaning apps can be easily accessed at times and locations convenient for the user. Accordingly Smartphones have the capacity to deliver time-unlimited mental health interventions, such as self-management, and ultimately the potential to increase access to effective care and reduce healthcare costs.[20] The benefits of Smartphone apps may also extend beyond the original treatment period with a community team, and could be a valuable tool following discharge where the risk of relapse is increased.[4,5]

The majority of digital health interventions that have been developed for psychosis have been based on existing psychological therapies such as Cognitive Behavioural Therapy,[21,22] or other evidence-based interventions,[23,24] yet very little is known regarding their effectiveness in EIP services. Adults with psychosis express favourable views about using Smartphones to access mental health interventions [25,26] and a number of apps have been found to be safe and acceptable.[27]

To date only one trial of a self-management app for EIP services has published its results.[28] In the proof-of-concept trial an active self-management app "Actissist" conferred benefits over a passive control app. The study suggests that participants that received Actissist had better outcomes regarding their mood and general and negative symptoms post-treatment in comparison to control participants. Actissist features a range of components including self-assessment questions focused on cognitive appraisals, emotions, behaviours and belief convictions and suggests appropriate coping strategies, but does not feature some major cornerstones of self-

For peer review only

management such as relapse and recovery plans. Regardless results from this study suggest that such digital self-management interventions could potentially improve outcomes of people using EIP services. Further trials are needed before firm conclusions can be made regarding the feasibility of conducting RCTs in this field and the therapeutic benefits of self-management apps for first-episode psychosis. We aimed to address this evidence gap by conducting a feasibility RCT of a self-management Smartphone app, "My Journey 3" designed to help EIP service users recognise early warning signs of illness, recognise and monitor symptoms and create plans for their recovery. The results of the feasibility are a potential step towards a full-scale trial to assess the effectiveness of the intervention.

The objectives of this study were as follows:

1. To determine the acceptability of the My Journey 3 self-management app for use in an EIP service context
2. To determine the feasibility of trial procedures for a definitive trial, including recruitment, intervention enrolment and trial attrition.
3. To test procedures for evaluating intervention engagement and participant outcomes.

METHODS

Design

The App to support Recovery in Early Intervention Services (ARIES) study was an unblinded feasibility RCT with a nested qualitative study comparing a self-management Smartphone app (My Journey 3) in addition to Treatment As Usual (TAU), with a control group receiving TAU only. Participants were randomly allocated to one of the two trial arms in a 1:1

For peer review only

ratio. Since this was a feasibility trial, it was not designed to have sufficient statistical power to assess the effectiveness of the My Journey 3 intervention.

Ethical approval was obtained from the London Brent National Research Ethics Service Committee (Ref 15/LO/1453). The trial was retrospectively registered (ISRCTN10004994). As the study was a feasibility trial prospective registration was not required.[29] Further details of the methodology are available in the protocol paper.[30] We have followed the Consolidated Standards of Reporting Trials (CONSORT) statement extension for pilot and feasibility randomised trials for reporting.[31] A copy of the CONSORT checklist is provided as Additional file 1.

Setting

The trial was conducted in six EIP services across three NHS Foundation Trusts in England. EIP services are multi-disciplinary community mental health services that provide care coordination to people in the first three years of a first-episode psychosis, focusing on engagement, achieving social and clinical recovery and delivering a full range of pharmacological, psychological and social interventions.[32] Two of the participating Trusts are located in inner London. The third Trust is located in a county outside of London with both urban and rural areas. Assessments were conducted face-to-face at EIP services, at participants' homes or at University College London.

Participants

Participants were recruited from the participating EIP services over seven months. We assumed a conservative 40% attrition rate and accordingly set the target sample size as 40 participants to ensure the trial retained twelve completer participants per group (as recommended to assess trial feasibility).[33] Participants were eligible if they were aged ≥16 years, had

For peer review only

experienced at least one episode of psychosis, were currently on the caseload of an EIP service and owned a Smartphone with an Android operating system. People were excluded from the trial if they lacked capacity to consent to participation, were unable to communicate and understand English, or were considered by their EIP service to pose a high risk to researchers during meetings, even on NHS premises. Familiarity and competence in using digital technology or Smartphones was not an eligibility criterion.

Recruitment strategy

Clinicians at the participating EIP services were briefed by the research team, and were asked to make initial contact with eligible EIP service users. Clinicians explained the trial to service users, and enquired whether the service user would be willing to speak to a researcher about participating in the trial. The researcher then made contact with eligible and potentially willing service users, and arranged a face-to-face meeting where the trial was explained further. The researcher provided the trial information sheet (Additional file 2), and assessed the participant's capacity to provide informed consent. Service users had at least 24 hours after receiving the information sheet to consider their participation. Participants then gave written informed consent to take part, prior to completing the baseline assessment. No participants were recruited via online methods.

Randomisation

Following the baseline assessment, participants were randomly allocated in a 1:1 ratio to either the intervention (n=20) or the control group (n=20) by an independent statistician. The treatment group had access to My Journey 3 in addition to TAU, whilst the control group received TAU only. An independent researcher held the allocation list and did not disclose participants'

For peer review only

allocation to the trial researcher until after completion of the baseline assessments, allowing the researcher to remain blinded during recruitment and whilst carrying out the baseline assessments.

Due to the nature of the intervention, participants were not blinded to their group allocation. During the recruitment process, participants would have been aware that My Journey 3 was the intervention of interest. As a single researcher carried out the majority of data collection, it was not practical for the allocation of participants to be concealed from the research team. Participants were informed of their allocation by the researcher via a telephone call.

Interventions

My Journey 3

My Journey 3 is a Smartphone app developed for adults accessing EIP services. The aim of the intervention is to develop users' self-management skills to help them to achieve self-determined recovery goals and avoid future relapses. My Journey 3 is suitable for independent use, but also designed to be used with support of EIP service clinicians who will be able to assist with the completion of the self-management components. It is the developers' aspiration for My Journey 3 to be used initially in collaboration with EIP service clinicians, and for it to support continuing self-management after users have been discharged from EIP services.

The development of My Journey 3 has been through several iterations. The first version (My Journey 1) was created by Surrey and Borders Partnership NHS Foundation Trust with leadership from Sarah Amani, for EIP service users to track their symptoms, set reminders for appointments and share their progress with EIP service clinicians. In developing the current version of My Journey 3 we have drawn on existing paper-and-pen self-management intervention components [34,35] to allow users to track recovery goals and personalise relapse prevention plans

For peer review only

– important cornerstones of illness self-management. The design and the content of My Journey 3 was led by a collaboration of researchers, digital health experts, EIP service clinicians and service users. A private app development company based in the UK (MyOxygen; https://myoxygen.uk) led the technical development of My Journey 3. To limit costs My Journey 3 is only compatible with Smartphones with Android operating systems at this stage of testing

My Journey 3 features four key elements of self-management, an approach with demonstrated efficacy in improving social and clinical outcomes for people with psychosis.[12] Users have the ability to create a relapse prevention plan, where there is the opportunity to identify triggers, early warning signs and personalised coping strategies and create a plan to follow if experiencing a crisis. Users are also able to set recovery goals and identify things they can do to keep well using the 'My Recovery Plan" section. Users can use a tracker to monitor and rate their symptoms and early warning signs over time. Psycho-education on mental health, medication and mental health services is provided in an 'Information' section. To encourage adherence with medication, users are encouraged to log and track their medication in the 'Pill Tracker' section. Users are able to set daily alerts to remind them to log whether they have taken their medication. The key components of My Journey 3 are summarized in Table 1, with further details available in the protocol paper.[30]

Prior to the feasibility trial reported in this paper, My Journey 3 was tested by EIP service users in lab-based usability tests and in a one-month field study. The final content of My Journey 3 was then refined based on feedback from individual interviews with the participating EIP service users and clinicians. No changes were made to the content of My Journey 3 during the feasibility RCT. A major technical update to My Journey 3 was carried out in January 2018 to fix

For peer review only

compatibility issues with older versions of Android operating systems. This did not require any changes to the trial design.

Table 1. Key sections of the My Journey 3 Smartphone app.

Section	Further features	Section purpose
My recovery plan	Things I can do to keep well<br><br>My goals	To encourage users to have regular routines, track activities, set reminders and plan how to achieve long-term goals
My relapse prevention plan	Coping with triggers<br><br>Coping with early warning signs<br><br>Coping with a crisis<br><br>Crisis contacts	To help users' identify, monitor and cope with triggers and early warning signs<br><br>To help users create a "relapse plan" to follow in times of crisis
How are you doing?	My mood<br><br>My early warning signs<br><br>My tracker	For users to monitor symptoms, behaviours and early warning signs and track these experiences over time
Pill tracker		To log whether users' have taken their medication each day
Information	Medication information<br><br>Useful websites<br><br>Emergency services<br><br>Jargon buster	To provide users with useful information and external links on medication and mental health<br><br>To identify local emergency services in a time of crisis<br><br>To provide a glossary of terms that are commonly used in mental health care

For peer review only

Delivery

Following assignment to the treatment group, participants engaged in individual training sessions with a trial researcher and a supporting EIP service clinician. Training sessions were intended to take place within six weeks of the participants' initial baseline assessment, and lasted for approximately 2 hours. During these sessions the researcher downloaded My Journey 3 onto the participants' Smartphone and gave a demonstration of the app and its main functions. Participants were then encouraged to input appropriate information to specific sections of My Journey 3 with the help of the supporting EIP service clinician. Following this session it was hoped that all participants had initial personal recovery plans, relapse prevention plans and crisis plans stored on My Journey 3.

Participants had access to My Journey 3 on their own Smartphone from the training session till the 12-month time-point. Researchers recommended that participants used My Journey 3 at least once a week, but participants had a free choice in how and when they used My Journey 3. Participants did not receive any financial incentives to use My Journey 3, and were free to withdraw from using the app or decline the installation of it on to their Smartphone. At the training session participants were informed by the researcher that My Journey 3 would be not suitable for seeking urgent medical care whilst in crisis, and that it is not a substitute for human support.

To encourage user engagement with My Journey 3 during the trial, supporting EIP service clinicians were asked to provide regular support and encouragement to service users who had access to My Journey 3. Clinicians were asked to discuss recovery goals and relapse prevention plans in routine appointments with participants, and assist with entering these into the appropriate My Journey 3 sections. Clinicians had an existing understanding of self-management approaches from their clinical training and practice, and would be able to provide appropriate advice with the

For peer review only

intervention components of My Journey 3. Clinicians' understanding of operating My Journey 3 was from the training sessions only. Clinician support for My Journey 3 as part of the trial was not manualised or incentivised.

Participants were encouraged to contact the trial researcher in the case of technical problems with My Journey 3. The researcher contacted participants a week after the training session to check that My Journey 3 has been functioning without issues, and invited any questions about the app. No further prompts were instigated by the researcher during the trial.

Treatment as usual

All participants received TAU regardless of group allocation. TAU for a person under the care of EIP services typically involves regular meetings with a care co-ordinator, access to a psychiatrist, psychiatric medication, and a range of psychological interventions. EIP services are encouraged to deliver self-management programmes, that includes advice on symptom management, crisis planning and relapse prevention, generally delivered with paper-and-pen tools if at all.[32] None of the participating EIP services offered digital interventions or Smartphone apps as part of routine care during the study period, and structured self-management support, including the relapse prevention work recommended in EIP contexts, was inconsistently implemented.

Patient and Participant Involvement

The development of My Journey 3 has been guided by the input of people with lived experience of psychosis. Initial development of the design and content involved a collaboration between researchers, experts in digital health and service users. Service users provided further

For peer review only

input into the design and functionality of My Journey 3 from providing feedback after taking part in lab-based tests and a field study.

Outcomes

Participant data were collected from numerous sources including participant assessments, patient records and anonymous My Journey 3 usage reports. There were no pre-specified criteria for assessing trial feasibility and intervention acceptability.

Questionnaire measures

Proposed outcome measures for a future trial were assessed at structured face-to-face assessments with a trained researcher at three time points; baseline, 4-months post baseline and 12-months post baseline. At all meetings participants completed self-report questionnaires that have been previously used with people with first-episode psychosis. Participants were given £20 as a thank you for completing the assessment at each time point.

At each assessment we collected sociodemographic data including age, gender, ethnicity, accommodation and living situation, employment status, educational attainment, Smartphone use, and use of other mental health apps. The following self-report measures were also collected: social outcomes (Social Outcomes Index (SIX),[36] score 0-6: higher score= better social outcomes), self-efficacy (Mental Health Confidence Scale (MHCS),[37] score 16-96: higher score= greater empowerment), self-rated recovery (Questionnaire about the Process of Recovery (QPR),[38] intrapersonal score 0-68, interpersonal score 0-20: higher score= greater recovery), mental well-being (Warwick-Edinburgh Mental Well-Being Scale (WEMWBS),[39] score 14-70: higher score= greater well-being) and quality of life and satisfaction with treatment (DIALOG scale,[40] score 1-7: higher score= greater quality of life/satisfaction with treatment).

For peer review only

Clinical structured interviews were also conducted with each participant by the researcher, to assess psychopathology, using the Positive and Negative Syndrome Scale (PANSS).[41] Higher PANSS scores are indicative of greater severity of each symptom domain.

Participants' engagement with EIP services were measured using the Service Engagement Scale (SES),[42] completed by EIP service clinicians known to each participant. Clinicians completed the SES at baseline and 12 months later, regardless of whether participants attended the 12-month assessment. Higher SES scores are indicative of poorer user engagement with EIP services.

Patient records

Clinical data were extracted from patient records at baseline and at the 12-month time point. Clinical measures included most recent diagnosis, most recent care cluster, and use of EIP services, other community mental health teams and acute mental health services in the previous 12 months.

The proposed primary outcome for a future RCT (relapse of psychosis) was operationalised as an admission to an acute mental health service (inpatient psychiatric ward, crisis house, crisis resolution team or acute day care service) during the 12-month trial period as indicated in patient records. This definition of relapse has been used previously in a recent trial of a self-management intervention.[43]

My Journey 3 use

To assess acceptability of the intervention and user engagement, My Journey 3 usage data were collected for all participants in the treatment group from the training session until the 12-month time-point. Whenever users had Wi-Fi internet access on their Smartphone My Journey 3

For peer review only - http://bmjopen.bmj.com/site/about/guidelines.xhtml

automatically uploaded encrypted usage data to a secure server. Data collected included a record of each time the user opened My Journey 3, whether this was in response to a prompt, and which components they used. To ensure confidentiality, personal or identifiable data such as text or responses to each sections were not collected.

Analysis

Participant demographic and clinical characteristics, My Journey 3 usage, and rates of participant recruitment and retention were summarised using descriptive statistics. As this was a feasibility RCT, it was not powered to assess the effectiveness of the intervention. Statistical analyses of participant outcome measures were conducted to pilot the methods of analysis for a fully powered effectiveness trial. Logistic regression was used to explore the impact of the My Journey 3 intervention on relapse. Linear regression was used to examine the potential effect of the intervention on continuous outcome measures at 4 months and 12 months separately. We report the effect estimates and corresponding 95% confidence intervals (CI) only for unadjusted analyses and for analyses adjusting for the baseline measure of the outcome in question. All analyses were performed using STATA V.14 after completion of the final participant assessment. No interim analyses were conducted.

RESULTS

Feasibility of trial design

Participant flow is detailed in the CONSORT diagram (figure 1). A total of 40 participants was recruited and randomised (20 to My Journey 3, 20 to TAU) over a 7-month period from March 2017 to September 2017. Participants were recruited until the required number of 40 was obtained: we do not therefore have a full assessment of the proportion of the teams' caseload who could have

For peer review only

been recruited to a full trail, nor do we know the proportion of approached EIP services users that did not meet eligibility criteria or declined involvement in the trial.

Among those recruited to the trial, attrition rates were generally low: 83% (33/40) and 75% (30/40) of participants successfully attended and completed follow-ups at 4 months and 12 months respectively. At both time points the follow-up rate was lower in the control group (4-months: 65% compared to 100%, 12-months: 70% compared to 80%). Patient record data were available for all participants at baseline and for 95% of the sample (38/40) at the 12-month time-point. Completion rates of the SES by clinicians were higher at baseline (90%) than at the 12-month time-point (67.5%). Follow-up assessments were conducted from July 2017 to October 2018.

All participants in the treatment group attended a training session with a researcher, and had access to My Journey 3 during the trial. Issues with Smartphone compatibility initially prevented three participants from downloading My Journey 3. Following an update to the system two of the participants were able to install and access My Journey 3 on their own Smartphones. Two participants were provided with Smartphones with My Journey 3 pre-installed (the app was still incompatible on one participant's Smartphone despite the update; another participant no longer owned an Android Smartphone after entering the trial). The median length of time from trial enrolment to having access to My Journey 3 was 14 weeks (IQR 11 to 17), longer than the planned time of 6 weeks. Participants had access to My Journey 3 for a median of 38.1 weeks (IQR 34.8 to 40.7). There were no reported privacy breaches.

My Journey 3 usage data were collected for all participants following the training session, with 500 different data entries available for analysis. Within the 500 data entries, 27 (5.4%) were corrupt and were subsequently removed from the analysis. The unusable data can grouped into two types. The first, duplicates of previous data entries that were subsequently removed. The

For peer review only

second, entries where the times were implausible (for example, the end time of using My Journey 3 was recorded as occurring before the start time). In addition, a further issue caused errors with accurately recording My Journey 3 usage data of 'My Recovery Plan' and 'My Relapse Plan' sections. As a result we were unable to accurately conclude how often participants used these sections.

One participant randomised to the control group was wrongly given access to My Journey 3. For the purpose of the statistical analysis they are classed as a control participant.

For peer review only

Figure 1. CONSORT diagram of the ARIES feasibility trial.

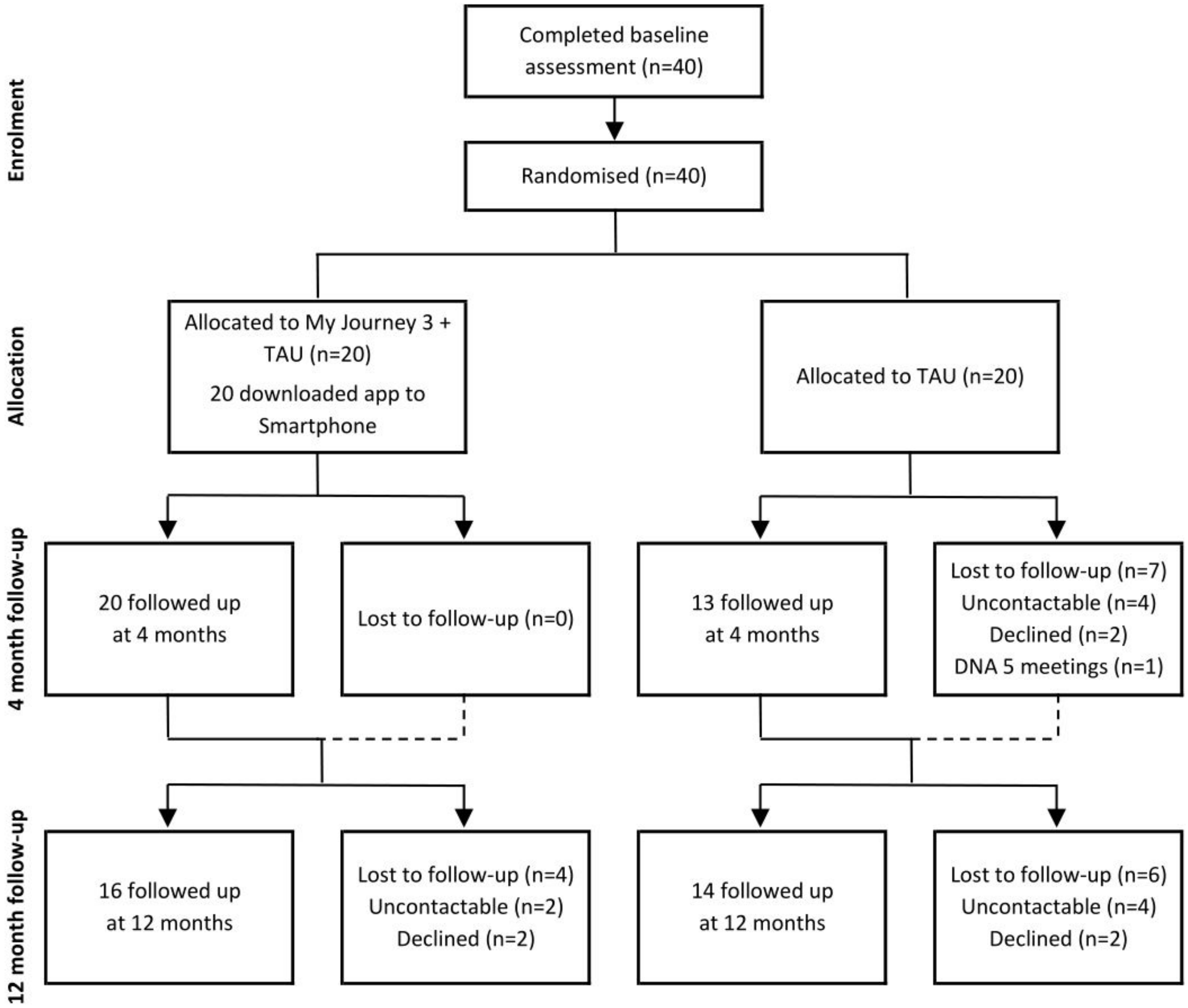

Note: DNA, did not attend.

Sample characteristics

A summary of demographic and clinical characteristics of the sample is displayed in Table 2. The sample was predominantly male (n=28, 70%). Most participants had a diagnosis of a schizophrenia, schizotypal or delusional disorder (ICD code F20-F29) and were not in paid employment. A quarter of the sample (n=10, 25%) had completed a university degree. Eight (20%) participants had previously used a mental health app.

Table 2. Key demographic and clinical characteristics of the sample at baseline.

	Control (n=20)	My Journey 3 (n=20)
**Age (years) – mean (SD)**	30 (10.1)	29.4 (9.7)
**Gender**		
Female	7 (35%)	5 (25%)
**Ethnicity**		
White British	6 (30%)	8 (40%)
Any other white/Mixed white	2 (10%)	1 (5%)
Black African	5 (25%)	3 (15%)
Black Caribbean	1 (5%)	1 (5%)
Black Other	1 (5%)	0
Asian Indian	1 (5%)	0
Asian Other	1 (5%)	2 (10%)
Other/Mixed other	3 (15%)	3 (15%)
**Education**		
Undergraduate degree	6 (30%)	4 (20%)
Some University but no degree	3 (15%)	2 (10%)
Higher National Degree or professional qualification	2 (10%)	1 (5%)
A Levels or equivalent	3 (15%)	4 (20%)
GCSEs or equivalent	4 (20%)	6 (30%)
No qualifications	1 (5%)	3 (15%)
Missing	1 (5%)	0
**Employment status**		
Employed – more than 16 hours a week	4 (20%)	4 (20%)
Employed – less than 16 hours a week	0	2 (10%)
Voluntary work	3 (15%)	3 (15%)
In study or training	1 (5%)	1 (5%)
Unemployed or exempt due to disability	8 (40%)	8 (40%)
Missing	4 (20%)	2 (10%)
**Primary diagnosis (ICD-10 code)**		
F10-F19: Mental and behavioural disorder due to psychoactive substance use	1 (5%)	0
F20-F29: Schizophrenia, schizotypal and delusional disorder	16 (80%)	13 (65%)
F30-F39: Mood disorder	1 (5%)	5 (25%)
Missing	2 (10%)	2 (10%)
**Admission to an acute mental health service in previous year**		
Yes	11 (55%)	10 (50%)
**SIX – mean (SD)**	3.2 (1.5)	3.6 (1.5)
**MHCS – mean (SD)**	59.7 (17.8)	61.2 (12.6)
**QPR – mean (SD)**		
Intrapersonal	45.7 (12)	42.2 (10.6)
Interpersonal	13.7 (2.7)	12.9 (3.4)
**WEMWBS – mean (SD)**	43.4 (11.6)	40.3 (10.2)
**DIALOG – mean (SD)**		
Quality of life	4.5 (1)	4.4 (0.8)
Treatment satisfaction	5.4 (0.7)	4.8 (0.7)
**PANSS – mean (SD)**		
Positive	10.9 (5)	11.3 (4.2)
Negative	10.7 (2.5)	11.8 (4.5)
General	26.6 (6)	26.2 (8)
**SES – mean (SD)**	11.3 (7.9)	9.6 (7)

For peer review only

All statistics are reported N (%) unless otherwise specified. Missing data: PANSS scores – one control group participant, SES – three control group participants, one treatment group participant.

My Journey 3 use

The level of My Journey 3 use was highly skewed. The median number of times My Journey 3 was used per participant during the trial was 16.5 (IQR 8.5 to 23). Participants accessed My Journey 3 a median of 3.22% (IQR 1.89 to 6.36) of the days it was available to them, equating to My Journey 3 being used on average once every 31 days (IQR 15.7 to 52.9). Participants spent a median of 26.8 minutes (IQR 18.3 to 57.3) in total using My Journey 3 over the course of the trial. Eight participants (40%) used My Journey 3 for longer than 30 minutes in total.

Five participants (25%) were still using My Journey 3 six months after downloading it, however one participant never used the app after the training session (figure 2). Half of the participants (n=10) stopped using My Journey 3 within the first three months after the training session.

For peer review only

Figure 2. Bar chart displaying how long after the training session participants disengaged with My Journey 3

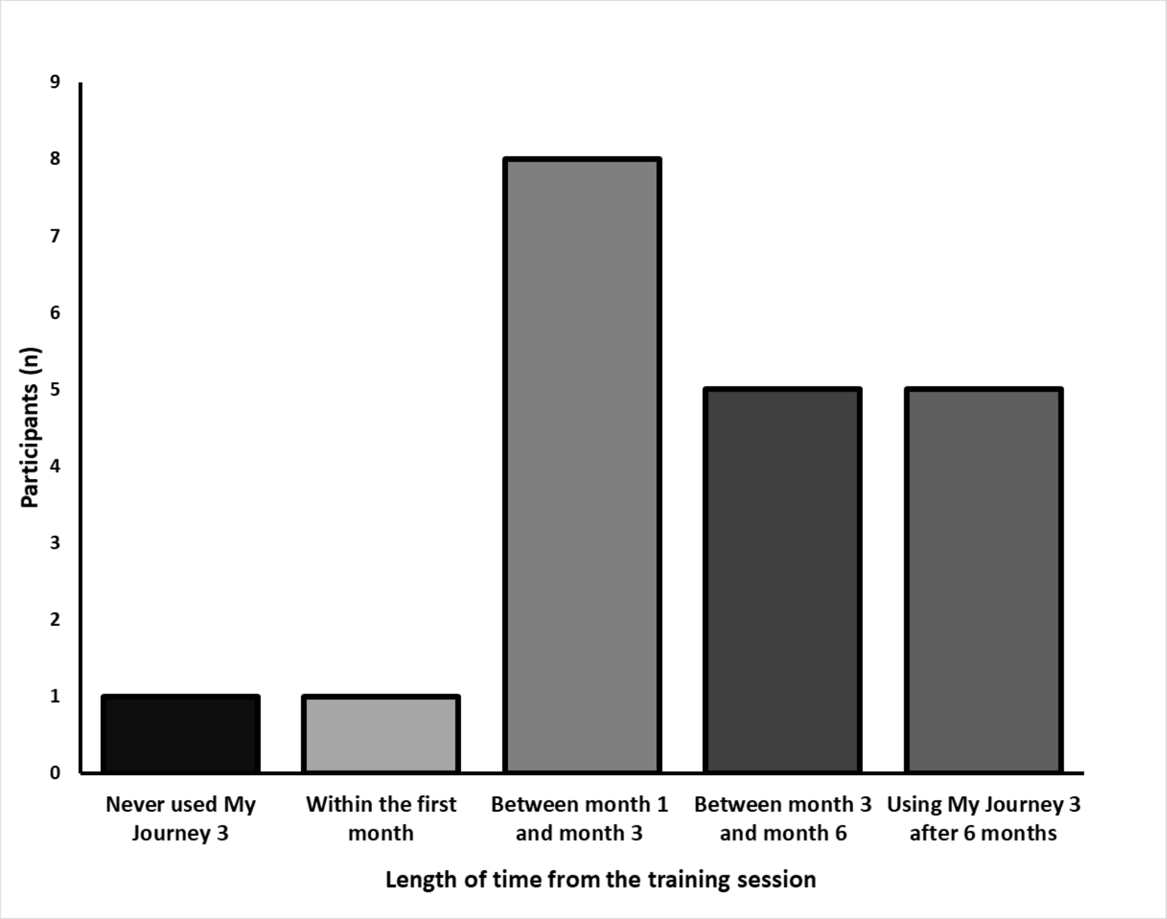

The average number of uses by participants for each My Journey 3 component is displayed in table 3. The most frequently accessed section was the "How are you doing?" section (median uses 3; IQR 1 to 6), however data on how frequently users accessed 'My Recovery Plan' and 'My Relapse Plan' is unavailable. The 'Information' section was accessed the fewest times, with 25% (n=5) of participants in the treatment group never using that section following the training session. Just over 7% of My Journey 3 uses were initiated following a prompt from the app.

For peer review only - http://bmjopen.bmj.com/site/about/guidelines.xhtml

Table 3. Participant use of My Journey 3 and various sections.

	Number of times used per participant	Days used whilst having access to My Journey 3 (%)	Participants that did not use app or section – n (%)
My Journey 3	16.5 (8.5 to 23)	3.22 (1.89 to 6.36)	1 (5%)
How are you doing?	3 (1 to 6)	1.08 (0.4 to 2.12)	3 (15%)
Pill tracker	2 (1 to 3.5)	0.73 (0.36 to 1.07)	3 (15%)
Information	1 (0 to 2.5)	0.48 (0.18 to 0.7)	5 (25%)

All median (IQR), except when stated

Participant outcomes

No research-related serious adverse events were recorded. Psychotic and general symptoms (measured by the PANSS) were generally low at all times for both groups suggesting a stable sample. Summary statistics and estimated effect sizes of participant outcomes are displayed in table 5. Inspection of the effect sizes and confidence intervals suggest that were no obvious differences for any outcome measure between the treatment and control group at either time-point.

Of the 38 participants whose patient records data were available, only five experienced a relapse during the trial, as indicated by using an acute mental health service. In the treatment group 15% of participants (3/20) experienced a relapse during the trial period compared with 11% (2/18) in the control group. We found no evidence of a difference in relapse between the two groups (odds ratio: 1.41; 95% CI: 0.21 to 9.58), but did not have sufficient power for an informative test.

For peer review only

Table 4. Summary statistics and unadjusted and adjusted treatment effects.

	Control (N = 13)	My Journey 3 (N = 20)	Unadjusted analysis		Analysis adjusted for baseline score	
**4-month scores**	Mean (SD)	Mean (SD)	Estimated difference	95% CI	Estimated difference	95% CI
**SIX** (Social Outcomes)	3.3 (1.9)	3.6 (1.3)	0.29	-0.84 to 1.43	0.16	-0.6 to 0.92
**MHCS** (Mental Health Confidence)	66.4 (12.7)	63 (15.8)	-3.43	-14.1 to 7.25	-4.81	-14.88 to 5.25
**QPR** (Recovery)						
Intrapersonal	47.8 (10.6)	43.2 (12.2)	-4.57	-13 to 3.87	-2.01	-8.43 to 4.49
Interpersonal	13.9 (2.4)	13.2 (2.3)	-0.72	-2.39 to 0.95	-0.42	-1.97 to 1.13
**MHCS** (Mental Health Confidence)	46.1 (9.9)	44 (11.3)	-2.08	-9.9 to 5.74	-0.19	-7.28 to 6.9
**DIALOG**						
Quality of life	4.4 (1.2)	4.5 (0.6)	0.07	-0.58 to 0.71	0.18	-0.38 to 0.74
Treatment satisfaction	5.4 (0.7)	5 (0.5)	-0.38	-0.83 to 0.06	-0.17	-0.6 to 0.25
**PANSS** (Symptom severity)						
**Positive**	9.3 (2.9)	11.4 (5.1)	2.09	-1.24 to 5.4	1.9	-0.49 to 4.3
**Negative**	10 (2.3)	11.1 (3.9)	1.05	-1.51 to 3.62	0.54	-1.6 to 2.67
**General**	23 (4)	24 (6.7)	1.21	-3.19 to 5.61	1.35	-2.68 to 5.37
**12-month scores**	Control (N = 14)	My Journey 3 (N = 16)	Unadjusted analysis		Analysis adjusted for baseline score	
	Mean (SD)	Mean (SD)	Estimated difference	95% CI	Estimated difference	95% CI
**SIX** (Social Outcomes)	3.2 (1.9)	3.5 (1.5)	0.29	-0.97 to 1.54	0.29	-0.73 to 1.3
**MHCS** (Mental Health Confidence)	66.2 (14.1)	71.1 (12.1)	4.81	-5 to 14.62	3.03	-6.04 to 12.1
**QPR** (Recovery)						
Intrapersonal	47.3 (11.5)	49.5 (11.1)	2.2	-6.25 to 10.7	3.21	-4.12 to 10.5
Interpersonal	13.6 (3.4)	15.1 (3.3)	1.44	-1.09 to 3.96	1.62	-0.89 to 4.12
**MHCS** (Mental Health Confidence)	45.6 (11.3)	49.3 (9.7)	3.61	-4.24 to 11.46	5.03	-1.67 to 11.7
**DIALOG**						
Quality of life	4.7 (0.9)	5 (0.7)	0.28	-0.31 to 0.87	0.24	-0.33 to 0.81
Treatment satisfaction	5.3 (1)	5.2 (1.2)	-0.12	-0.93 to 0.69	0.31	-0.42 to 1.04
**PANSS** (Symptom severity)						
Positive	9.5 (2.1)	10.2 (2.1)	0.69	-0.98 to 2.36	0.88	-0.62 to 2.38
Negative	10.2 (2.2)	10.9 (3.3)	0.77	-1.51 to 3.05	0.14	-1.56 to 1.84
General	23.5 (5.4)	22.1 (3.5)	-1.38	-4.82 to 2.07	-1	-4.57 to 2.55
**SES** (Engagement with services)	10 (6.2)	9.5 (8)	-0.4	-6.08 to 5.28	3.11	-1.57 to 7.79
**Relapse – n (%)**	2 (11)	3 (15)	OR: 1.41	OR: 0.21 to 9.58		

Missing data: 4-month PANSS scores – one control group participant, one treatment group participant. 12-month PANSS scores – two control group participants. Note: 12-month SES data

For peer review only

available for 13 control group participants, and 14 treatment group participants. Relapse data available for 18 control group participants and 20 treatment group participants. OR; odds ratio.

DISCUSSION

The present study examined the feasibility of conducting a RCT of a supported self-management Smartphone app in EIP services. My Journey 3 aims to facilitate recovery and prevent relapse primarily via the digital delivery of previously developed paper-and-pen self-management tools. The trial indicates that recruitment and retention in a RCT evaluating My Journey 3 is feasible, and that My Journey 3 can be delivered in EIP services. The level of My Journey 3 use was relatively low across the trial period.

Building on from extensive preliminary work with NHS staff and service users, adults with lived experience of psychosis and experts in digital health we were able to successfully develop a self-management Smartphone app that can be used in EIP services. My Journey 3 appeared to be safe with no related serious adverse events reported. My Journey 3 was successfully delivered to all participants in the treatment group, however technical problems with the intervention caused significant delays in providing access. Prior to any future evaluations technical problems with My Journey 3 will need to be identified and fixed to ensure the intervention is implemented as intended.

My Journey 3 use varied considerably between participants, with only a small proportion of participants frequently engaging with the app after obtaining access to it. This raises questions about whether use was at a level where it is likely that useful self-management activities were taking place: certainly not enough time was spent regularly enough for participants to be engaging in detailed monitoring of symptoms and early warning signs, tracking medication and activities

For peer review only

and referring to crisis or recovery plans. Despite that, 40% of participants used My Journey 3 for a minimum of 30 minutes which could be an adequate amount of time for users to effectively monitor relapse signs and follow a crisis plan when needed. We have not found evidence on how regularly EIP service users make use of pen and paper self-management interventions delivered in routine settings, and this was not measured in our trial. Long-term engagement with My Journey 3 appears a challenge, but low levels of app use is a common phenomenon with market research showing that 62% of users stop using Smartphone apps after ten or fewer uses.[44] We will report separately on qualitative findings from this study exploring further the acceptability of My Journey 3 and drivers of engagement and non-adherence.

Participant retention for research data collection was high, with 75% of the sample attending the 12-month follow-up assessment, and is comparable to other Smartphone app studies.[45] Completion rates of the SES by EIP service clinicians were much lower at the 12-month follow in comparison to baseline, potentially due to staff changes and participants being discharged from services. Recruitment strategies were largely successful, however data is lacking on overall proportion of caseload recruited, reasons for non-inclusion and the numbers that were assessed for eligibility, thus limiting the conclusions we can make regarding trial feasibility.

The trial was not powered to detect effectiveness, and, as expected with our small number of participants, we found no significant differences between groups on any outcomes, with confidence intervals generally including substantial effects in either direction. Accordingly we cannot draw any conclusions regarding the potential impact of My Journey 3 as a mental health intervention. The proposed primary outcome for a full-scale trial, relapse as defined by use of an acute mental health service during the trial period, was marked by low event rates. Only five participants (12.5%) experienced a relapse during the one year follow-up period, compared with

For peer review only

expected levels of 12 to 47%.[46] Consideration should be given to whether relapse is an appropriate outcome for a future RCT of this intervention.

Strengths and limitations

My Journey 3 has been developed with extensive stakeholder input, and the intervention has been tested through lab-testing and a field study prior to the feasibility RCT. In comparison to previous studies,[45] participants had access to the app for a longer period of time. Participants' app use and usage data may be more reflective of real-world use as a result. Participant data were also collected from a wide range of methods including from participant assessments and patient records. The proposed primary outcome for a future RCT (relapse) was measured objectively and data were obtained for 95% of participants.

We recruited until the required number of participants was obtained rather than screening caseloads objectively: as a result we are not aware of the proportion eligible who were recruited, reasons for non-eligibility and how many EIP service users declined to take part and why. This limits our understanding of how feasible conducting a large scale trial of this intervention would be. In addition there were issues with the usage data, which impacts the reliability of our conclusions regarding how often participants engaged with My Journey 3.

The trial did not feature an active digital placebo for the control group, meaning that non-specifics of Smartphone use could not be controlled for. Although clinicians were encouraged to support participants with My Journey 3, support was not manualised and clinicians did not have own personal access to the app, potentially limiting the level and quality of the support offered. We did also not define pre-specified criteria for assessing the feasibility of a RCT and the acceptability of My Journey 3. Although the trial was not designed to assess intervention

For peer review only

effectiveness, participants and trial researchers were not blinded to group allocation, and as such could have led to an inflation of any observed effects.

Finally the sample consisted of Android Smartphone users who were generally stable and in an appropriate stage of recovery to consider using a self-management Smartphone app. Participants may therefore not be representative of all EIP service users. Furthermore contact with a researcher within a trial context could have led to increased intervention engagement that would not occur in a real-world clinical environment.

Conclusions

We developed and delivered a self-management Smartphone app for first-episode psychosis in a trial context. Participants were successfully recruited, most engaged at least to some extent with the intervention, and they had high follow-up rates over the one year trial period. Based on the data presented the trial methods appear feasible. My Journey 3 was shown to be safe, but the level of use was lower than anticipated thus potentially limiting its utility, although usage levels were higher than reported for downloaded apps in the general population.

If My Journey 3 is to be further tested in a research setting, attention needs to be given to engagement, a challenge associated with many digital tools in mental health.[47] Further usability testing in lab and field settings may also be a means to improving engagement. Other potential strategies including making more efforts to engage clinicians as well as service users with My Journey 3 by giving them access to the tool and to aspects of the planning and monitoring that service users conduct through it. The app could also potentially be offered as part of a blended approach to self-management, with pen and paper tools also used and as a whole service strategy for implementation of self-management. Refinements required before participating to a full trial

For peer review only

including participant and assessor blinding and manualised clinician support should be considered prior to conducting a future RCT.

For peer review only

Acknowledgements: The ARIES research team are grateful to their software collaborators MyOxygen for their technical development and hosting of My Journey 3 and to Ali Mousa for his valuable contribution to the development of the original My Journey app. We are grateful to Max Birchwood for his permission to incorporate 'Back in the Saddle' in to My Journey 3. We are grateful to Rachel Perkins for her permission to adapt the Personal Recovery Plan resource and incorporate in to My Journey 3.

Author Contributions: SJ is the Chief Investigator, based at University College London, DO the co-Chief Investigator, and TS the project manager. The trial design was developed by SJ, DO, BLE and PO. SA, HR, PO and ME have led on the development of the intervention. TS conducted the statistical analysis, with advice from RJ. TS wrote the draft of the paper, which was revised and approved by all authors. All authors approved the final manuscript.

Funding: The research is funded by the National Institute for Health Research (NIHR) Collaboration for Leadership in Applied Health Research and Care North Thames at Barts Health NHS Trust (NIHR CLAHRC North Thames). The views expressed in this article are those of the authors and not necessarily those of the NHS, the NIHR or the Department of Health and Social Care. SJ, DO and BLE are supported by the NIHR Mental Health Research Policy Unit, the NIHR Collaboration for Leadership in Applied Health Research and Care (CLAHRC) North Thames and the UCLH Biomedical Research Centre.

Data statement: The datasets generated during and/or analysed during the current study will be made available two years after the trial end.

Competing interests: None declared.

For peer review only

Legends:

Figure 1. CONSORT diagram of the ARIES feasibility trial.

Figure 2. Bar chart displaying how long after the training session participants disengaged with My Journey 3.

For peer review only

REFERENCES

1 Bird V, Premkumar P, Kendall T, et al. Early intervention services, cognitive–behavioural therapy and family intervention in early psychosis: systematic review. *Brit J Psychiat*. 2010;197:350-6.

2 Tsiachristas A, Thomas T, Leal J, et al. Economic impact of early intervention in psychosis services: results from a longitudinal retrospective controlled study in England. *BMJ Open* 2016;6:e012611.

3 Alvarez-Jiménez M, Parker AG, Hetrick SE, et al. Preventing the second episode: a systematic review and meta-analysis of psychosocial and pharmacological trials in first-episode psychosis. *Schizophrenia Bull*. 2009;37:619-30.

4 Kam SM, Singh SP, Upthegrove R. What needs to follow early intervention? Predictors of relapse and functional recovery following first-episode psychosis. *Early Interv Psychia*. 2015;9:279-83.

5 Puntis S, Oke J, Lennox B. Discharge pathways and relapse following treatment from early intervention in psychosis services. *BJPsych open* 2018;4:368-74.

6 Leucht S, Heres S. Epidemiology, clinical consequences, and psychosocial treatment of nonadherence in schizophrenia. *J Clin Psychiatry* 2006;67:3-8.

7 Haddock G, Eisner E, Boone C, et al. An investigation of the implementation of NICE-recommended CBT interventions for people with schizophrenia. *J Ment Health* 2014:1;23:162-5.

8 Mueser KT, Meyer PS, Penn DL, et al. The Illness Management and Recovery program: rationale, development, and preliminary findings. *Schizophrenia Bull* 2006;32:S32-43.

For peer review only - http://bmjopen.bmj.com/site/about/guidelines.xhtml

9 Mueser KT, Corrigan PW, Hilton DW et al. Illness management and recovery: A review of the research. *Psychiat Serv* 2002;53:1272-84.

10 Cook JA, Copeland ME, Jonikas JA, et al. Results of a randomized controlled trial of mental illness self-management using Wellness Recovery Action Planning. *Schizophrenia Bull* 2011;38:881-91.

11 Färdig R, Lewander T, Melin L, et al. A randomized controlled trial of the illness management and recovery program for persons with schizophrenia. *Psychiat Serv* 2011;62:606-12.

12 Lean M, Fornells-Ambrojo M, Milton A, et al. Self-management interventions for people with severe mental illness: systematic review and meta-analysis. *Brit J Psychiat* 2019;22:1-9.

13 NICE Guidance - Psychosis and schizophrenia in adults: prevention and management. https://www.nice.org.uk/guidance/cg178/ifp/chapter/peer-support-and-self-management [accessed 2019-01-30]

14 Whitehead L, Seaton P. The effectiveness of self-management mobile phone and tablet apps in long-term condition management: a systematic review. *J Med Internet Res* 2016;18:e97.

15 Poushter J. 2016 Smartphone ownership and internet usage continues to climb in emerging economies. URL: http://www.pewglobal.org/2016/02/22/smartphone-ownership-and-internet-usage-continues-to-climb-in-emerging-economies/ [accessed 2019-01-30]

16 Wigginton C, Curran M, Broedeur C. 2017. Global mobile consumer trends, 2nd edition. URL: https://www2.deloitte.com/content/dam/Deloitte/us/Documents/technology-media-telecommunications/us-global-mobile-consumer-survey-second-edition.pdf. [accessed 2019-07-04]

For peer review only

17 Firth J, Cotter J, Torous J, et al. Mobile phone ownership and endorsement of "mHealth" among people with psychosis: a meta-analysis of cross-sectional studies. *Schizophrenia Bull* 2015;42:448-55.

18 Robotham D, Satkunanathan S, Doughty L, Wykes T. Do We Still Have a Digital Divide in Mental Health? A Five-Year Survey Follow-up. *J Med Internet Res* 2016;18:e309.

19 Naslund JA, Aschbrenner KA, Bartels SJ. How people with serious mental illness use smartphones, mobile apps, and social media. *Psychiatr Rehabil J* 2016;39:364-367.

20 Proudfoot J. The future is in our hands: the role of mobile phones in the prevention and management of mental disorders. *Aust NZ J Psychiat* 2013;47:111-3.

21 Gottlieb JD, Romeo KH, Penn DL, et al. Web-based cognitive–behavioral therapy for auditory hallucinations in persons with psychosis: a pilot study. *Schizophr Res* 2013;145:82–87.

22 Granholm E, Ben-Zeev D, Link PC, et al. Mobile Assessment and Treatment for Schizophrenia (MATS): a pilot trial of an interactive text-messaging intervention for medication adherence, socialization, and auditory hallucinations. *Schizophr Bull* 2011;38:414-25.

23 Ben-Zeev D, Kaiser SM, Brenner CJ, et al. Development and usability testing of FOCUS: A smartphone system for self-management of schizophrenia. *Psychiatr Rehabil J* 2013;36:289-296.

24 Alvarez-Jimenez M, Bendall S, Lederman R, et al. On the HORYZON: moderated online social therapy for long-term recovery in first episode psychosis. *Schizophr Res* 2013;143:143-9.

25 Aref-Adib G, O'Hanlon P, Fullarton K, et al. A qualitative study of online mental health information seeking behaviour by those with psychosis. *BMC Psychiatry* 2016;16:232.

For peer review only

26 Berry N, Lobban F, Bucci S. A qualitative exploration of service user views about using digital health interventions for self-management in severe mental health problems. *BMC Psychiatry* 2019;19:35.

27 Bonet L, Izquierdo C, Escartí MJ, et al. Use of mobile technologies in patients with psychosis: A systematic review. *Rev Psiquiatr Salud Ment* 2017;10:168-78.

28 Bucci S, Barrowclough C, Ainsworth J, et al. Actissist: Proof-of-Concept Trial of a Theory-Driven Digital Intervention for Psychosis. *Schizophrenia Bull* 2018; sby032.

29 JMIR. URL: https://support.jmir.org/hc/en-us/articles/115001389307-Does-my-trial-RCT-have-to-be-registered- [accessed 2019-10-08]

30 Steare T, O'Hanlon P, Eskinazi M, et al. App to support Recovery in Early Intervention Services (ARIES) study: protocol of a feasibility randomised controlled trial of a self-management Smartphone application for psychosis. *BMJ Open* 2019;9:e025823.

31 Eldridge SM, Chan CL, Campbell MJ, et al. CONSORT 2010 statement: extension to randomised pilot and feasibility trials. *BMJ* 2016;355:i5239.

32 National Institute for Health and Care Excellence. Implementing the Early Intervention in Psychosis Access and Waiting Time Standard: Guidance. London: NHS England Publications. 2016. p. 1-57.

33 Julious SA. Sample size of 12 per group rule of thumb for a pilot study. *Pharm Stat* 2005;287-91.

34 Plaistow J, Birchwood M. Back in the saddle: a guide to relapse prevention. In: Birchwood M, Fowler D, Jackson C, editors. Early Intervention in Psychosis: A Guide to Concepts, Evidence and Interventions. Chichester: McGraw-Hill; 1996. p. 239-244.

For peer review only

35 Perkins R, Rinaldi M. Taking back control: a guide to planning your own recovery. South West London: St George's Mental Health NHS Trust, 2007.

36 Priebe S, Watzke S, Hansson L, et al. Objective social outcomes index (SIX): a method to summarise objective indicators of social outcomes in mental health care. *Acta Psychiat Scand* 2008;118:57-63.

37 Carpinello SE, Knight EL, Markowitz FE, et al. The development of the Mental Health Confidence Scale: A measure of self-efficacy in individuals diagnosed with mental disorders. *Psychiatr Rehabil J* 2000;23:236-243.

38 Neil ST, Kilbride M, Pitt L, et al. The questionnaire about the process of recovery (QPR): a measurement tool developed in collaboration with service users. *Psychosis* 2009;1:145-55.

39 Tennant R, Hiller L, Fishwick R, et al. The Warwick-Edinburgh mental well-being scale (WEMWBS): development and UK validation. *Health Qual Life Out* 2007;5:63.

40 Priebe S, McCabe RO, Bullenkamp J, et al. Structured patient–clinician communication and 1-year outcome in community mental healthcare. *Brit J Psychiat* 2007;191:420-6.

41 Kay SR, Fiszbein A, Opfer LA. The positive and negative syndrome scale (PANSS) for schizophrenia. *Schizophrenia Bull* 1987;13:261-76.

42 Tait L, Birchwood M, Trower P. A new scale (SES) to measure engagement with community mental health services. *J Ment Health* 2002;11:191-8.

43 Johnson S, Lamb D, Marston L, et al. Peer-supported self-management for people discharged from a mental health crisis team: a randomised controlled trial. *Lancet* 2018;392:409-18.

For peer review only

44 Localytics. 21% of users abandon an app after one use. http://info.localytics.com/blog/21-percent-of-users-abandon-apps-after-one-use. [accessed 2019-05-16]

45 Firth J, Torous J. Smartphone apps for schizophrenia: a systematic review. *JMIR Mhealth Uhealth* 2015;3.

46 Alvarez-Jimenez M, Priede A, Hetrick SE, et al. Risk factors for relapse following treatment for first episode psychosis: a systematic review and meta-analysis of longitudinal studies. *Schizophr Res* 2012;139:116-28.

47 Aref-Adib G, McCloud T, Ross J, et al. Factors affecting implementation of digital health interventions for people with psychosis or bipolar disorder, and their family and friends: a systematic review. *Lancet Psychiat* 2018;6:257-66.

For peer review only

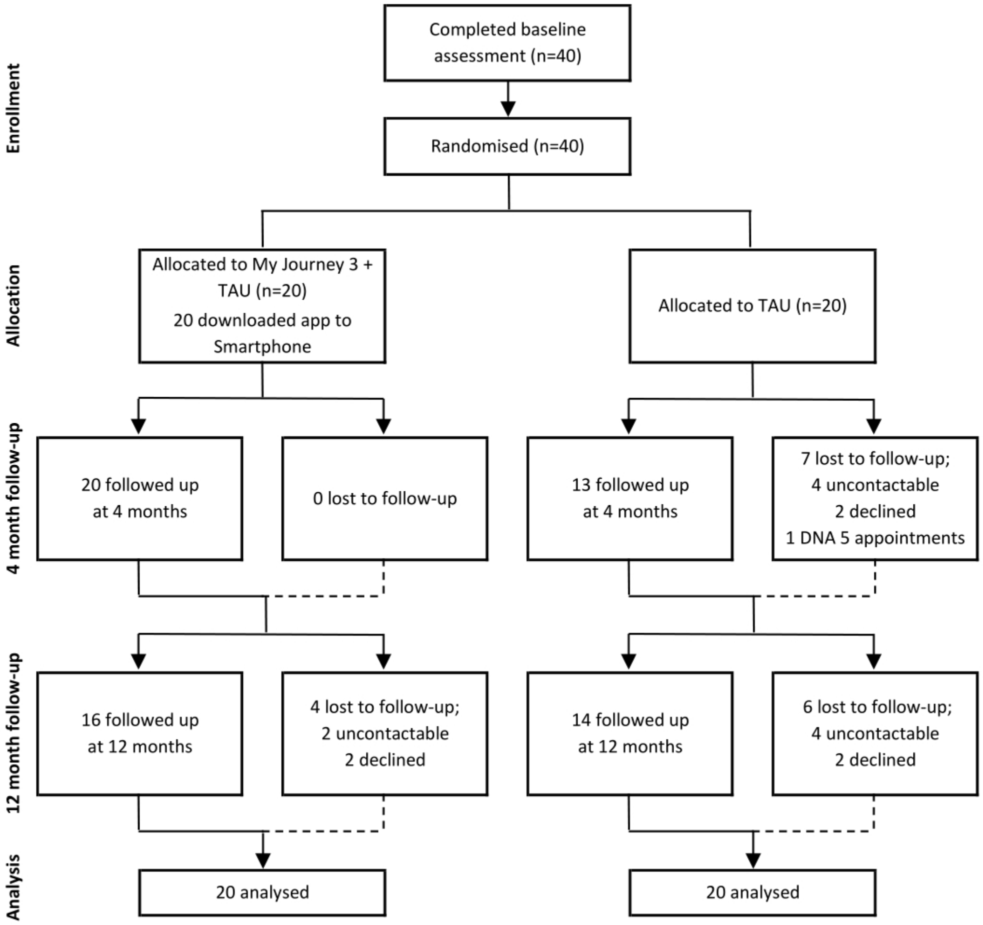

CONSORT diagram of the ARIES feasibility trial.

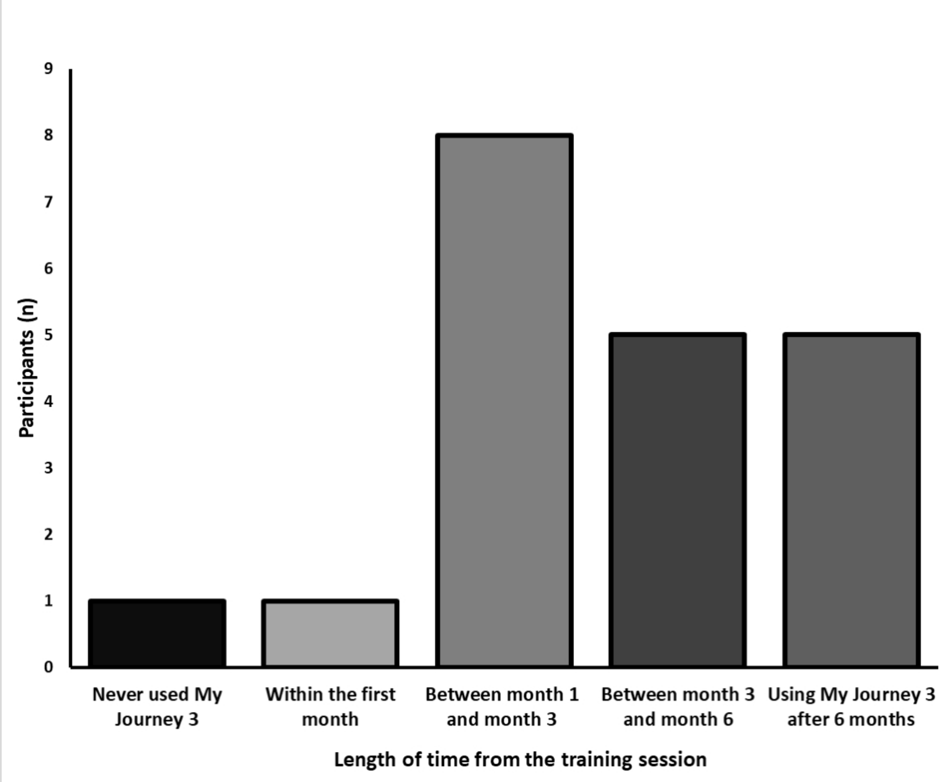

Figure 2. Bar chart displaying how long after the training session participants disengaged with My Journey 3

Service user participant consent form

Study Title: App to support Recovery In Early intervention Services (the ARIES study): Pilot randomised controlled trial of a self-management smartphone application

Principal Investigators: Professor Sonia Johnson and Professor David Osborn

Please Initial Each Box

1. I confirm that I have read and understood the Participant Information Sheet V5 dated 29/05/2017 for the above study and have had the opportunity to ask questions about the study. ☐
2. I understand that my participation is voluntary and that I am free to withhold personal information or to withdraw my participation at any time, without giving any reason, and without my medical care or legal rights being affected. ☐
3. I understand that if I choose to withdraw from the study that any data that I have already provided for the purposes of the research will be kept and used by the research team. ☐
4. I give permission for my General Practitioner (GP) and my Early Intervention team to be told I am participating in this study. ☐
5. I understand that relevant sections of my medical notes and data collected during the study may be looked at by regulatory authorities or from the NHS Trust, where it is relevant to my taking part in this research. I give permission for these individuals to have access to my records. ☐
6. I understand that I will be given a £20 gift as cash for my participation in each study assessment. ☐
7. I agree to the research team consulting NHS electronic records to investigate my diagnosis, medication, and mental health service use, and give them permission to do so even if I choose to no longer participate in the intervention, or they are not able to carry out further study interviews with me. ☐
8. I understand that in the event that I disclose information which may indicate new risk to myself or others, the researcher will be obliged to follow NHS Trust risk procedures that may require release of my personal data. ☐
9. I give permission for findings from the study to be written up for publication. Any publication will not identify me. ☐
10. I give permission to be audio recorded where required for the purposes of the study. I understand these audio-recordings will be transcribed and anonymised and audio recordings destroyed after the study. I give permission for direct quotations taken from this interview to be included in papers written for publication. Any quotation would not identify me. ☐
11. I give permission for the research team to collect data from the My Journey 3 app regarding the frequency, duration, and pattern of my use of it. I understand that no personal information will be collected from the app. ☐
12. I give permission for non-identifiable data to be shared with other research teams for research purposes. ☐

For peer review only - http://bmjopen.bmj.com/site/about/guidelines.xhtml

13. I agree to take part in this study. ☐

Name of participant	Date	Signature
Name of Researcher taking consent	Date	Signature

For peer review only

For peer review only - http://bmjopen.bmj.com/site/about/guidelines.xhtml

CONSORT 2010 checklist of information to include when reporting a pilot or feasibility trial*

Section/Topic	Item No	Checklist item	Reported on page No
**Title and abstract**			
	1a	Identification as a pilot or feasibility randomised trial in the title	Title Page
	1b	Structured summary of pilot trial design, methods, results, and conclusions (for specific guidance see CONSORT abstract extension for pilot trials)	Abstract
**Introduction**			
Background and objectives	2a	Scientific background and explanation of rationale for future definitive trial, and reasons for randomised pilot trial	Introduction
	2b	Specific objectives or research questions for pilot trial	Introduction
**Methods**			
Trial design	3a	Description of pilot trial design (such as parallel, factorial) including allocation ratio	Methods (design)
	3b	Important changes to methods after pilot trial commencement (such as eligibility criteria), with reasons	N/A
Participants	4a	Eligibility criteria for participants	Methods (participants)
	4b	Settings and locations where the data were collected	Methods (setting)
	4c	How participants were identified and consented	Methods (recruitment strategy)
Interventions	5	The interventions for each group with sufficient details to allow replication, including how and when they were actually administered	Methods (interventions)
Outcomes	6a	Completely defined prespecified assessments or measurements to address each pilot trial objective specified in 2b, including how and when they were assessed	Methods (outcomes)
	6b	Any changes to pilot trial assessments or measurements after the pilot trial commenced, with reasons	N/A
	6c	If applicable, prespecified criteria used to judge whether, or how, to proceed with future definitive trial	N/A
Sample size	7a	Rationale for numbers in the pilot trial	Methods (participants)

For peer review only

	7b	When applicable, explanation of any interim analyses and stopping guidelines	Methods (analysis)
Randomisation:			
Sequence generation	8a	Method used to generate the random allocation sequence	Methods (randomisation)
	8b	Type of randomisation(s); details of any restriction (such as blocking and block size)	Methods (randomisation)
Allocation concealment mechanism	9	Mechanism used to implement the random allocation sequence (such as sequentially numbered containers), describing any steps taken to conceal the sequence until interventions were assigned	Methods (randomisation)
Implementation	10	Who generated the random allocation sequence, who enrolled participants, and who assigned participants to interventions	Methods (randomisation)
Blinding	11a	If done, who was blinded after assignment to interventions (for example, participants, care providers, those assessing outcomes) and how	Methods (randomisation)
	11b	If relevant, description of the similarity of interventions	N/A
Statistical methods	12	Methods used to address each pilot trial objective whether qualitative or quantitative	Methods (analysis)
**Results**			
Participant flow (a diagram is strongly recommended)	13a	For each group, the numbers of participants who were approached and/or assessed for eligibility, randomly assigned, received intended treatment, and were assessed for each objective	Results (feasibility of trial design)
	13b	For each group, losses and exclusions after randomisation, together with reasons	Results (feasibility of trial design)
Recruitment	14a	Dates defining the periods of recruitment and follow-up	Results (feasibility of trial design)
	14b	Why the pilot trial ended or was stopped	N/A
Baseline data	15	A table showing baseline demographic and clinical characteristics for each group	Results (sample characteristics)
Numbers analysed	16	For each objective, number of participants (denominator) included in each analysis. If relevant, these numbers should be by randomised group	Results (participant outcomes)

For peer review only

Outcomes and estimation	17	For each objective, results including expressions of uncertainty (such as 95% confidence interval) for any estimates. If relevant, these results should be by randomised group	Results (participant outcomes)
Ancillary analyses	18	Results of any other analyses performed that could be used to inform the future definitive trial	N/A
Harms	19	All important harms or unintended effects in each group (for specific guidance see CONSORT for harms)	Results (participant outcomes)
	19a	If relevant, other important unintended consequences	N/A
**Discussion**			
Limitations	20	Pilot trial limitations, addressing sources of potential bias and remaining uncertainty about feasibility	Discussion (strength and limitations)
Generalisability	21	Generalisability (applicability) of pilot trial methods and findings to future definitive trial and other studies	Discussion (strength and limitations)
Interpretation	22	Interpretation consistent with pilot trial objectives and findings, balancing potential benefits and harms, and considering other relevant evidence	Discussion (conclusions)
	22a	Implications for progression from pilot to future definitive trial, including any proposed amendments	Discussion (conclusions)
**Other information**			
Registration	23	Registration number for pilot trial and name of trial registry	Abstract
Protocol	24	Where the pilot trial protocol can be accessed, if available	Additional file 3
Funding	25	Sources of funding and other support (such as supply of drugs), role of funders	Funding
	26	Ethical approval or approval by research review committee, confirmed with reference number	Methods (design)

Citation: Eldridge SM, Chan CL, Campbell MJ, Bond CM, Hopewell S, Thabane L, et al. CONSORT 2010 statement: extension to randomised pilot and feasibility trials. BMJ. 2016;355.
*We strongly recommend reading this statement in conjunction with the CONSORT 2010, extension to randomised pilot and feasibility trials, Explanation and Elaboration for important clarifications on all the items. If relevant, we also recommend reading CONSORT extensions for cluster randomised trials, non-inferiority and equivalence trials, non-pharmacological treatments, herbal interventions, and pragmatic trials. Additional extensions are forthcoming: for those and for up to date references relevant to this checklist, see www.consort-statement.org.

For peer review only

BMJ Open

Smartphone-delivered self-management for first-episode psychosis: the ARIES feasibility randomised controlled trial

Journal:	*BMJ Open*
Manuscript ID	bmjopen-2019-034927.R1
Article Type:	Original research
Date Submitted by the Author:	08-Jan-2020
Complete List of Authors:	Steare, Thomas; University College London, Division of Psychiatry<br>O'Hanlon, Puffin; University College London, Division of Psychiatry<br>Eskinazi, Michelle; University College London, Division of Psychiatry<br>Osborn, David; University College London, Division of Psychiatry; Camden and Islington NHS Foundation Trust<br>Lloyd-Evans, Brynmor ; University College London, Division of Psychiatry; Camden and Islington NHS Foundation Trust<br>Jones, Rebecca; University College London, Division of Psychiatry<br>Rostill, Helen; University of Surrey; Surrey and Borders Partnership NHS Foundation Trust<br>Amani, Sarah; Early Intervention in Psychosis Programme (South of England)<br>Johnson, Sonia; University College London, Division of Psychiatry; Camden and Islington NHS Foundation Trust
**Primary Subject Heading**:	Mental health
Secondary Subject Heading:	Health services research
Keywords:	Schizophrenia & psychotic disorders < PSYCHIATRY, MENTAL HEALTH, Clinical trials < THERAPEUTICS

SCHOLARONE™
Manuscripts

I, the Submitting Author has the right to grant and does grant on behalf of all authors of the Work (as defined in the below author licence), an exclusive licence and/or a non-exclusive licence for contributions from authors who are: i) UK Crown employees; ii) where BMJ has agreed a CC-BY licence shall apply, and/or iii) in accordance with the terms applicable for US Federal Government officers or employees acting as part of their official duties; on a worldwide, perpetual, irrevocable, royalty-free basis to BMJ Publishing Group Ltd ("BMJ") its licensees and where the relevant Journal is co-owned by BMJ to the co-owners of the Journal, to publish the Work in this journal and any other BMJ products and to exploit all rights, as set out in our licence.

The Submitting Author accepts and understands that any supply made under these terms is made by BMJ to the Submitting Author unless you are acting as an employee on behalf of your employer or a postgraduate student of an affiliated institution which is paying any applicable article publishing charge ("APC") for Open Access articles. Where the Submitting Author wishes to make the Work available on an Open Access basis (and intends to pay the relevant APC), the terms of reuse of such Open Access shall be governed by a Creative Commons licence – details of these licences and which Creative Commons licence will apply to this Work are set out in our licence referred to above.

Other than as permitted in any relevant BMJ Author's Self Archiving Policies, I confirm this Work has not been accepted for publication elsewhere, is not being considered for publication elsewhere and does not duplicate material already published. I confirm all authors consent to publication of this Work and authorise the granting of this licence.

For peer review only

TITLE PAGE

Title: Smartphone-delivered self-management for first-episode psychosis: the ARIES feasibility randomised controlled trial

Authors:

Thomas Steare

Division of Psychiatry, University College London, London, UK

Puffin O'Hanlon

Division of Psychiatry, University College London, London, UK

Michelle Eskinazi

Division of Psychiatry, University College London, London, UK

David Osborn

Division of Psychiatry, University College London, London, UK

R&D Department, Camden and Islington NHS Foundation Trust, London, UK

Brynmor Lloyd-Evans

Division of Psychiatry, University College London, London, UK

R&D Department, Camden and Islington NHS Foundation Trust, London, UK

Rebecca Jones

Division of Psychiatry, University College London, London, UK

Helen Rostill

University of Surrey, UK

Surrey and Borders Partnership NHS Foundation Trust, Leatherhead, Surrey, UK

Sarah Amani

EIP Programme (South of England), NHS England, Oxford, Oxfordshire, UK

For peer review only

Sonia Johnson (corresponding author)

Address: UCL Division of Psychiatry, 6th Floor, Wing B, Maple House, 149 Tottenham Court Road, London W1T 7NF

Division of Psychiatry, University College London, London, UK

R&D Department, Camden and Islington NHS Foundation Trust, London, UK

s.johnson@ucl.ac.uk

02076799453

Word count: 5,790

Keywords: Psychosis, Self-management, Smartphone, Feasibility study, Randomised controlled trial

For peer review only

ABSTRACT

Objectives: To test the feasibility and acceptability of a randomised controlled trial (RCT) to evaluate a Smartphone-based self-management tool in Early Intervention in Psychosis (EIP) services.

Design: A two-arm unblinded feasibility RCT.

Setting: Three NHS EIP services in England.

Participants: Adults using EIP services that own an Android Smartphone. Participants were recruited until the recruitment target was met (n=40).

Interventions: Participants were randomised with a 1:1 allocation to one of two conditions: (1) treatment as usual from EIP services (TAU) or (2) TAU plus access to My Journey 3 on their own Smartphone. My Journey 3 features a range of self-management components including access to digital recovery and relapse prevention plans, medication tracking and symptom monitoring. My Journey 3 use was at the users' discretion, and was supported by EIP service clinicians. Participants had access for a median of 38.1 weeks.

Primary and secondary outcome measures: Feasibility outcomes included recruitment, follow-up rates and intervention engagement. Participant data on mental health outcomes were collected from clinical records and from research assessments at baseline, 4 months and 12 months.

Results: 83% and 75% of participants were retained in the trial at the 4- and 12-month assessments. All treatment group participants had access to My Journey 3 during the trial, but technical difficulties caused delays in ensuring timely access to the intervention. The median

For peer review only

number of My Journey 3 uses was 16.5 (IQR 8.5 to 23) and median total minutes spent using My Journey 3 was 26.8 (IQR 18.3 to 57.3). No serious adverse events were reported.

Conclusions: Recruitment and retention were feasible. Within a trial context My Journey 3 could be successfully delivered to adults using EIP services, but with relatively low usage rates. Further evaluation of the intervention in a larger trial may be warranted, but should include attention to implementation.

Trial Registration: ISRCTN10004994

ARTICLE SUMMARY

Strengths and limitations of this study:

- Participant data was collected from a wide range of sources including questionnaires, patient records and from the app
- Participants were followed up for a 12-month period; longer than the majority of feasibility trials investigating Smartphone apps for psychosis
- We were not able to blind researchers or participants to their treatment allocation
- The study recruited users of Early Intervention in Psychosis services that own an Android Smartphone, limiting sample representativeness
- This is a feasibility study, and therefore does not have the statistical power to conclude the effectiveness of the intervention.

For peer review only

INTRODUCTION

Early Intervention in Psychosis (EIP) services have been established across the United Kingdom to provide care to adults during the three years following an initial episode of psychosis. There is evidence that such services are effective and cost-effective,[1,2] resulting in improvement in a range of outcomes yet challenges remain. Relapse rates for EIP service users are high [3] particularly after discharge [4,5] and limited adherence with antipsychotic medication is common [6]. There are also difficulties accessing psychosocial interventions,[7] including supported self-management.

Illness self-management is an approach designed to support people to manage long-term health conditions by developing their ability to recognise and monitor symptoms and early warning signs of relapse, identify and avoid stressors, make plans for achieving their own recovery and to effectively use coping strategies.[8] For people with psychosis, self-management tools have been shown to reduce psychological distress, improve medication adherence and reduce the likelihood of future hospital admissions.[9-11] In a recent meta-analysis, self-management interventions for severe mental illness were also found to have a significant benefit on patient-valued outcomes of personal recovery, hope and self-efficacy.[12] Despite clinician-supported self-management programmes being mandated in current UK treatment guidelines for first-episode psychosis,[13] there is a lack of well-evaluated tools to support delivery within EIP services. There is a clear need to overcome implementation barriers affecting the delivery of self-management to those likely to benefit from it.[12] A potentially convenient and economical way of achieving this is via the use of digital technology such as Smartphones.[14]

Smartphones can run advanced software known as apps that hold promise as an effective tool to assist the monitoring and treatment of mental health problems. Smartphone ownership is

For peer review only

rapidly growing world-wide [15] with a significant number of developed countries with ownership rates of more than 80%.[16] Adults with severe mental health problems have comparable Smartphone ownership rates to the general population,[17-19] and there is a growing consensus that adults with psychosis are open to using Smartphones to access mental health interventions.[20,21] Smartphones also provide high accessibility to the internet and are commonly carried on the person, meaning apps can be easily accessed at times and locations convenient for the user. Accordingly Smartphones have the capacity to deliver time-unlimited mental health interventions, such as self-management tools, and ultimately the potential to increase access to effective care and reduce healthcare costs.[22] The benefits of Smartphone apps may also extend beyond the original treatment period with a community team, and could be a valuable tool following discharge where the risk of relapse is increased.[4,5]

The majority of digital health interventions that have been developed for psychosis have been based on existing psychological therapies such as Cognitive Behavioural Therapy,[23,24] or other evidence-based interventions,[25,26] yet very little is known regarding their effectiveness when delivered in EIP services. A growing number of self-management apps for psychosis have been tested for feasibility and acceptability, including those delivered independent of a clinical setting and those embedded within clinical care.[27-29] These have shown promising levels of adoption and use in research contexts yet little is known about their clinical efficacy.

To date only one trial of a self-management app delivered in EIP services has published results regarding the interventions impact on clinical outcomes.[30] In the proof-of-concept trial an active self-management app "Actissist" was found to confer benefits over a passive control app. The study suggests that participants that received Actissist had better outcomes regarding their mood and general and negative symptoms post-treatment in comparison to control participants.

For peer review only

Actissist features a range of components including self-assessment questions focused on cognitive appraisals, emotions, behaviours and belief convictions and suggests appropriate coping strategies, but does not feature some major cornerstones of self-management such as relapse and recovery plans. Regardless results from this study suggest that such digital self-management interventions could potentially improve outcomes of people using EIP services. Further trials are needed before firm conclusions can be made regarding the feasibility of conducting RCTs in this field and of the therapeutic benefits of self-management apps for first-episode psychosis delivered in clinical settings.

We aimed to address this evidence gap by conducting a feasibility RCT of a supported self-management Smartphone app, “My Journey 3” designed to help EIP service users recognise early warning signs of illness, recognise and monitor symptoms and create plans for their recovery. My Journey 3 has been designed to be initially set up in EIP services and used with clinician support, but to also be suitable for independent use. The results of the feasibility RCT are a potential step towards a full-scale trial to assess the effectiveness of the intervention.

The objectives of this study were as follows:

1. To determine the acceptability of the My Journey 3 self-management app for use in an EIP service context
2. To determine the feasibility of trial procedures for a definitive trial, including recruitment, intervention enrolment and trial attrition.
3. To test procedures for evaluating intervention engagement and participant outcomes.

For peer review only

METHODS

Design

The App to support Recovery in Early Intervention Services (ARIES) study was an unblinded feasibility RCT with a nested qualitative study comparing a supported self-management Smartphone app (My Journey 3) in addition to Treatment As Usual (TAU), with a control group receiving TAU only. Participants were randomly allocated to one of the two trial arms in a 1:1 ratio. Since this was a feasibility trial, it was not designed to have sufficient statistical power to assess the effectiveness of the My Journey 3 intervention.

Ethical approval was obtained from the London Brent National Research Ethics Service Committee (Ref 15/LO/1453). The trial was retrospectively registered (ISRCTN10004994). As the study was a feasibility trial prospective registration was not required.[31] Further details of the methodology are available in the protocol paper.[32] We have followed the Consolidated Standards of Reporting Trials (CONSORT) statement extension for pilot and feasibility randomised trials for reporting.[33] A copy of the CONSORT checklist is provided as Additional file 1.

Setting

The trial was conducted in six EIP services across three NHS Foundation Trusts in England. EIP services are multi-disciplinary community mental health services that provide care coordination to people in the first three years of a first-episode psychosis, focusing on engagement, achieving social and clinical recovery and delivering a full range of pharmacological, psychological and social interventions.[34] Two of the participating Trusts are located in inner London. The third Trust is located in a county outside of London with both urban and rural areas.

For peer review only

Assessments were conducted face-to-face at EIP services, at participants' homes or at University College London.

Participants

Participants were recruited from the participating EIP services over seven months. We assumed a conservative 40% attrition rate and accordingly set the target sample size as 40 participants to ensure the trial retained twelve completer participants per group (as recommended to assess trial feasibility).[35] Participants were eligible if they were aged ≥16 years, had experienced at least one episode of psychosis, were currently on the caseload of an EIP service and owned a Smartphone with an Android operating system. People were excluded from the trial if they lacked capacity to consent to participation, were unable to communicate and understand English, or were considered by their EIP service to pose a high risk to researchers during meetings, even on NHS premises. Familiarity and competence in using digital technology or Smartphones was not an eligibility criterion.

Recruitment strategy

Clinicians at the participating EIP services were briefed by the research team, and were asked to make initial contact with eligible EIP service users. Clinicians explained the trial to service users, and enquired whether the service user would be willing to speak to a researcher about participating in the trial. The researcher then made contact with eligible and potentially willing service users, and arranged a face-to-face meeting where the trial was explained further. The researcher provided the trial information sheet (Additional file 2), and assessed the participant's capacity to provide informed consent. Service users had at least 24 hours after receiving the information sheet to consider their participation. Participants then gave written

For peer review only

informed consent to take part, prior to completing the baseline assessment. No participants were recruited via online methods.

Randomisation

Following the baseline assessment, participants were randomly allocated in a 1:1 ratio to either the intervention (n=20) or the control group (n=20) by an independent statistician. The treatment group had access to My Journey 3 in addition to TAU, whilst the control group received TAU only. An independent researcher held the allocation list and did not disclose participants' allocation to the trial researcher until after completion of the baseline assessments, allowing the researcher to remain blinded during recruitment and whilst carrying out the baseline assessments.

Due to the nature of the intervention, participants were not blinded to their group allocation. During the recruitment process, participants would have been aware that My Journey 3 was the intervention of interest. As a single researcher carried out the majority of data collection, it was not practical for the allocation of participants to be concealed from the research team. Participants were informed of their allocation by the researcher via a telephone call.

Interventions

My Journey 3

My Journey 3 is a Smartphone app developed for adults accessing EIP services. The aim of the intervention is to develop users' self-management skills to help them to achieve self-determined recovery goals and avoid future relapses. My Journey 3 is suitable for independent use, but also designed to be used with support from EIP service clinicians who will be able to assist with the completion of the self-management components and initial set-up. It is the developers'

For peer review only

aspiration for My Journey 3 to be used initially in collaboration with EIP service clinicians, and for it to support continuing self-management after users have been discharged from EIP services.

The development of My Journey 3 has been through several iterations. The first version (My Journey 1) was created by Surrey and Borders Partnership NHS Foundation Trust with leadership from Sarah Amani, for EIP service users to track their symptoms, set reminders for appointments and share their progress with EIP service clinicians. In developing the current version of My Journey 3 we have drawn on existing paper-and-pen self-management intervention components [36,37] to allow users to track recovery goals and personalise relapse prevention plans – important cornerstones of illness self-management. The design and the content of My Journey 3 was led by a collaboration of researchers, digital health experts, EIP service clinicians and service users. A private app development company based in the UK (MyOxygen; https://myoxygen.uk) led the technical development of My Journey 3. To limit costs My Journey 3 is only compatible with Smartphones with Android operating systems at this stage of testing.

My Journey 3 features four key elements of self-management, an approach with demonstrated efficacy in improving social and clinical outcomes for people with psychosis.[12] Screenshots of the key components are displayed in figure 1. Users have the ability to create a relapse prevention plan, where there is the opportunity to identify and list triggers, early warning signs of relapse and personalised coping strategies to refer to as required and to create a plan to follow if experiencing a crisis. Via the 'My Recovery Plan' section users are able to set recovery goals, list actions they can do to encourage well-being, and set reminders on their Smartphone to encourage engagement in these activities. Users can also use a tracker to monitor and rate their symptoms and early warning signs over time. In the Symptom Tracker users are presented with seventeen different symptoms and behaviours and are asked to respond via a "Yes/No" format as

For peer review only

to whether they have recently experienced these. Users that respond with a “Yes” are then presented with a 10-point scale (4-point scale for the early warning sign tracker) to rate the severity or frequency of the associated symptoms, with advice on how to manage these symptoms displayed. Psycho-education on mental health, medication and mental health services is provided in an ‘Information’ section. To encourage adherence with medication, users are encouraged to log and track their medication in the ‘Pill Tracker’ section. Users are able to set daily alerts to remind them to log whether they have taken their medication. My Journey 3 also features weekly discrete notifications to encourage engagement with the app, which can be disabled at the users’ preference. The key components of My Journey 3 are summarized in Table 1, with further details available in the protocol paper.[32]

Prior to the feasibility trial reported in this paper, My Journey 3 was tested by EIP service users in lab-based usability tests and in a one-month field study. The final content of My Journey 3 was then refined based on feedback from individual interviews with the participating EIP service users and clinicians. No changes were made to the content of My Journey 3 during the feasibility RCT. A major technical update to My Journey 3 was carried out in January 2018 to fix compatibility issues with older versions of Android operating systems. This did not require any changes to the trial design.

For peer review only

Table 1. Key sections of the My Journey 3 Smartphone app.

Section	Features	Purpose
My recovery plan	Things I can do to keep well<br><br>My goals	To encourage users to have regular routines, track activities, set reminders and plan how to achieve long-term goals
My relapse prevention plan	Coping with triggers<br><br>Coping with early warning signs<br><br>Coping with a crisis<br><br>Crisis contacts	To help users' identify, monitor and cope with triggers and early warning signs<br><br>To help users create a "relapse plan" to follow in times of crisis
How are you doing?	My mood<br><br>My early warning signs<br><br>My tracker	For users to monitor symptoms, behaviours and early warning signs and track these experiences over time
Pill tracker		To log whether users' have taken their medication each day
Information	Medication information<br><br>Useful websites<br><br>Emergency services<br><br>Jargon buster	To provide users with useful information and external links on medication and mental health<br><br>To identify local emergency services in a time of crisis<br><br>To provide a glossary of terms that are commonly used in mental health care

Delivery

Following assignment to the treatment group, participants engaged in individual training sessions with a trial researcher and a supporting EIP service clinician. Training sessions were intended to take place within six weeks of the participants' initial baseline assessment, and lasted

For peer review only

for approximately 2 hours. During these sessions the researcher downloaded My Journey 3 onto the participants' Smartphone and gave a demonstration of the app and its main functions. Participants were then encouraged to input appropriate information to specific sections of My Journey 3 with the help of the supporting EIP service clinician in attendance. Following this session it was hoped that all participants had initial personal recovery plans, relapse prevention plans and crisis plans stored on My Journey 3.

Participants had access to My Journey 3 on their own Smartphone from the training session till the 12-month time-point. Researchers recommended that participants used My Journey 3 at least once a week, but participants had a free choice in how and when they used My Journey 3. Participants did not receive any financial incentives to use My Journey 3, and were free to withdraw from using the app or decline the installation of it on to their Smartphone. At the training session participants were informed by the researcher that My Journey 3 would be not suitable for seeking urgent medical care whilst in crisis, and that it is not a substitute for human support.

To encourage user engagement with My Journey 3 during the trial, supporting EIP service clinicians were asked to provide regular support and encouragement to service users who had access to My Journey 3. Clinicians were asked to discuss recovery goals and relapse prevention plans in routine appointments with participants, and assist with entering these into the appropriate My Journey 3 sections. Clinicians had an existing understanding of self-management approaches from their clinical training and practice, and would be able to provide appropriate advice with the intervention components of My Journey 3 but they received no formal training on how to implement My Journey 3 into their clinical work. Clinicians' understanding of operating My Journey 3 was from the training sessions only. Clinician support for My Journey 3 as part of the trial was not manualised or incentivised.

For peer review only

Participants were encouraged to contact the trial researcher in the case of technical problems with My Journey 3. The researcher contacted participants a week after the training session to check that My Journey 3 had been functioning without issues, and invited any questions about the app. No further prompts were instigated by the researcher during the trial.

Treatment as usual

All participants received TAU regardless of group allocation. TAU for a person under the care of EIP services typically involves regular meetings with a care co-ordinator, access to a psychiatrist, psychiatric medication, and a range of psychological interventions. EIP services are encouraged to deliver self-management programmes, that includes advice on symptom management, crisis planning and relapse prevention, generally delivered with paper-and-pen tools if at all.[34] None of the participating EIP services offered digital interventions or Smartphone apps as part of routine care during the study period, and structured self-management support, including the relapse prevention work recommended in EIP contexts, is inconsistently implemented.

Patient and Participant Involvement

The development of My Journey 3 has been guided by the input of people with lived experience of psychosis. Initial development of the design and content involved a collaboration between researchers, experts in digital health and service users. Service users provided further input into the design and functionality of My Journey 3 from providing feedback after taking part in lab-based tests and a field study.

For peer review only

Outcomes

Participant data were collected from numerous sources including participant assessments, patient records and anonymous My Journey 3 usage reports. There were no pre-specified criteria for assessing trial feasibility and intervention acceptability.

Questionnaire measures

Proposed outcome measures for a future trial were assessed at structured face-to-face assessments with a trained researcher at three time points; baseline, 4-months post baseline and 12-months post baseline. At all meetings participants completed self-report questionnaires that have been previously used with people with first-episode psychosis. Participants were given £20 as a thank you for completing the assessment at each time point.

At each assessment we collected sociodemographic data including age, gender, ethnicity, accommodation and living situation, employment status, educational attainment, Smartphone use, and use of other mental health apps. The following self-report measures were also collected: social outcomes (Social Outcomes Index (SIX),[38] score 0-6: higher score= better social outcomes), self-efficacy (Mental Health Confidence Scale (MHCS),[39] score 16-96: higher score= greater empowerment), self-rated recovery (Questionnaire about the Process of Recovery (QPR),[40] intrapersonal score 0-68, interpersonal score 0-20: higher score= greater recovery), mental well-being (Warwick-Edinburgh Mental Well-Being Scale (WEMWBS),[41] score 14-70: higher score= greater well-being) and quality of life and satisfaction with treatment (DIALOG scale,[42] score 1-7: higher score= greater quality of life/satisfaction with treatment).

For peer review only

Clinical structured interviews were also conducted with each participant by the researcher, to assess psychopathology, using the Positive and Negative Syndrome Scale (PANSS).[43] Higher PANSS scores are indicative of greater severity of each symptom domain.

Participants' engagement with EIP services were measured using the Service Engagement Scale (SES),[44] completed by EIP service clinicians known to each participant, typically care co-coordinators. Clinicians completed the SES at baseline and 12 months later, regardless of whether participants attended the 12-month assessment. Higher SES scores are indicative of poorer user engagement with EIP services.

Patient records

Clinical data were extracted from patient records at baseline and at the 12-month time point. Clinical measures included most recent diagnosis and use of EIP services, other community mental health teams and acute mental health services in the previous 12 months.

The proposed primary outcome for a future RCT (relapse of psychosis) was operationalised as an admission to an acute mental health service (inpatient psychiatric ward, crisis house, crisis resolution team or acute day care service) during the 12-month trial period as indicated in patient records. This definition of relapse has been used previously in a recent trial of a self-management intervention.[45]

My Journey 3 use

To assess acceptability of the intervention and user engagement, My Journey 3 usage data were collected for all participants in the treatment group from the training session until the 12-month time-point. Whenever users had Wi-Fi internet access on their Smartphone My Journey 3 automatically uploaded encrypted usage data to a secure server. Data collected included a record

For peer review only

of each time the user opened My Journey 3, whether this was in response to a prompt, and which components they used. To ensure confidentiality, personal or identifiable data such as text or responses to each sections were not collected.

Analysis

Participant demographic and clinical characteristics, My Journey 3 usage, and rates of participant recruitment and retention were summarised using descriptive statistics. As this was a feasibility RCT, it was not powered to assess the effectiveness of the intervention. Statistical analyses of participant outcome measures were conducted to pilot the methods of analysis for a fully powered effectiveness trial. Logistic regression was used to explore the impact of the My Journey 3 intervention on relapse. Linear regression was used to examine the potential effect of the intervention on continuous outcome measures at 4 months and 12 months separately. We report the effect estimates and corresponding 95% confidence intervals (CI) only for unadjusted analyses and for analyses adjusting for the baseline measure of the outcome in question. All analyses were performed using STATA V.14 after completion of the final participant assessment. No interim analyses were conducted.

RESULTS

Feasibility of trial design

Participant flow is detailed in the CONSORT diagram (figure 2). A total of 40 participants was recruited and randomised (20 to My Journey 3, 20 to TAU) over a 7-month period from March 2017 to September 2017. Participants were recruited until the required number of 40 was obtained: we do not therefore have a full assessment of the proportion of the teams' caseload who could have

For peer review only

been recruited to a full trail, nor do we know the proportion of approached EIP services users that did not meet eligibility criteria or declined involvement in the trial.

Among those recruited to the trial, attrition rates were generally low: 83% (33/40) and 75% (30/40) of participants successfully attended and completed follow-ups at 4 months and 12 months respectively. At both time points the follow-up rate was lower in the control group (4-months: 65% compared to 100%, 12-months: 70% compared to 80%). Patient record data were available for all participants at baseline and for 95% of the sample (38/40) at the 12-month time-point. Completion rates of the SES by clinicians were higher at baseline (90%) than at the 12-month time-point (67.5%). Follow-up assessments were conducted from July 2017 to October 2018.

All participants in the treatment group attended a training session with a researcher, and had access to My Journey 3 during the trial. Issues with Smartphone compatibility initially prevented three participants from downloading My Journey 3. Following an update to the system two of the participants were able to install and access My Journey 3 on their own Smartphones. Two participants were provided with Smartphones with My Journey 3 pre-installed (the app was still incompatible on one participant's Smartphone despite the update; another participant no longer owned an Android Smartphone after entering the trial). The median length of time from trial enrolment to having access to My Journey 3 was 14 weeks (IQR 11 to 17), longer than the planned time of 6 weeks. Participants had access to My Journey 3 for a median of 38.1 weeks (IQR 34.8 to 40.7). There were no reported privacy breaches.

My Journey 3 usage data were collected for all participants following the training session, with 500 different data entries available for analysis. Within the 500 data entries, 27 (5.4%) were corrupt and were subsequently removed from the analysis. The unusable data can grouped into two types. The first, duplicates of previous data entries that were subsequently removed. The

For peer review only

second, entries where the times were implausible (for example, the end time of using My Journey 3 was recorded as occurring before the start time). In addition, a further issue caused errors with accurately recording My Journey 3 usage data of 'My Recovery Plan' and 'My Relapse Plan' sections. As a result we were unable to accurately conclude how often participants used these sections.

One participant randomised to the control group was wrongly given access to My Journey 3. For the purpose of the statistical analysis they are classed as a control participant.

Sample characteristics

A summary of demographic and clinical characteristics of the sample is displayed in Table 2. The sample was predominantly male (n=28, 70%). Most participants had a diagnosis of a schizophrenia, schizotypal or delusional disorder (ICD code F20-F29) and were not in paid employment. A quarter of the sample (n=10, 25%) had completed a university degree. Eight (20%) participants had previously used a mental health app.

Table 2. Key demographic and clinical characteristics of the sample at baseline.

	Control (n=20)	My Journey 3 (n=20)
**Age (years) – mean (SD), [min, max]**	30 (10.1), [18.8, 64.7]	29.4 (9.7), [17.6, 52.4]
**Gender**		
Female	7 (35%)	5 (25%)
**Ethnicity**		
White British	6 (30%)	8 (40%)
Any other white/Mixed white	2 (10%)	1 (5%)
Black African	5 (25%)	3 (15%)
Black Caribbean	1 (5%)	1 (5%)
Black Other	1 (5%)	0
Asian Indian	1 (5%)	0
Asian Other	1 (5%)	2 (10%)
Other/Mixed other	3 (15%)	3 (15%)
**Education**		
Undergraduate degree	6 (30%)	4 (20%)
Some University but no degree	3 (15%)	2 (10%)
Higher National Degree or professional qualification	2 (10%)	1 (5%)
A Levels or equivalent	3 (15%)	4 (20%)

For peer review only

GCSEs or equivalent	4 (20%)	6 (30%)
No qualifications	1 (5%)	3 (15%)
Missing	1 (5%)	0
**Employment status**		
Employed – more than 16 hours a week	4 (20%)	4 (20%)
Employed – less than 16 hours a week	0	2 (10%)
Voluntary work	3 (15%)	3 (15%)
In study or training	1 (5%)	1 (5%)
Unemployed or exempt due to disability	8 (40%)	8 (40%)
Missing	4 (20%)	2 (10%)
**Primary diagnosis (ICD-10 code)**		
F10-F19: Mental and behavioural disorder due to psychoactive substance use	1 (5%)	0
F20-F29: Schizophrenia, schizotypal and delusional disorder	16 (80%)	13 (65%)
F30-F39: Mood disorder	1 (5%)	5 (25%)
Missing	2 (10%)	2 (10%)
**Admission to an acute mental health service in previous year**		
Yes	11 (55%)	10 (50%)
**SIX – mean (SD), [min, max]**	3.2 (1.5), [0, 6]	3.6 (1.5), [1, 6]
**MHCS – mean (SD), [min, max]**	59.7 (17.8), [16, 82]	61.2 (12.6), [38, 78]
**QPR – mean (SD), [min, max]**		
Intrapersonal	45.7 (12), [22, 68]	42.2 (10.6), [24, 60]
Interpersonal	13.7 (2.7), [9, 19]	12.9 (3.4), [5, 19]
**WEMWBS – mean (SD), [min, max]**	43.4 (11.6), [25, 69]	40.3 (10.2), [23, 57]
**DIALOG – mean (SD), [min, max]**		
Quality of life	4.5 (1), [2.8, 6.5]	4.4 (0.8), [3, 5.7]
Treatment satisfaction	5.4 (0.7), [4.3, 7]	4.8 (0.7), [3.7, 6]
**PANSS – mean (SD), [min, max]**		
Positive	10.9 (5), [7, 22]	11.3 (4.2), [7, 19]
Negative	10.7 (2.5), [7, 19]	11.8 (4.5), [7, 20]
General	26.6 (6), [17, 39]	26.2 (8), [16, 46]
**SES – mean (SD), [min, max]**	11.3 (7.9), [0, 26]	9.6 (7), [0, 23]

All statistics are reported N (%) unless otherwise specified. Missing data: PANSS scores – one control group participant, SES – three control group participants, one treatment group participant.

My Journey 3 use

The level of My Journey 3 use was highly skewed. The median number of times My Journey 3 was used per participant during the trial was 16.5 (IQR 8.5 to 23). Participants accessed My Journey 3 a median of 3.22% (IQR 1.89 to 6.36) of the days it was available to them, equating to My Journey 3 being used on average once every 31 days (IQR 15.7 to 52.9). Participants spent

For peer review only

a median of 26.8 minutes (IQR 18.3 to 57.3) in total using My Journey 3 over the course of the trial. Eight participants (40%) used My Journey 3 for longer than 30 minutes in total.

Five participants (25%) were still using My Journey 3 six months after downloading it, however one participant never used the app after the training session (figure 3). Half of the participants (n=10) stopped using My Journey 3 within the first three months after the training session.

The average number of uses by participants for each My Journey 3 component is displayed in table 3. The most frequently accessed section was the "How are you doing?" Symptom Tracker section (median uses 3; IQR 1 to 6), however data on how frequently users accessed 'My Recovery Plan' and 'My Relapse Plan' is unavailable. The 'Information' section was accessed the fewest times, with 25% (n=5) of participants in the treatment group never using that section following the training session. Just over 7% of My Journey 3 uses were initiated following a prompt from the app.

Table 3. Participant use of My Journey 3 and various sections.

	Number of times used per participant	Days used whilst having access to My Journey 3 (%)	Participants that did not use app or section – n (%)
My Journey 3	16.5 (8.5 to 23)	3.22 (1.89 to 6.36)	1 (5%)
How are you doing?	3 (1 to 6)	1.08 (0.4 to 2.12)	3 (15%)
Pill tracker	2 (1 to 3.5)	0.73 (0.36 to 1.07)	3 (15%)
Information	1 (0 to 2.5)	0.48 (0.18 to 0.7)	5 (25%)

All median (IQR), except when stated

For peer review only

Participant outcomes

No research-related serious adverse events were recorded. Psychotic and general symptoms (measured by the PANSS) were generally low at all times for both groups suggesting a stable sample. Summary statistics and estimated effect sizes of participant outcomes are displayed in table 4. Inspection of the effect sizes and confidence intervals suggest that were no obvious differences for any outcome measure between the treatment and control group at either time-point.

Of the 38 participants whose patient records data were available, only five experienced a relapse during the trial, as indicated by using an acute mental health service. In the treatment group 15% of participants (3/20) experienced a relapse during the trial period compared with 11% (2/18) in the control group. We found no evidence of a difference in relapse between the two groups (odds ratio: 1.41; 95% CI: 0.21 to 9.58), but did not have sufficient power for an informative test.

Table 4. Summary statistics and unadjusted and adjusted treatment effects.

	Control (N = 13)	My Journey 3 (N = 20)	Unadjusted analysis		Analysis adjusted for baseline score	
**4-month scores**	Mean (SD)	Mean (SD)	Estimated difference	95% CI	Estimated difference	95% CI
**SIX** (Social Outcomes)	3.3 (1.9)	3.6 (1.3)	0.29	-0.84 to 1.43	0.16	-0.6 to 0.92
**MHCS** (Mental Health Confidence)	66.4 (12.7)	63 (15.8)	-3.43	-14.1 to 7.25	-4.81	-14.88 to 5.25
**QPR** (Recovery)						
Intrapersonal	47.8 (10.6)	43.2 (12.2)	-4.57	-13 to 3.87	-2.01	-8.43 to 4.49
Interpersonal	13.9 (2.4)	13.2 (2.3)	-0.72	-2.39 to 0.95	-0.42	-1.97 to 1.13
**MHCS** (Mental Health Confidence)	46.1 (9.9)	44 (11.3)	-2.08	-9.9 to 5.74	-0.19	-7.28 to 6.9
**DIALOG**						
Quality of life	4.4 (1.2)	4.5 (0.6)	0.07	-0.58 to 0.71	0.18	-0.38 to 0.74
Treatment satisfaction	5.4 (0.7)	5 (0.5)	-0.38	-0.83 to 0.06	-0.17	-0.6 to 0.25
**PANSS** (Symptom severity)						
**Positive**	9.3 (2.9)	11.4 (5.1)	2.09	-1.24 to 5.4	1.9	-0.49 to 4.3
**Negative**	10 (2.3)	11.1 (3.9)	1.05	-1.51 to 3.62	0.54	-1.6 to 2.67
**General**	23 (4)	24 (6.7)	1.21	-3.19 to 5.61	1.35	-2.68 to 5.37
**12-month scores**	Control (N = 14)	My Journey 3 (N = 16)	Unadjusted analysis		Analysis adjusted for baseline score	

	Mean (SD)	Mean (SD)	Estimated difference	95% CI	Estimated difference	95% CI
**SIX** (Social Outcomes)	3.2 (1.9)	3.5 (1.5)	0.29	-0.97 to 1.54	0.29	-0.73 to 1.3
**MHCS** (Mental Health Confidence)	66.2 (14.1)	71.1 (12.1)	4.81	-5 to 14.62	3.03	-6.04 to 12.1
**QPR** (Recovery)						
Intrapersonal	47.3 (11.5)	49.5 (11.1)	2.2	-6.25 to 10.7	3.21	-4.12 to 10.5
Interpersonal	13.6 (3.4)	15.1 (3.3)	1.44	-1.09 to 3.96	1.62	-0.89 to 4.12
**MHCS** (Mental Health Confidence)	45.6 (11.3)	49.3 (9.7)	3.61	-4.24 to 11.46	5.03	-1.67 to 11.7
**DIALOG**						
Quality of life	4.7 (0.9)	5 (0.7)	0.28	-0.31 to 0.87	0.24	-0.33 to 0.81
Treatment satisfaction	5.3 (1)	5.2 (1.2)	-0.12	-0.93 to 0.69	0.31	-0.42 to 1.04
**PANSS** (Symptom severity)						
Positive	9.5 (2.1)	10.2 (2.1)	0.69	-0.98 to 2.36	0.88	-0.62 to 2.38
Negative	10.2 (2.2)	10.9 (3.3)	0.77	-1.51 to 3.05	0.14	-1.56 to 1.84
General	23.5 (5.4)	22.1 (3.5)	-1.38	-4.82 to 2.07	-1	-4.57 to 2.55
**SES** (Engagement with services)	10 (6.2)	9.5 (8)	-0.4	-6.08 to 5.28	3.11	-1.57 to 7.79

Estimated differences and associated 95% Confidence Intervals from linear regression models with the control group as reference. Missing data: 4-month PANSS scores – one control group participant, one treatment group participant. 12-month PANSS scores – two control group participants. Note: 12-month SES data available for 13 control group participants, and 14 treatment group participants.

DISCUSSION

The present study examined the feasibility of conducting a RCT of a supported self-management Smartphone app in EIP services. My Journey 3 aims to facilitate recovery and prevent relapse primarily via the digital delivery of previously developed paper-and-pen self-management tools. The trial indicates that recruitment and retention in a RCT evaluating My Journey 3 is feasible, and that My Journey 3 can be delivered in EIP services. The level of My Journey 3 use was relatively low across the trial period.

Building on from extensive preliminary work with NHS staff and service users, adults with lived experience of psychosis and experts in digital health we were able to successfully develop a

For peer review only

self-management Smartphone app that can be used in EIP services. My Journey 3 appeared to be safe with no related serious adverse events reported. My Journey 3 was successfully delivered to all participants in the treatment group, however technical problems with the intervention caused significant delays in providing access. Prior to any future evaluations technical problems with My Journey 3 will need to be identified and fixed to ensure the intervention is implemented as intended.

My Journey 3 use varied considerably between participants, with only a small proportion of participants frequently engaging with the app after obtaining access to it. This raises questions about whether use was at a level where it is likely that useful self-management activities were taking place: certainly not enough time was spent regularly enough for participants to be engaging in detailed monitoring of symptoms and early warning signs, tracking medication and activities and referring to crisis or recovery plans. Despite that, 40% of participants used My Journey 3 for a minimum of 30 minutes which could be an adequate amount of time for users to effectively monitor relapse signs and follow a crisis plan when needed. We have not found evidence on how regularly EIP service users make use of pen and paper self-management interventions delivered in routine settings, and this was not measured in our trial. Long-term engagement with My Journey 3 appears a challenge, but low levels of app use is a common phenomenon with market research showing that 62% of users stop using Smartphone apps after ten or fewer uses.[46] We will report separately on qualitative findings from this study exploring further the acceptability of My Journey 3 and drivers of engagement and non-adherence.

Participant retention for research data collection was high, with 75% of the sample attending the 12-month follow-up assessment, and is comparable to other Smartphone app studies.[47] Completion rates of the SES by EIP service clinicians were much lower at the 12-

For peer review only

month follow in comparison to baseline, potentially due to staff changes and participants being discharged from services. Recruitment strategies were largely successful, however data is lacking on overall proportion of caseload recruited, reasons for non-inclusion and the numbers that were assessed for eligibility, thus limiting the conclusions we can make regarding trial feasibility.

The trial was not powered to detect effectiveness, and, as expected with our small number of participants, we found no significant differences between groups on any outcomes, with confidence intervals generally including substantial effects in either direction. Accordingly we cannot draw any conclusions regarding the potential impact of My Journey 3 as a mental health intervention. The proposed primary outcome for a full-scale trial, relapse as defined by use of an acute mental health service during the trial period, was marked by low event rates. Only five participants (12.5%) experienced a relapse during the one year follow-up period, compared with expected levels of 12% to 47%.[48] Consideration should be given to whether relapse, or our measure of relapse, is an appropriate outcome for a future RCT of this intervention. Symptom severity or alternatively patient-valued outcomes of personal recovery that self-management interventions have been shown to benefit may be more suitable primary outcomes in a future large scale trial.[12]

For peer review only

Strengths and limitations

My Journey 3 has been developed with extensive stakeholder input, and the intervention has been tested through lab-testing and a field study prior to the feasibility RCT. In comparison to previous studies,[47] participants had access to the app for a longer period of time. Participants' app use and usage data may be more reflective of real-world use as a result. Participant data were also collected from a wide range of methods including from participant assessments and patient

records. The proposed primary outcome for a future RCT (relapse) was measured objectively and data were obtained for 95% of participants.

We recruited until the required number of participants was obtained rather than screening caseloads objectively: as a result we are not aware of the proportion eligible who were recruited, reasons for non-eligibility and how many EIP service users declined to take part and why. This limits our understanding of how feasible conducting a large scale trial of this intervention would be. In addition there were issues with the usage data, which impacts the reliability of our conclusions regarding how often participants engaged with My Journey 3.

The trial did not feature an active digital placebo for the control group, meaning that non-specifics of Smartphone use could not be controlled for. Furthermore data was not collected during the study period from either group regarding frequency of completing recovery work such as relapse prevention plans, recovery plans or crisis plans either in paper-and-pen or digital format, limiting our understanding of whether access to My Journey 3 facilitated increased access to self-management activities.

Although clinicians were encouraged to support participants with My Journey 3, support was not manualised and clinicians did not have personal access to the app or associated data, potentially limiting the level and quality of the support offered and therefore user engagement. Future developments of My Journey 3 should focus on effective implementation and delivery within healthcare settings, and there should be considerations on how to facilitate secure data-sharing between My Journey 3 and healthcare records or other secure web-based platforms dependent on user consent, which is likely to increase clinician engagement with the app and its utility.[49]

For peer review only

We did also not define pre-specified criteria for assessing the feasibility of a RCT and the acceptability of My Journey 3. Instead we will consider all findings from the trial, app usage data and feedback from qualitative interviews yet to be reported in determining whether My Journey 3 will be evaluated in a full-scale trial. This allows all data from the RCT to be thoroughly considered, but may be a less objective approach in determining feasibility than using pre-defined criteria. Although the trial was not designed to assess intervention effectiveness, participants and trial researchers were not blinded to group allocation, and as such could have led to an inflation of any observed effects.

Finally the sample consisted of Android Smartphone users who were generally stable and in an appropriate stage of recovery to consider using a self-management Smartphone app. Participants may therefore not be representative of all EIP service users. Furthermore contact with a researcher within a trial context could have led to increased intervention engagement that would not occur in a real-world clinical environment.

Conclusions

We developed and delivered a self-management Smartphone app for first-episode psychosis in a trial context. Participants were successfully recruited, most engaged at least to some extent with the intervention, and they had high follow-up rates over the one year trial period. Based on the data presented the trial methods appear feasible. My Journey 3 was shown to be safe, but the level of use was lower than anticipated thus potentially limiting its utility, although usage levels were higher than reported for downloaded apps in the general population.

If My Journey 3 is to be further tested in a research setting, attention needs to be given to engagement, a challenge associated with many digital tools in mental health.[50] Further usability

For peer review only

testing in lab and field settings may also be a means to improving engagement. Other potential strategies including making more efforts to engage clinicians as well as service users with My Journey 3 by giving them access to the tool and to aspects of the planning and monitoring that service users conduct through it. The app could also potentially be offered as part of a blended approach to self-management, with pen and paper tools also used and as a whole service strategy for implementation of self-management. Refinements required before participating to a full trial including participant and assessor blinding and manualised clinician support should be considered prior to conducting a future RCT.

For peer review only

Acknowledgements: The ARIES research team are grateful to their software collaborators MyOxygen for their technical development and hosting of My Journey 3 and to Ali Mousa for his valuable contribution to the development of the original My Journey app. We are grateful to Max Birchwood for his permission to incorporate 'Back in the Saddle' in to My Journey 3. We are grateful to Rachel Perkins for her permission to adapt the Personal Recovery Plan resource and incorporate in to My Journey 3.

Author Contributions: SJ is the Chief Investigator, based at University College London, DO the co-Chief Investigator, and TS the project manager. The trial design was developed by SJ, DO, BLE and PO. SA, HR, PO and ME have led on the development of the intervention. TS conducted the statistical analysis, with advice from RJ. TS wrote the draft of the paper, which was revised and approved by all authors. All authors approved the final manuscript.

Funding: The research is funded by the National Institute for Health Research (NIHR) Collaboration for Leadership in Applied Health Research and Care North Thames at Barts Health NHS Trust (NIHR CLAHRC North Thames). The views expressed in this article are those of the authors and not necessarily those of the NHS, the NIHR or the Department of Health and Social Care. SJ, DO and BLE are supported by the NIHR Mental Health Research Policy Unit, the NIHR Collaboration for Leadership in Applied Health Research and Care (CLAHRC) North Thames and the UCLH Biomedical Research Centre.

Data statement: The datasets generated during and/or analysed during the current study will be made available two years after the trial end.

Competing interests: None declared.

For peer review only

Legends:

Figure 1. Screenshots of the My Journey 3 app. a) The homescreen, b) the ‘My goals’ section of the recovery plan, c) the ‘Coping with early warning signs’ section of the relapse prevention plan, d) an example item from the Symptom Tracker, e) the Information section.

Figure 2. CONSORT diagram of the ARIES feasibility trial. Note: DNA, did not attend.

Figure 3. Bar chart displaying how long after the training session participants disengaged with My Journey 3.

For peer review only

REFERENCES

1 Bird V, Premkumar P, Kendall T, et al. Early intervention services, cognitive–behavioural therapy and family intervention in early psychosis: systematic review. *Brit J Psychiat*. 2010;197:350-6.

2 Tsiachristas A, Thomas T, Leal J, et al. Economic impact of early intervention in psychosis services: results from a longitudinal retrospective controlled study in England. *BMJ Open* 2016;6:e012611.

3 Alvarez-Jiménez M, Parker AG, Hetrick SE, et al. Preventing the second episode: a systematic review and meta-analysis of psychosocial and pharmacological trials in first-episode psychosis. *Schizophrenia Bull*. 2009;37:619-30.

4 Kam SM, Singh SP, Upthegrove R. What needs to follow early intervention? Predictors of relapse and functional recovery following first-episode psychosis. *Early Interv Psychia*. 2015;9:279-83.

5 Puntis S, Oke J, Lennox B. Discharge pathways and relapse following treatment from early intervention in psychosis services. *BJPsych open* 2018;4:368-74.

6 Leucht S, Heres S. Epidemiology, clinical consequences, and psychosocial treatment of nonadherence in schizophrenia. *J Clin Psychiatry* 2006;67:3-8.

7 Haddock G, Eisner E, Boone C, et al. An investigation of the implementation of NICE-recommended CBT interventions for people with schizophrenia. *J Ment Health* 2014:1;23:162-5.

8 Mueser KT, Meyer PS, Penn DL, et al. The Illness Management and Recovery program: rationale, development, and preliminary findings. *Schizophrenia Bull* 2006;32:S32-43.

For peer review only

9 Mueser KT, Corrigan PW, Hilton DW et al. Illness management and recovery: A review of the research. *Psychiat Serv* 2002;53:1272-84.

10 Cook JA, Copeland ME, Jonikas JA, et al. Results of a randomized controlled trial of mental illness self-management using Wellness Recovery Action Planning. *Schizophrenia Bull* 2011;38:881-91.

11 Färdig R, Lewander T, Melin L, et al. A randomized controlled trial of the illness management and recovery program for persons with schizophrenia. *Psychiat Serv* 2011;62:606-12.

12 Lean M, Fornells-Ambrojo M, Milton A, et al. Self-management interventions for people with severe mental illness: systematic review and meta-analysis. *Brit J Psychiat* 2019;22:1-9.

13 NICE Guidance - Psychosis and schizophrenia in adults: prevention and management. https://www.nice.org.uk/guidance/cg178/ifp/chapter/peer-support-and-self-management [accessed 2019-01-30]

14 Whitehead L, Seaton P. The effectiveness of self-management mobile phone and tablet apps in long-term condition management: a systematic review. *J Med Internet Res* 2016;18:e97.

15 Poushter J. 2016 Smartphone ownership and internet usage continues to climb in emerging economies. URL: http://www.pewglobal.org/2016/02/22/smartphone-ownership-and-internet-usage-continues-to-climb-in-emerging-economies/ [accessed 2019-01-30]

16 Wigginton C, Curran M, Broedeur C. 2017. Global mobile consumer trends, 2nd edition. URL: https://www2.deloitte.com/content/dam/Deloitte/us/Documents/technology-media-telecommunications/us-global-mobile-consumer-survey-second-edition.pdf. [accessed 2019-07-04]

For peer review only

17 Firth J, Cotter J, Torous J, et al. Mobile phone ownership and endorsement of "mHealth" among people with psychosis: a meta-analysis of cross-sectional studies. *Schizophrenia Bull* 2015;42:448-55.

18 Robotham D, Satkunanathan S, Doughty L, Wykes T. Do We Still Have a Digital Divide in Mental Health? A Five-Year Survey Follow-up. *J Med Internet Res* 2016;18:e309.

19 Naslund JA, Aschbrenner KA, Bartels SJ. How people with serious mental illness use smartphones, mobile apps, and social media. *Psychiatr Rehabil J* 2016;39:364-367.

20 Aref-Adib G, O'Hanlon P, Fullarton K, et al. A qualitative study of online mental health information seeking behaviour by those with psychosis. *BMC Psychiatry* 2016;16:232.

21 Berry N, Lobban F, Bucci S. A qualitative exploration of service user views about using digital health interventions for self-management in severe mental health problems. *BMC Psychiatry* 2019;19:35.

22 Proudfoot J. The future is in our hands: the role of mobile phones in the prevention and management of mental disorders. *Aust NZ J Psychiat* 2013;47:111-3.

23 Gottlieb JD, Romeo KH, Penn DL, et al. Web-based cognitive–behavioral therapy for auditory hallucinations in persons with psychosis: a pilot study. *Schizophr Res* 2013;145:82–87.

24 Granholm E, Ben-Zeev D, Link PC, et al. Mobile Assessment and Treatment for Schizophrenia (MATS): a pilot trial of an interactive text-messaging intervention for medication adherence, socialization, and auditory hallucinations. *Schizophr Bull* 2011;38:414-25.

25 Ben-Zeev D, Kaiser SM, Brenner CJ, et al. Development and usability testing of FOCUS: A smartphone system for self-management of schizophrenia. *Psychiatr Rehabil J* 2013;36:289-296.

For peer review only

26 Alvarez-Jimenez M, Bendall S, Lederman R, et al. On the HORYZON: moderated online social therapy for long-term recovery in first episode psychosis. *Schizophr Res* 2013;143:143-9.

27 Bonet L, Izquierdo C, Escartí MJ, et al. Use of mobile technologies in patients with psychosis: A systematic review. *Rev Psiquiatr Salud Ment* 2017;10:168-78.

28 Rus-Calafell M, Schneider S. Are we there yet?!—a literature review of recent digital technology advances for the treatment of early psychosis. *mHealth* 2019. doi: 10.21037/mhealth.2019.09.14

29 Camacho E, Levin L, Torous J. Smartphone Apps to Support Coordinated Specialty Care for Prodromal and Early Course Schizophrenia Disorders: Systematic Review. *J Med Internet Res* 2019;21:e16393.

30 Bucci S, Barrowclough C, Ainsworth J, et al. Actissist: Proof-of-Concept Trial of a Theory-Driven Digital Intervention for Psychosis. *Schizophrenia Bull* 2018; sby032.

31 JMIR. URL: https://support.jmir.org/hc/en-us/articles/115001389307-Does-my-trial-RCT-have-to-be-registered- [accessed 2019-10-08]

32 Steare T, O'Hanlon P, Eskinazi M, et al. App to support Recovery in Early Intervention Services (ARIES) study: protocol of a feasibility randomised controlled trial of a self-management Smartphone application for psychosis. *BMJ Open* 2019;9:e025823.

33 Eldridge SM, Chan CL, Campbell MJ, et al. CONSORT 2010 statement: extension to randomised pilot and feasibility trials. *BMJ* 2016;355:i5239.

34 National Institute for Health and Care Excellence. Implementing the Early Intervention in Psychosis Access and Waiting Time Standard: Guidance. London: NHS England Publications. 2016. p. 1-57.

For peer review only

35 Julious SA. Sample size of 12 per group rule of thumb for a pilot study. *Pharm Stat* 2005;287-91.

36 Plaistow J, Birchwood M. Back in the saddle: a guide to relapse prevention. In: Birchwood M, Fowler D, Jackson C, editors. Early Intervention in Psychosis: A Guide to Concepts, Evidence and Interventions. Chichester: McGraw-Hill; 1996. p. 239-244.

37 Perkins R, Rinaldi M. Taking back control: a guide to planning your own recovery. South West London: St George's Mental Health NHS Trust, 2007.

38 Priebe S, Watzke S, Hansson L, et al. Objective social outcomes index (SIX): a method to summarise objective indicators of social outcomes in mental health care. *Acta Psychiat Scand* 2008;118:57-63.

39 Carpinello SE, Knight EL, Markowitz FE, et al. The development of the Mental Health Confidence Scale: A measure of self-efficacy in individuals diagnosed with mental disorders. *Psychiatr Rehabil J* 2000;23:236-243.

40 Neil ST, Kilbride M, Pitt L, et al. The questionnaire about the process of recovery (QPR): a measurement tool developed in collaboration with service users. *Psychosis* 2009;1:145-55.

41 Tennant R, Hiller L, Fishwick R, et al. The Warwick-Edinburgh mental well-being scale (WEMWBS): development and UK validation. *Health Qual Life Out* 2007;5:63.

42 Priebe S, McCabe RO, Bullenkamp J, et al. Structured patient–clinician communication and 1-year outcome in community mental healthcare. *Brit J Psychiat* 2007;191:420-6.

43 Kay SR, Fiszbein A, Opfer LA. The positive and negative syndrome scale (PANSS) for schizophrenia. *Schizophrenia Bull* 1987;13:261-76.

For peer review only

44 Tait L, Birchwood M, Trower P. A new scale (SES) to measure engagement with community mental health services. *J Ment Health* 2002;11:191-8.

45 Johnson S, Lamb D, Marston L, et al. Peer-supported self-management for people discharged from a mental health crisis team: a randomised controlled trial. *Lancet* 2018;392:409-18.

46 Localytics. 21% of users abandon an app after one use. http://info.localytics.com/blog/21-percent-of-users-abandon-apps-after-one-use. [accessed 2019-05-16]

47 Firth J, Torous J. Smartphone apps for schizophrenia: a systematic review. *JMIR Mhealth Uhealth* 2015;3.

48 Alvarez-Jimenez M, Priede A, Hetrick SE, et al. Risk factors for relapse following treatment for first episode psychosis: a systematic review and meta-analysis of longitudinal studies. *Schizophr Res* 2012;139:116-28.

49 Torous J, Woodyatt J, Keshavan M, Tully LM. A new hope for early psychosis care: the evolving landscape of digital care tools. *British J Psychiat* 2019;214:269-72.

50 Aref-Adib G, McCloud T, Ross J, et al. Factors affecting implementation of digital health interventions for people with psychosis or bipolar disorder, and their family and friends: a systematic review. *Lancet Psychiat* 2018;6:257-66.

For peer review only

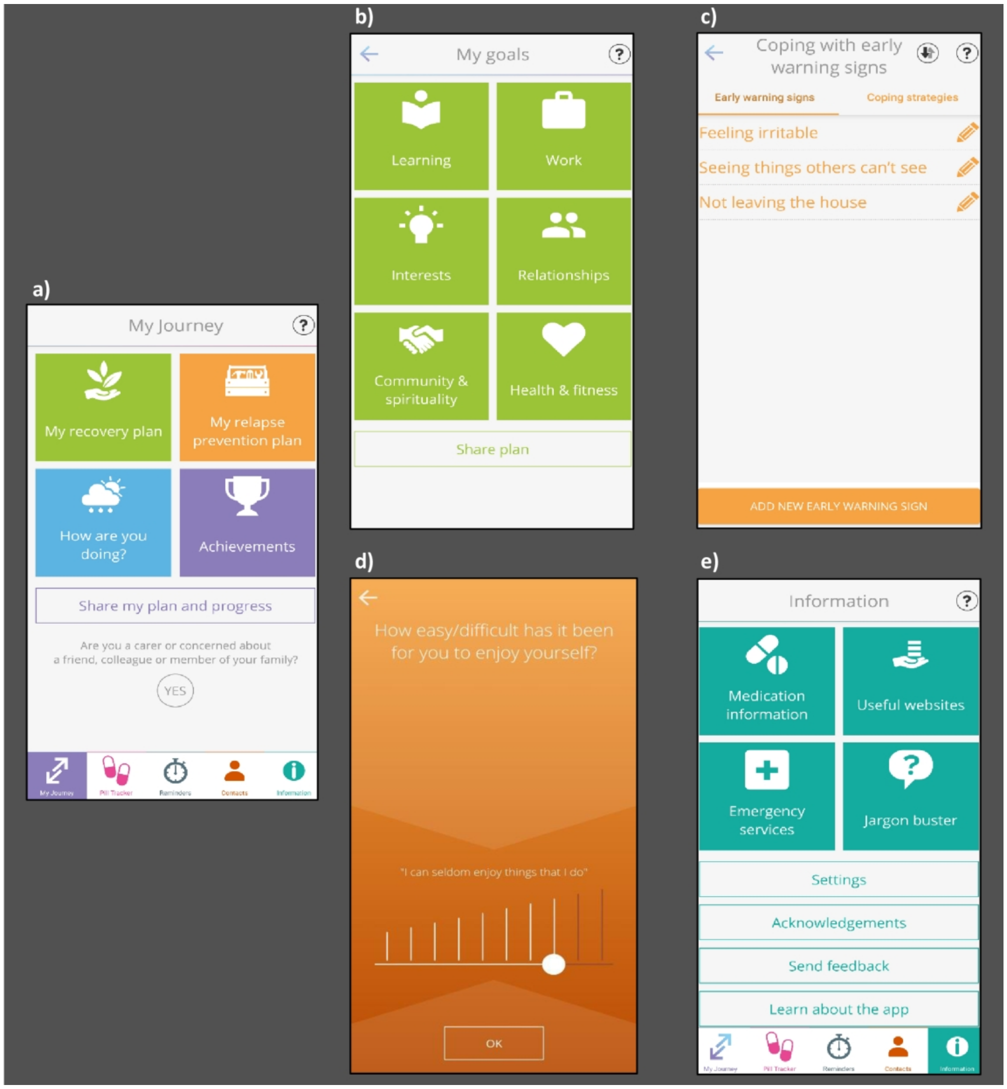

Figure 1. Screenshots of the My Journey 3 app. a) The homescreen, b) the 'My goals' section of the recovery plan, c) the 'Coping with early warning signs' section of the relapse prevention plan, d) an example item from the Symptom Tracker, e) the Information section.

169x182mm (300 x 300 DPI)

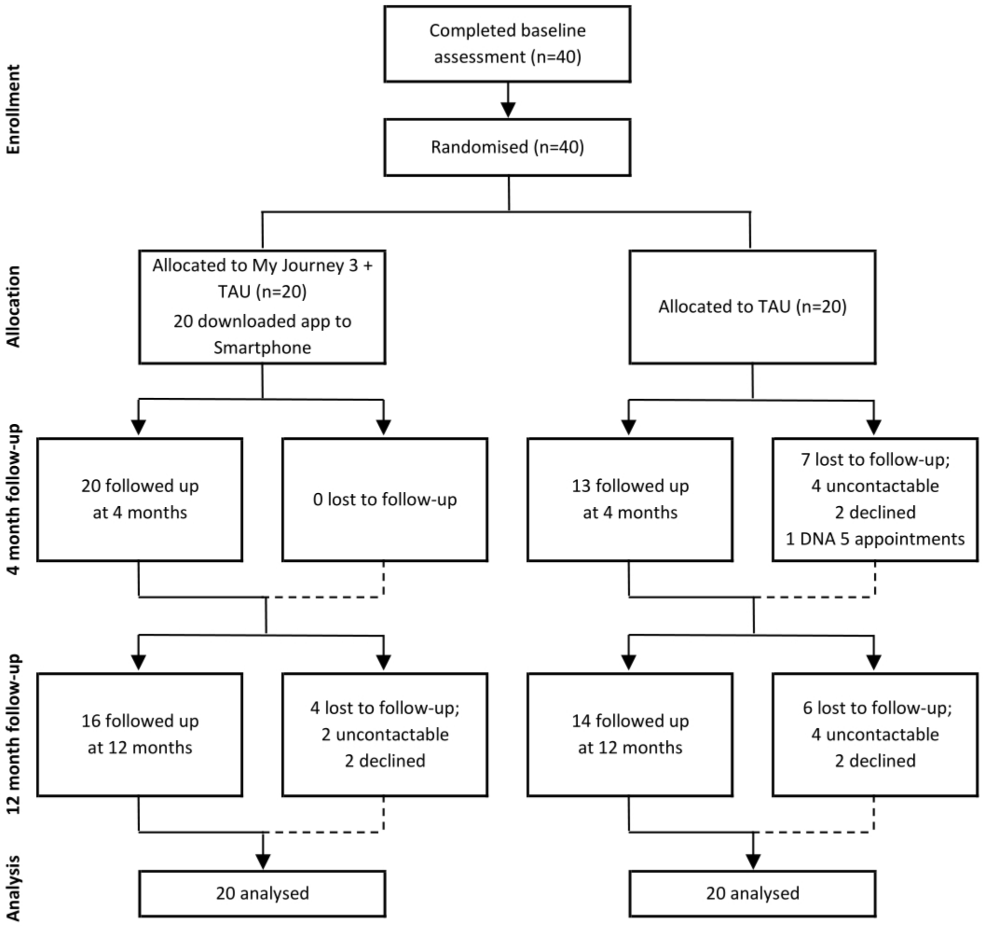

Figure 2. CONSORT diagram of the ARIES feasibility trial. Note: DNA, did not attend.

90x85mm (300 x 300 DPI)

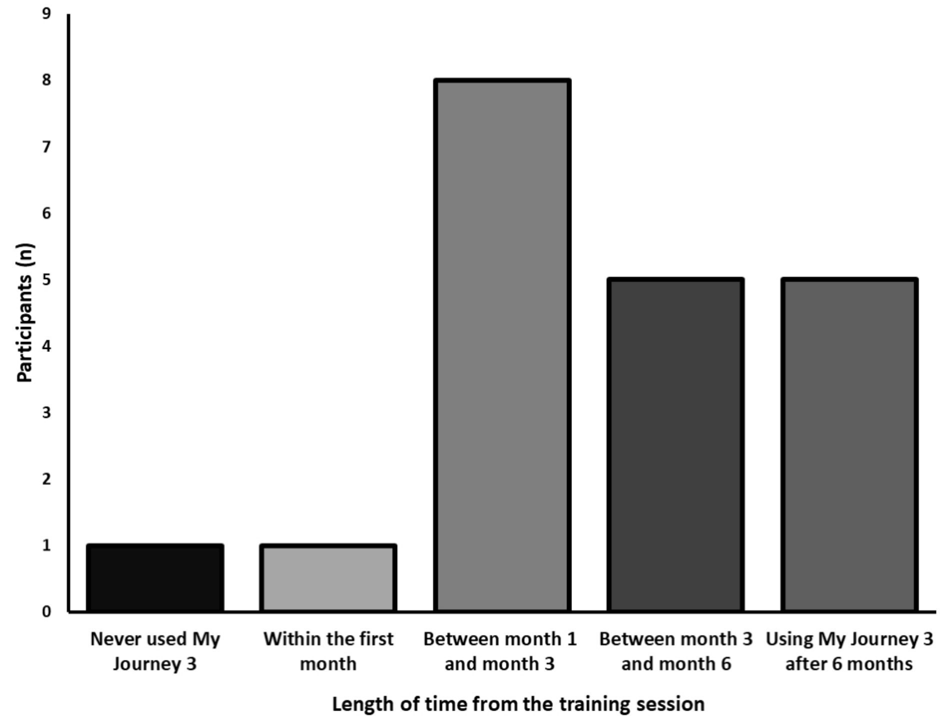

Figure 3. Bar chart displaying how long after the training session participants disengaged with My Journey 3.

101x79mm (300 x 300 DPI)

Service user participant consent form

Study Title: App to support Recovery In Early intervention Services (the ARIES study): Pilot randomised controlled trial of a self-management smartphone application

Principal Investigators: Professor Sonia Johnson and Professor David Osborn

	Please Initial Each Box
1. I confirm that I have read and understood the Participant Information Sheet V5 dated 29/05/2017 for the above study and have had the opportunity to ask questions about the study.	☐
2. I understand that my participation is voluntary and that I am free to withhold personal information or to withdraw my participation at any time, without giving any reason, and without my medical care or legal rights being affected.	☐
3. I understand that if I choose to withdraw from the study that any data that I have already provided for the purposes of the research will be kept and used by the research team.	☐
4. I give permission for my General Practitioner (GP) and my Early Intervention team to be told I am participating in this study.	☐
5. I understand that relevant sections of my medical notes and data collected during the study may be looked at by regulatory authorities or from the NHS Trust, where it is relevant to my taking part in this research. I give permission for these individuals to have access to my records.	☐
6. I understand that I will be given a £20 gift as cash for my participation in each study assessment.	☐
7. I agree to the research team consulting NHS electronic records to investigate my diagnosis, medication, and mental health service use, and give them permission to do so even if I choose to no longer participate in the intervention, or they are not able to carry out further study interviews with me.	☐
8. I understand that in the event that I disclose information which may indicate new risk to myself or others, the researcher will be obliged to follow NHS Trust risk procedures that may require release of my personal data.	☐
9. I give permission for findings from the study to be written up for publication. Any publication will not identify me.	☐
10. I give permission to be audio recorded where required for the purposes of the study. I understand these audio-recordings will be transcribed and anonymised and audio recordings destroyed after the study. I give permission for direct quotations taken from this interview to be included in papers written for publication. Any quotation would not identify me.	☐
11. I give permission for the research team to collect data from the My Journey 3 app regarding the frequency, duration, and pattern of my use of it. I understand that no personal information will be collected from the app.	☐
12. I give permission for non-identifiable data to be shared with other research teams for research purposes.	☐

For peer review only - http://bmjopen.bmj.com/site/about/guidelines.xhtml

13. I agree to take part in this study. ☐

Name of participant	Date	Signature
Name of Researcher taking consent	Date	Signature

For peer review only

For peer review only - http://bmjopen.bmj.com/site/about/guidelines.xhtml

CONSORT 2010 checklist of information to include when reporting a pilot or feasibility trial*

Section/Topic	Item No	Checklist item	Reported on page No
**Title and abstract**			
	1a	Identification as a pilot or feasibility randomised trial in the title	Title Page
	1b	Structured summary of pilot trial design, methods, results, and conclusions (for specific guidance see CONSORT abstract extension for pilot trials)	Abstract
**Introduction**			
Background and objectives	2a	Scientific background and explanation of rationale for future definitive trial, and reasons for randomised pilot trial	Introduction
	2b	Specific objectives or research questions for pilot trial	Introduction
**Methods**			
Trial design	3a	Description of pilot trial design (such as parallel, factorial) including allocation ratio	Methods (design)
	3b	Important changes to methods after pilot trial commencement (such as eligibility criteria), with reasons	N/A
Participants	4a	Eligibility criteria for participants	Methods (participants)
	4b	Settings and locations where the data were collected	Methods (setting)
	4c	How participants were identified and consented	Methods (recruitment strategy)
Interventions	5	The interventions for each group with sufficient details to allow replication, including how and when they were actually administered	Methods (interventions)
Outcomes	6a	Completely defined prespecified assessments or measurements to address each pilot trial objective specified in 2b, including how and when they were assessed	Methods (outcomes)
	6b	Any changes to pilot trial assessments or measurements after the pilot trial commenced, with reasons	N/A
	6c	If applicable, prespecified criteria used to judge whether, or how, to proceed with future definitive trial	N/A
Sample size	7a	Rationale for numbers in the pilot trial	Methods (participants)

For peer review only

	7b	When applicable, explanation of any interim analyses and stopping guidelines	Methods (analysis)
Randomisation:			
Sequence generation	8a	Method used to generate the random allocation sequence	Methods (randomisation)
	8b	Type of randomisation(s); details of any restriction (such as blocking and block size)	Methods (randomisation)
Allocation concealment mechanism	9	Mechanism used to implement the random allocation sequence (such as sequentially numbered containers), describing any steps taken to conceal the sequence until interventions were assigned	Methods (randomisation)
Implementation	10	Who generated the random allocation sequence, who enrolled participants, and who assigned participants to interventions	Methods (randomisation)
Blinding	11a	If done, who was blinded after assignment to interventions (for example, participants, care providers, those assessing outcomes) and how	Methods (randomisation)
	11b	If relevant, description of the similarity of interventions	N/A
Statistical methods	12	Methods used to address each pilot trial objective whether qualitative or quantitative	Methods (analysis)
**Results**			
Participant flow (a diagram is strongly recommended)	13a	For each group, the numbers of participants who were approached and/or assessed for eligibility, randomly assigned, received intended treatment, and were assessed for each objective	Results (feasibility of trial design)
	13b	For each group, losses and exclusions after randomisation, together with reasons	Results (feasibility of trial design)
Recruitment	14a	Dates defining the periods of recruitment and follow-up	Results (feasibility of trial design)
	14b	Why the pilot trial ended or was stopped	N/A
Baseline data	15	A table showing baseline demographic and clinical characteristics for each group	Results (sample characteristics)
Numbers analysed	16	For each objective, number of participants (denominator) included in each analysis. If relevant, these numbers should be by randomised group	Results (participant outcomes)

For peer review only

Outcomes and estimation	17	For each objective, results including expressions of uncertainty (such as 95% confidence interval) for any estimates. If relevant, these results should be by randomised group	Results (participant outcomes)
Ancillary analyses	18	Results of any other analyses performed that could be used to inform the future definitive trial	N/A
Harms	19	All important harms or unintended effects in each group (for specific guidance see CONSORT for harms)	Results (participant outcomes)
	19a	If relevant, other important unintended consequences	N/A
**Discussion**			
Limitations	20	Pilot trial limitations, addressing sources of potential bias and remaining uncertainty about feasibility	Discussion (strength and limitations)
Generalisability	21	Generalisability (applicability) of pilot trial methods and findings to future definitive trial and other studies	Discussion (strength and limitations)
Interpretation	22	Interpretation consistent with pilot trial objectives and findings, balancing potential benefits and harms, and considering other relevant evidence	Discussion (conclusions)
	22a	Implications for progression from pilot to future definitive trial, including any proposed amendments	Discussion (conclusions)
**Other information**			
Registration	23	Registration number for pilot trial and name of trial registry	Abstract
Protocol	24	Where the pilot trial protocol can be accessed, if available	Additional file 3
Funding	25	Sources of funding and other support (such as supply of drugs), role of funders	Funding
	26	Ethical approval or approval by research review committee, confirmed with reference number	Methods (design)

Citation: Eldridge SM, Chan CL, Campbell MJ, Bond CM, Hopewell S, Thabane L, et al. CONSORT 2010 statement: extension to randomised pilot and feasibility trials. BMJ. 2016;355.
*We strongly recommend reading this statement in conjunction with the CONSORT 2010, extension to randomised pilot and feasibility trials, Explanation and Elaboration for important clarifications on all the items. If relevant, we also recommend reading CONSORT extensions for cluster randomised trials, non-inferiority and equivalence trials, non-pharmacological treatments, herbal interventions, and pragmatic trials. Additional extensions are forthcoming: for those and for up to date references relevant to this checklist, see www.consort-statement.org.

For peer review only

BMJ Open

Smartphone-delivered self-management for first-episode psychosis: the ARIES feasibility randomised controlled trial

Journal:	*BMJ Open*
Manuscript ID	bmjopen-2019-034927.R2
Article Type:	Original research
Date Submitted by the Author:	13-Mar-2020
Complete List of Authors:	Steare, Thomas; University College London, Division of Psychiatry<br>O'Hanlon, Puffin; University College London, Division of Psychiatry<br>Eskinazi, Michelle; University College London, Division of Psychiatry<br>Osborn, David; University College London, Division of Psychiatry; Camden and Islington NHS Foundation Trust<br>Lloyd-Evans, Brynmor ; University College London, Division of Psychiatry; Camden and Islington NHS Foundation Trust<br>Jones, Rebecca; University College London, Division of Psychiatry<br>Rostill, Helen; University of Surrey; Surrey and Borders Partnership NHS Foundation Trust<br>Amani, Sarah; Early Intervention in Psychosis Programme (South of England)<br>Johnson, Sonia; University College London, Division of Psychiatry; Camden and Islington NHS Foundation Trust
**Primary Subject Heading**:	Mental health
Secondary Subject Heading:	Health services research
Keywords:	Schizophrenia & psychotic disorders < PSYCHIATRY, MENTAL HEALTH, Clinical trials < THERAPEUTICS

SCHOLARONE™
Manuscripts

I, the Submitting Author has the right to grant and does grant on behalf of all authors of the Work (as defined in the below author licence), an exclusive licence and/or a non-exclusive licence for contributions from authors who are: i) UK Crown employees; ii) where BMJ has agreed a CC-BY licence shall apply, and/or iii) in accordance with the terms applicable for US Federal Government officers or employees acting as part of their official duties; on a worldwide, perpetual, irrevocable, royalty-free basis to BMJ Publishing Group Ltd ("BMJ") its licensees and where the relevant Journal is co-owned by BMJ to the co-owners of the Journal, to publish the Work in this journal and any other BMJ products and to exploit all rights, as set out in our licence.

The Submitting Author accepts and understands that any supply made under these terms is made by BMJ to the Submitting Author unless you are acting as an employee on behalf of your employer or a postgraduate student of an affiliated institution which is paying any applicable article publishing charge ("APC") for Open Access articles. Where the Submitting Author wishes to make the Work available on an Open Access basis (and intends to pay the relevant APC), the terms of reuse of such Open Access shall be governed by a Creative Commons licence – details of these licences and which Creative Commons licence will apply to this Work are set out in our licence referred to above.

Other than as permitted in any relevant BMJ Author's Self Archiving Policies, I confirm this Work has not been accepted for publication elsewhere, is not being considered for publication elsewhere and does not duplicate material already published. I confirm all authors consent to publication of this Work and authorise the granting of this licence.

For peer review only

TITLE PAGE

Title: Smartphone-delivered self-management for first-episode psychosis: the ARIES feasibility randomised controlled trial

Authors:

Thomas Steare

Division of Psychiatry, University College London, London, UK

Puffin O'Hanlon

Division of Psychiatry, University College London, London, UK

Michelle Eskinazi

Division of Psychiatry, University College London, London, UK

David Osborn

Division of Psychiatry, University College London, London, UK

R&D Department, Camden and Islington NHS Foundation Trust, London, UK

Brynmor Lloyd-Evans

Division of Psychiatry, University College London, London, UK

R&D Department, Camden and Islington NHS Foundation Trust, London, UK

Rebecca Jones

Division of Psychiatry, University College London, London, UK

Helen Rostill

University of Surrey, UK

Surrey and Borders Partnership NHS Foundation Trust, Leatherhead, Surrey, UK

Sarah Amani

EIP Programme (South of England), NHS England, Oxford, Oxfordshire, UK

For peer review only

Sonia Johnson (corresponding author)

Address: UCL Division of Psychiatry, 6th Floor, Wing B, Maple House, 149 Tottenham Court Road, London W1T 7NF

Division of Psychiatry, University College London, London, UK

R&D Department, Camden and Islington NHS Foundation Trust, London, UK

s.johnson@ucl.ac.uk

02076799453

Word count: 5,937

Keywords: Psychosis, Self-management, Smartphone, Feasibility study, Randomised controlled trial

For peer review only

ABSTRACT

Objectives: To test the feasibility and acceptability of a randomised controlled trial (RCT) to evaluate a Smartphone-based self-management tool in Early Intervention in Psychosis (EIP) services.

Design: A two-arm unblinded feasibility RCT.

Setting: Three NHS EIP services in England.

Participants: Adults using EIP services that own an Android Smartphone. Participants were recruited until the recruitment target was met (n=40).

Interventions: Participants were randomised with a 1:1 allocation to one of two conditions: (1) treatment as usual from EIP services (TAU) or (2) TAU plus access to My Journey 3 on their own Smartphone. My Journey 3 features a range of self-management components including access to digital recovery and relapse prevention plans, medication tracking and symptom monitoring. My Journey 3 use was at the users' discretion, and was supported by EIP service clinicians. Participants had access for a median of 38.1 weeks.

Primary and secondary outcome measures: Feasibility outcomes included recruitment, follow-up rates and intervention engagement. Participant data on mental health outcomes were collected from clinical records and from research assessments at baseline, 4 months and 12 months.

Results: 83% and 75% of participants were retained in the trial at the 4- and 12-month assessments. All treatment group participants had access to My Journey 3 during the trial, but technical difficulties caused delays in ensuring timely access to the intervention. The median

For peer review only

number of My Journey 3 uses was 16.5 (IQR 8.5 to 23) and median total minutes spent using My Journey 3 was 26.8 (IQR 18.3 to 57.3). No serious adverse events were reported.

Conclusions: Recruitment and retention were feasible. Within a trial context My Journey 3 could be successfully delivered to adults using EIP services, but with relatively low usage rates. Further evaluation of the intervention in a larger trial may be warranted, but should include attention to implementation.

Trial Registration: ISRCTN10004994

ARTICLE SUMMARY

Strengths and limitations of this study:

- Participant data was collected from a wide range of sources including questionnaires, patient records and from the app
- Participants were followed up for a 12-month period; longer than the majority of feasibility trials investigating Smartphone apps for psychosis
- We were not able to blind researchers or participants to their treatment allocation
- The study recruited users of Early Intervention in Psychosis services that own an Android Smartphone, limiting sample representativeness
- This is a feasibility study, and therefore does not have the statistical power to conclude the effectiveness of the intervention.

For peer review only

INTRODUCTION

Early Intervention in Psychosis (EIP) services have been established across the United Kingdom to provide care to adults during the three years following an initial episode of psychosis. There is evidence that such services are effective and cost-effective,[1,2] resulting in improvement in a range of outcomes yet challenges remain. Relapse rates for EIP service users are high [3] particularly after discharge [4,5] and limited adherence with antipsychotic medication is common [6]. There are also difficulties accessing psychosocial interventions,[7] including supported self-management.

Illness self-management is an approach designed to support people to manage long-term health conditions by developing their ability to recognise and monitor symptoms and early warning signs of relapse, identify and avoid stressors, make plans for achieving their own recovery and to effectively use coping strategies.[8] For people with psychosis, self-management tools have been shown to reduce psychological distress, improve medication adherence and reduce the likelihood of future hospital admissions.[9-11] In a recent meta-analysis, self-management interventions for severe mental illness were also found to have a significant benefit on patient-valued outcomes of personal recovery, hope and self-efficacy.[12] Despite clinician-supported self-management programmes being mandated in current UK treatment guidelines for first-episode psychosis,[13] there is a lack of well-evaluated tools to support delivery within EIP services. There is a clear need to overcome implementation barriers affecting the delivery of self-management to those likely to benefit from it.[12] A potentially convenient and economical way of achieving this is via the use of digital technology such as Smartphones.[14]

Smartphones can run advanced software known as apps that hold promise as an effective tool to assist the monitoring and treatment of mental health problems. Smartphone ownership is

For peer review only

rapidly growing world-wide [15] with a significant number of developed countries with ownership rates of more than 80%.[16] Adults with severe mental health problems have comparable Smartphone ownership rates to the general population,[17-19] and there is a growing consensus that adults with psychosis are open to using Smartphones to access mental health interventions.[20,21] Smartphones also provide high accessibility to the internet and are commonly carried on the person, meaning apps can be easily accessed at times and locations convenient for the user. Accordingly Smartphones have the capacity to deliver time-unlimited mental health interventions, such as self-management tools, and ultimately the potential to increase access to effective care and reduce healthcare costs.[22] The benefits of Smartphone apps may also extend beyond the original treatment period with a community team, and could be a valuable tool following discharge where the risk of relapse is increased.[4,5]

The majority of digital health interventions that have been developed for psychosis have been based on existing psychological therapies such as Cognitive Behavioural Therapy,[23,24] or other evidence-based interventions,[25,26] yet very little is known regarding their effectiveness when delivered in EIP services. A growing number of self-management apps for psychosis have been tested for feasibility and acceptability, including those delivered independent of a clinical setting and those embedded within clinical care.[27-29] These have shown promising levels of adoption and use in research contexts yet little is known about their clinical efficacy.

To date only one trial of a self-management app delivered in EIP services has published results regarding the interventions impact on clinical outcomes.[30] In the proof-of-concept trial an active self-management app "Actissist" was found to confer benefits over a passive control app. The study suggests that participants that received Actissist had better outcomes regarding their mood and general and negative symptoms post-treatment in comparison to control participants.

For peer review only

Actissist features a range of components including self-assessment questions focused on cognitive appraisals, emotions, behaviours and belief convictions and suggests appropriate coping strategies, but does not feature some major cornerstones of self-management such as relapse and recovery plans. Regardless results from this study suggest that such digital self-management interventions could potentially improve outcomes of people using EIP services. Further trials are needed before firm conclusions can be made regarding the feasibility of conducting RCTs in this field and of the therapeutic benefits of self-management apps for first-episode psychosis delivered in clinical settings.

We aimed to address this evidence gap by conducting a feasibility RCT of a supported self-management Smartphone app, "My Journey 3" designed to help EIP service users recognise early warning signs of illness, recognise and monitor symptoms and create plans for their recovery. My Journey 3 has been designed to be initially set up in EIP services and used with clinician support, but to also be suitable for independent use. The results of the feasibility RCT are a potential step towards a full-scale trial to assess the effectiveness of the intervention.

The objectives of this study were as follows:

1. To determine the acceptability of the My Journey 3 self-management app for use in an EIP service context
2. To determine the feasibility of trial procedures for a definitive trial, including recruitment, intervention enrolment and trial attrition.
3. To test procedures for evaluating intervention engagement and participant outcomes.

For peer review only

METHODS

Design

The App to support Recovery in Early Intervention Services (ARIES) study was an unblinded feasibility RCT with a nested qualitative study comparing a supported self-management Smartphone app (My Journey 3) in addition to Treatment As Usual (TAU), with a control group receiving TAU only. Participants were randomly allocated to one of the two trial arms in a 1:1 ratio. Since this was a feasibility trial, it was not designed to have sufficient statistical power to assess the effectiveness of the My Journey 3 intervention.

Ethical approval was obtained from the London Brent National Research Ethics Service Committee (Ref 15/LO/1453). The trial was retrospectively registered (ISRCTN10004994). As the study was a feasibility trial prospective registration was not required.[31] Further details of the methodology are available in the protocol paper.[32] We have followed the Consolidated Standards of Reporting Trials (CONSORT) statement extension for pilot and feasibility randomised trials for reporting.[33] A copy of the CONSORT checklist is provided as Additional file 1.

Setting

The trial was conducted in six EIP services across three NHS Foundation Trusts in England. EIP services are multi-disciplinary community mental health services that provide care coordination to people in the first three years of a first-episode psychosis, focusing on engagement, achieving social and clinical recovery and delivering a full range of pharmacological, psychological and social interventions.[34] The six EIP services as mandated in England provide care for people up to the age of 65, with the potential for adults above the age range to access EIP services if clinically appropriate although these cases are rare . Two of the participating Trusts are

located in inner London. The third Trust is located in a county outside of London with both urban and rural areas. Assessments were conducted face-to-face at EIP services, at participants' homes or at University College London.

Participants

Participants were recruited from the participating EIP services over seven months. We assumed a conservative 40% attrition rate and accordingly set the target sample size as 40 participants to ensure the trial retained twelve completer participants per group (as recommended to assess trial feasibility).[35] Participants were eligible if they were aged ≥16 years, had experienced at least one episode of psychosis, were currently on the caseload of an EIP service and owned a Smartphone with an Android operating system. People were excluded from the trial if they lacked capacity to consent to participation, were unable to communicate and understand English, or were considered by their EIP service to pose a high risk to researchers during meetings, even on NHS premises. Familiarity and competence in using digital technology or Smartphones was not an eligibility criterion.

Recruitment strategy

Clinicians at the participating EIP services were briefed by the research team, and were asked to make initial contact with eligible EIP service users. Clinicians explained the trial to service users, and enquired whether the service user would be willing to speak to a researcher about participating in the trial. The researcher then made contact with eligible and potentially willing service users, and arranged a face-to-face meeting where the trial was explained further. The researcher provided the trial information sheet (Additional file 2), and assessed the participant's capacity to provide informed consent. Service users had at least 24 hours after

For peer review only

receiving the information sheet to consider their participation. Participants then gave written informed consent to take part, prior to completing the baseline assessment. No participants were recruited via online methods.

Randomisation

Following the baseline assessment, participants were randomly allocated in a 1:1 ratio to either the intervention (n=20) or the control group (n=20) by an independent statistician. The treatment group had access to My Journey 3 in addition to TAU, whilst the control group received TAU only. An independent researcher held the allocation list and did not disclose participants' allocation to the trial researcher until after completion of the baseline assessments, allowing the researcher to remain blinded during recruitment and whilst carrying out the baseline assessments.

Due to the nature of the intervention, participants were not blinded to their group allocation. During the recruitment process, participants would have been aware that My Journey 3 was the intervention of interest. As a single researcher carried out the majority of data collection, it was not practical for the allocation of participants to be concealed from the research team. Participants were informed of their allocation by the researcher via a telephone call.

Interventions

My Journey 3

My Journey 3 is a Smartphone app developed for adults accessing EIP services. The aim of the intervention is to develop users' self-management skills to help them to achieve self-determined recovery goals and avoid future relapses. My Journey 3 is suitable for independent use, but also designed to be used with support from EIP service clinicians who will be able to assist with the completion of the self-management components and initial set-up. It is the developers'

For peer review only

aspiration for My Journey 3 to be used initially in collaboration with EIP service clinicians, and for it to support continuing self-management after users have been discharged from EIP services.

The development of My Journey 3 has been through several iterations. The first version (My Journey 1) was created by Surrey and Borders Partnership NHS Foundation Trust with leadership from Sarah Amani, for EIP service users to track their symptoms, set reminders for appointments and share their progress with EIP service clinicians. In developing the current version of My Journey 3 we have drawn on existing paper-and-pen self-management intervention components [36,37] to allow users to track recovery goals and personalise relapse prevention plans – important cornerstones of illness self-management. The design and the content of My Journey 3 was led by a collaboration of researchers, digital health experts, EIP service clinicians and service users. A private app development company based in the UK (MyOxygen; https://myoxygen.uk) led the technical development of My Journey 3. To limit costs My Journey 3 is only compatible with Smartphones with Android operating systems at this stage of testing.

My Journey 3 features four key elements of self-management, an approach with demonstrated efficacy in improving social and clinical outcomes for people with psychosis.[12] Screenshots of the key components are displayed in figure 1. Users have the ability to create a relapse prevention plan, where there is the opportunity to identify and list triggers, early warning signs of relapse and personalised coping strategies to refer to as required and to create a plan to follow if experiencing a crisis. Via the 'My Recovery Plan' section users are able to set recovery goals, list actions they can do to encourage well-being, and set reminders on their Smartphone to encourage engagement in these activities. Users can also use a tracker to monitor and rate their symptoms and early warning signs over time. In the Symptom Tracker users are presented with seventeen different symptoms and behaviours and are asked to respond via a "Yes/No" format as

For peer review only

to whether they have recently experienced these. Users that respond with a “Yes” are then presented with a 10-point scale (4-point scale for the early warning sign tracker) to rate the severity or frequency of the associated symptoms, with advice on how to manage these symptoms displayed. Psycho-education on mental health, medication and mental health services is provided in an ‘Information’ section. To encourage adherence with medication, users are encouraged to log and track their medication in the ‘Pill Tracker’ section. Users are able to set daily alerts to remind them to log whether they have taken their medication. My Journey 3 also features weekly discrete notifications to encourage engagement with the app, which can be disabled at the users’ preference. The key components of My Journey 3 are summarized in Table 1, with further details available in the protocol paper.[32]

Prior to the feasibility trial reported in this paper, My Journey 3 was tested by EIP service users in lab-based usability tests and in a one-month field study. The final content of My Journey 3 was then refined based on feedback from individual interviews with the participating EIP service users and clinicians. No changes were made to the content of My Journey 3 during the feasibility RCT. A major technical update to My Journey 3 was carried out in January 2018 to fix compatibility issues with older versions of Android operating systems. This did not require any changes to the trial design.

For peer review only

Table 1. Key sections of the My Journey 3 Smartphone app.

Section	Features	Purpose
My recovery plan	Things I can do to keep well<br><br>My goals	To encourage users to have regular routines, track activities, set reminders and plan how to achieve long-term goals
My relapse prevention plan	Coping with triggers<br><br>Coping with early warning signs<br><br>Coping with a crisis<br><br>Crisis contacts	To help users' identify, monitor and cope with triggers and early warning signs<br><br>To help users create a "relapse plan" to follow in times of crisis
How are you doing?	My mood<br><br>My early warning signs<br><br>My tracker	For users to monitor symptoms, behaviours and early warning signs and track these experiences over time
Pill tracker		To log whether users' have taken their medication each day
Information	Medication information<br><br>Useful websites<br><br>Emergency services<br><br>Jargon buster	To provide users with useful information and external links on medication and mental health<br><br>To identify local emergency services in a time of crisis<br><br>To provide a glossary of terms that are commonly used in mental health care

Delivery

Following assignment to the treatment group, participants engaged in individual training sessions with a trial researcher and a supporting EIP service clinician. Training sessions were intended to take place within six weeks of the participants' initial baseline assessment, and lasted

For peer review only

for approximately 2 hours. During these sessions the researcher downloaded My Journey 3 onto the participants' Smartphone and gave a demonstration of the app and its main functions. Participants were then encouraged to input appropriate information to specific sections of My Journey 3 with the help of the supporting EIP service clinician in attendance. Following this session it was hoped that all participants had initial personal recovery plans, relapse prevention plans and crisis plans stored on My Journey 3.

Participants had access to My Journey 3 on their own Smartphone from the training session till the 12-month time-point. Researchers recommended that participants used My Journey 3 at least once a week, but participants had a free choice in how and when they used My Journey 3. Participants did not receive any financial incentives to use My Journey 3, and were free to withdraw from using the app or decline the installation of it on to their Smartphone. At the training session participants were informed by the researcher that My Journey 3 would be not suitable for seeking urgent medical care whilst in crisis, and that it is not a substitute for human support.

To encourage user engagement with My Journey 3 during the trial, supporting EIP service clinicians were asked to provide regular support and encouragement to service users who had access to My Journey 3. Clinicians were asked to discuss recovery goals and relapse prevention plans in routine appointments with participants, and assist with entering these into the appropriate My Journey 3 sections. Clinicians had an existing understanding of self-management approaches from their clinical training and practice, and would be able to provide appropriate advice with the intervention components of My Journey 3 but they received no formal training on how to implement My Journey 3 into their clinical work. Clinicians' understanding of operating My Journey 3 was from the training sessions only. Clinician support for My Journey 3 as part of the trial was not manualised or incentivised.

For peer review only

Participants were encouraged to contact the trial researcher in the case of technical problems with My Journey 3. The researcher contacted participants a week after the training session to check that My Journey 3 had been functioning without issues, and invited any questions about the app. No further prompts were instigated by the researcher during the trial.

Treatment as usual

All participants received TAU regardless of group allocation. TAU for a person under the care of EIP services typically involves regular meetings with a care co-ordinator, access to a psychiatrist, psychiatric medication, and a range of psychological interventions. EIP services are encouraged to deliver self-management programmes, that includes advice on symptom management, crisis planning and relapse prevention, generally delivered with paper-and-pen tools if at all.[34] None of the participating EIP services offered digital interventions or Smartphone apps as part of routine care during the study period, and structured self-management support, including the relapse prevention work recommended in EIP contexts, is inconsistently implemented.

Patient and Participant Involvement

The development of My Journey 3 has been guided by the input of people with lived experience of psychosis. Initial development of the design and content involved a collaboration between researchers, experts in digital health and service users. Service users provided further input into the design and functionality of My Journey 3 from providing feedback after taking part in lab-based tests and a field study.

For peer review only

Outcomes

Participant data were collected from numerous sources including participant assessments, patient records and anonymous My Journey 3 usage reports. There were no pre-specified criteria for assessing trial feasibility and intervention acceptability.

Questionnaire measures

Proposed outcome measures for a future trial were assessed at structured face-to-face assessments with a trained researcher at three time points; baseline, 4-months post baseline and 12-months post baseline. At all meetings participants completed self-report questionnaires that have been previously used with people with first-episode psychosis. Participants were given £20 as a thank you for completing the assessment at each time point.

At each assessment we collected sociodemographic data including age, gender, ethnicity, accommodation and living situation, employment status, educational attainment, Smartphone use, and use of other mental health apps. The following self-report measures were also collected: social outcomes (Social Outcomes Index (SIX),[38] score 0-6: higher score= better social outcomes), self-efficacy (Mental Health Confidence Scale (MHCS),[39] score 16-96: higher score= greater empowerment), self-rated recovery (Questionnaire about the Process of Recovery (QPR),[40] intrapersonal score 0-68, interpersonal score 0-20: higher score= greater recovery), mental well-being (Warwick-Edinburgh Mental Well-Being Scale (WEMWBS),[41] score 14-70: higher score= greater well-being) and quality of life and satisfaction with treatment (DIALOG scale,[42] score 1-7: higher score= greater quality of life/satisfaction with treatment).

For peer review only

Clinical structured interviews were also conducted with each participant by the researcher, to assess psychopathology, using the Positive and Negative Syndrome Scale (PANSS).[43] Higher PANSS scores are indicative of greater severity of each symptom domain.

Participants' engagement with EIP services were measured using the Service Engagement Scale (SES),[44] completed by EIP service clinicians known to each participant, typically care co-coordinators. Clinicians completed the SES at baseline and 12 months later, regardless of whether participants attended the 12-month assessment. Higher SES scores are indicative of poorer user engagement with EIP services.

Patient records

Clinical data were extracted from patient records at baseline and at the 12-month time point. Clinical measures included most recent diagnosis and use of EIP services, other community mental health teams and acute mental health services in the previous 12 months.

The proposed primary outcome for a future RCT (relapse of psychosis) was operationalised as an admission to an acute mental health service (inpatient psychiatric ward, crisis house, crisis resolution team or acute day care service) during the 12-month trial period as indicated in patient records. This definition of relapse has been used previously in a recent trial of a self-management intervention.[45]

My Journey 3 use

To assess acceptability of the intervention and user engagement, My Journey 3 usage data were collected for all participants in the treatment group from the training session until the 12-month time-point. Whenever users had Wi-Fi internet access on their Smartphone My Journey 3 automatically uploaded encrypted usage data to a secure server. Data collected included a record

For peer review only

of each time the user opened My Journey 3, whether this was in response to a prompt, and which components they used. To ensure confidentiality, personal or identifiable data such as text or responses to each sections were not collected.

Analysis

Participant demographic and clinical characteristics, My Journey 3 usage, and rates of participant recruitment and retention were summarised using descriptive statistics. As this was a feasibility RCT, it was not powered to assess the effectiveness of the intervention. Statistical analyses of participant outcome measures were conducted to pilot the methods of analysis for a fully powered effectiveness trial. Logistic regression was used to explore the impact of the My Journey 3 intervention on relapse. Linear regression was used to examine the potential effect of the intervention on continuous outcome measures at 4 months and 12 months separately. We report the effect estimates and corresponding 95% confidence intervals (CI) only for unadjusted analyses and for analyses adjusting for the baseline measure of the outcome in question. All analyses were performed using STATA V.14 after completion of the final participant assessment. No interim analyses were conducted.

RESULTS

Feasibility of trial design

Participant flow is detailed in the CONSORT diagram (figure 2). A total of 40 participants was recruited and randomised (20 to My Journey 3, 20 to TAU) over a 7-month period from March 2017 to September 2017. Participants were recruited until the required number of 40 was obtained: we do not therefore have a full assessment of the proportion of the teams' caseload who could have

For peer review only

been recruited to a full trail, nor do we know the proportion of approached EIP services users that did not meet eligibility criteria or declined involvement in the trial.

Among those recruited to the trial, attrition rates were generally low: 83% (33/40) and 75% (30/40) of participants successfully attended and completed follow-ups at 4 months and 12 months respectively. At both time points the follow-up rate was lower in the control group (4-months: 65% compared to 100%, 12-months: 70% compared to 80%). Patient record data were available for all participants at baseline and for 95% of the sample (38/40) at the 12-month time-point. Completion rates of the SES by clinicians were higher at baseline (90%) than at the 12-month time-point (67.5%). Follow-up assessments were conducted from July 2017 to October 2018.

All participants in the treatment group attended a training session with a researcher, and had access to My Journey 3 during the trial. Issues with Smartphone compatibility initially prevented three participants from downloading My Journey 3. Following an update to the system two of the participants were able to install and access My Journey 3 on their own Smartphones. Two participants were provided with Smartphones with My Journey 3 pre-installed (the app was still incompatible on one participant's Smartphone despite the update; another participant no longer owned an Android Smartphone after entering the trial). The median length of time from trial enrolment to having access to My Journey 3 was 14 weeks (IQR 11 to 17), longer than the planned time of 6 weeks. Participants had access to My Journey 3 for a median of 38.1 weeks (IQR 34.8 to 40.7). There were no reported privacy breaches.

My Journey 3 usage data were collected for all participants following the training session, with 500 different data entries available for analysis. Within the 500 data entries, 27 (5.4%) were corrupt and were subsequently removed from the analysis. The unusable data can grouped into two types. The first, duplicates of previous data entries that were subsequently removed. The

For peer review only

second, entries where the times were implausible (for example, the end time of using My Journey 3 was recorded as occurring before the start time). In addition, a further issue caused errors with accurately recording My Journey 3 usage data of ‘My Recovery Plan’ and ‘My Relapse Plan’ sections. As a result we were unable to accurately conclude how often participants used these sections.

One participant randomised to the control group was wrongly given access to My Journey 3. For the purpose of the statistical analysis they are classed as a control participant.

Sample characteristics

A summary of demographic and clinical characteristics of the sample is displayed in Table 2. The sample was predominantly male (n=28, 70%). The mean age of the sample was 29.7 years (SD = 9.78) and similar to that of UK cohorts of EIP service users at first presentation.[46,47] Most participants had a diagnosis of a schizophrenia, schizotypal or delusional disorder (ICD code F20-F29) and were not in paid employment. A quarter of the sample (n=10, 25%) had completed a university degree. Eight (20%) participants had previously used a mental health app.

For peer review only

Table 2. Key demographic and clinical characteristics of the sample at baseline.

	Control (n=20)	My Journey 3 (n=20)
**Age (years) – mean (SD), [min, max]**	30 (10.1), [18.8, 64.7]	29.4 (9.7), [17.6, 52.4]
**Gender**		
Female	7 (35%)	5 (25%)
**Ethnicity**		
White British	6 (30%)	8 (40%)
Any other white/Mixed white	2 (10%)	1 (5%)
Black African	5 (25%)	3 (15%)
Black Caribbean	1 (5%)	1 (5%)
Black Other	1 (5%)	0
Asian Indian	1 (5%)	0
Asian Other	1 (5%)	2 (10%)
Other/Mixed other	3 (15%)	3 (15%)
**Education**		
Undergraduate degree	6 (30%)	4 (20%)
Some University but no degree	3 (15%)	2 (10%)
Higher National Degree or professional qualification	2 (10%)	1 (5%)
A Levels or equivalent	3 (15%)	4 (20%)
GCSEs or equivalent	4 (20%)	6 (30%)
No qualifications	1 (5%)	3 (15%)
Missing	1 (5%)	0
**Employment status**		
Employed – more than 16 hours a week	4 (20%)	4 (20%)
Employed – less than 16 hours a week	0	2 (10%)
Voluntary work	3 (15%)	3 (15%)
In study or training	1 (5%)	1 (5%)
Unemployed or exempt due to disability	8 (40%)	8 (40%)
Missing	4 (20%)	2 (10%)
**Primary diagnosis (ICD-10 code)**		
F10-F19: Mental and behavioural disorder due to psychoactive substance use	1 (5%)	0
F20-F29: Schizophrenia, schizotypal and delusional disorder	16 (80%)	13 (65%)
F30-F39: Mood disorder	1 (5%)	5 (25%)
Missing	2 (10%)	2 (10%)
**Admission to an acute mental health service in previous year**		
Yes	11 (55%)	10 (50%)
**SIX – mean (SD), [min, max]**	3.2 (1.5), [0, 6]	3.6 (1.5), [1, 6]
**MHCS – mean (SD), [min, max]**	59.7 (17.8), [16, 82]	61.2 (12.6), [38, 78]
**QPR – mean (SD), [min, max]**		
Intrapersonal	45.7 (12), [22, 68]	42.2 (10.6), [24, 60]
Interpersonal	13.7 (2.7), [9, 19]	12.9 (3.4), [5, 19]
**WEMWBS – mean (SD), [min, max]**	43.4 (11.6), [25, 69]	40.3 (10.2), [23, 57]
**DIALOG – mean (SD), [min, max]**		
Quality of life	4.5 (1), [2.8, 6.5]	4.4 (0.8), [3, 5.7]
Treatment satisfaction	5.4 (0.7), [4.3, 7]	4.8 (0.7), [3.7, 6]
**PANSS – mean (SD), [min, max]**		
Positive	10.9 (5), [7, 22]	11.3 (4.2), [7, 19]
Negative	10.7 (2.5), [7, 19]	11.8 (4.5), [7, 20]
General	26.6 (6), [17, 39]	26.2 (8), [16, 46]
**SES – mean (SD), [min, max]**	11.3 (7.9), [0, 26]	9.6 (7), [0, 23]

For peer review only

All statistics are reported N (%) unless otherwise specified. Missing data: PANSS scores – one control group participant, SES – three control group participants, one treatment group participant.

My Journey 3 use

The level of My Journey 3 use was highly skewed. The median number of times My Journey 3 was used per participant during the trial was 16.5 (IQR 8.5 to 23). Participants accessed My Journey 3 a median of 3.22% (IQR 1.89 to 6.36) of the days it was available to them, equating to My Journey 3 being used on average once every 31 days (IQR 15.7 to 52.9). Participants spent a median of 26.8 minutes (IQR 18.3 to 57.3) in total using My Journey 3 over the course of the trial. Eight participants (40%) used My Journey 3 for longer than 30 minutes in total.

Five participants (25%) were still using My Journey 3 six months after downloading it, however one participant never used the app after the training session (figure 3). Half of the participants (n=10) stopped using My Journey 3 within the first three months after the training session.

The average number of uses by participants for each My Journey 3 component is displayed in table 3. The most frequently accessed section was the "How are you doing?" Symptom Tracker section (median uses 3; IQR 1 to 6), however data on how frequently users accessed 'My Recovery Plan' and 'My Relapse Plan' is unavailable. The 'Information' section was accessed the fewest times, with 25% (n=5) of participants in the treatment group never using that section following the training session. Just over 7% of My Journey 3 uses were initiated following a prompt from the app.

For peer review only

Table 3. Participant use of My Journey 3 and various sections.

	Number of times used per participant	Days used whilst having access to My Journey 3 (%)	Participants that did not use app or section – n (%)
My Journey 3	16.5 (8.5 to 23)	3.22 (1.89 to 6.36)	1 (5%)
How are you doing?	3 (1 to 6)	1.08 (0.4 to 2.12)	3 (15%)
Pill tracker	2 (1 to 3.5)	0.73 (0.36 to 1.07)	3 (15%)
Information	1 (0 to 2.5)	0.48 (0.18 to 0.7)	5 (25%)

All median (IQR), except when stated

Participant outcomes

No research-related serious adverse events were recorded. Psychotic and general symptoms (measured by the PANSS) were generally low at all times for both groups suggesting a stable sample. Summary statistics and estimated effect sizes of participant outcomes are displayed in table 4. Inspection of the effect sizes and confidence intervals suggest that were no obvious differences for any outcome measure between the treatment and control group at either time-point.

Of the 38 participants whose patient records data were available, only five experienced a relapse during the trial, as indicated by using an acute mental health service. In the treatment group 15% of participants (3/20) experienced a relapse during the trial period compared with 11% (2/18) in the control group. We found no evidence of a difference in relapse between the two groups (odds ratio: 1.41; 95% CI: 0.21 to 9.58), but did not have sufficient power for an informative test.

For peer review only

Table 4. Summary statistics and unadjusted and adjusted treatment effects.

	Control (N = 13)	My Journey 3 (N = 20)	Unadjusted analysis		Analysis adjusted for baseline score	
**4-month scores**	Mean (SD)	Mean (SD)	Estimated difference	95% CI	Estimated difference	95% CI
**SIX** (Social Outcomes)	3.3 (1.9)	3.6 (1.3)	0.29	-0.84 to 1.43	0.16	-0.6 to 0.92
**MHCS** (Mental Health Confidence)	66.4 (12.7)	63 (15.8)	-3.43	-14.1 to 7.25	-4.81	-14.88 to 5.25
**QPR** (Recovery)						
Intrapersonal	47.8 (10.6)	43.2 (12.2)	-4.57	-13 to 3.87	-2.01	-8.43 to 4.49
Interpersonal	13.9 (2.4)	13.2 (2.3)	-0.72	-2.39 to 0.95	-0.42	-1.97 to 1.13
**MHCS** (Mental Health Confidence)	46.1 (9.9)	44 (11.3)	-2.08	-9.9 to 5.74	-0.19	-7.28 to 6.9
**DIALOG**						
Quality of life	4.4 (1.2)	4.5 (0.6)	0.07	-0.58 to 0.71	0.18	-0.38 to 0.74
Treatment satisfaction	5.4 (0.7)	5 (0.5)	-0.38	-0.83 to 0.06	-0.17	-0.6 to 0.25
**PANSS** (Symptom severity)						
**Positive**	9.3 (2.9)	11.4 (5.1)	2.09	-1.24 to 5.4	1.9	-0.49 to 4.3
**Negative**	10 (2.3)	11.1 (3.9)	1.05	-1.51 to 3.62	0.54	-1.6 to 2.67
**General**	23 (4)	24 (6.7)	1.21	-3.19 to 5.61	1.35	-2.68 to 5.37
**12-month scores**	Control (N = 14)	My Journey 3 (N = 16)	Unadjusted analysis		Analysis adjusted for baseline score	
	Mean (SD)	Mean (SD)	Estimated difference	95% CI	Estimated difference	95% CI
**SIX** (Social Outcomes)	3.2 (1.9)	3.5 (1.5)	0.29	-0.97 to 1.54	0.29	-0.73 to 1.3
**MHCS** (Mental Health Confidence)	66.2 (14.1)	71.1 (12.1)	4.81	-5 to 14.62	3.03	-6.04 to 12.1
**QPR** (Recovery)						
Intrapersonal	47.3 (11.5)	49.5 (11.1)	2.2	-6.25 to 10.7	3.21	-4.12 to 10.5
Interpersonal	13.6 (3.4)	15.1 (3.3)	1.44	-1.09 to 3.96	1.62	-0.89 to 4.12
**MHCS** (Mental Health Confidence)	45.6 (11.3)	49.3 (9.7)	3.61	-4.24 to 11.46	5.03	-1.67 to 11.7
**DIALOG**						
Quality of life	4.7 (0.9)	5 (0.7)	0.28	-0.31 to 0.87	0.24	-0.33 to 0.81
Treatment satisfaction	5.3 (1)	5.2 (1.2)	-0.12	-0.93 to 0.69	0.31	-0.42 to 1.04
**PANSS** (Symptom severity)						
Positive	9.5 (2.1)	10.2 (2.1)	0.69	-0.98 to 2.36	0.88	-0.62 to 2.38
Negative	10.2 (2.2)	10.9 (3.3)	0.77	-1.51 to 3.05	0.14	-1.56 to 1.84
General	23.5 (5.4)	22.1 (3.5)	-1.38	-4.82 to 2.07	-1	-4.57 to 2.55
**SES** (Engagement with services)	10 (6.2)	9.5 (8)	-0.4	-6.08 to 5.28	3.11	-1.57 to 7.79

Estimated differences and associated 95% Confidence Intervals from linear regression models with the control group as reference. Missing data: 4-month PANSS scores – one control group participant, one treatment group participant. 12-month PANSS scores – two control group

For peer review only

participants. Note: 12-month SES data available for 13 control group participants, and 14 treatment group participants.

DISCUSSION

The present study examined the feasibility of conducting a RCT of a supported self-management Smartphone app in EIP services. My Journey 3 aims to facilitate recovery and prevent relapse primarily via the digital delivery of previously developed paper-and-pen self-management tools. The trial indicates that recruitment and retention in a RCT evaluating My Journey 3 is feasible, and that My Journey 3 can be delivered in EIP services. The level of My Journey 3 use was relatively low across the trial period.

Building on from extensive preliminary work with NHS staff and service users, adults with lived experience of psychosis and experts in digital health we were able to successfully develop a self-management Smartphone app that can be used in EIP services. My Journey 3 appeared to be safe with no related serious adverse events reported. My Journey 3 was successfully delivered to all participants in the treatment group, however technical problems with the intervention caused significant delays in providing access. Prior to any future evaluations technical problems with My Journey 3 will need to be identified and fixed to ensure the intervention is implemented as intended.

My Journey 3 use varied considerably between participants, with only a small proportion of participants frequently engaging with the app after obtaining access to it. This raises questions about whether use was at a level where it is likely that useful self-management activities were taking place: certainly not enough time was spent regularly enough for participants to be engaging in detailed monitoring of symptoms and early warning signs, tracking medication and activities

For peer review only

and referring to crisis or recovery plans. Despite that, 40% of participants used My Journey 3 for a minimum of 30 minutes which could be an adequate amount of time for users to effectively monitor relapse signs and follow a crisis plan when needed. We have not found evidence on how regularly EIP service users make use of pen and paper self-management interventions delivered in routine settings, and this was not measured in our trial. Long-term engagement with My Journey 3 appears a challenge, but low levels of app use is a common phenomenon with market research showing that 62% of users stop using Smartphone apps after ten or fewer uses.[48]

Age has been shown to be an important factor linked to engagement with mental health apps and general Smartphone use,[49] and could partially explain differences in user engagement of My Journey 3. The treatment group however featured only a small number of participants from older age groups. We therefore lack informative data regarding app engagement for older participants and we are accordingly unable to explore if engagement and pattern of use of My Journey 3 varied between age groups. We will report separately on qualitative findings from this study exploring further the acceptability of My Journey 3 and drivers of engagement and non-adherence.

Participant retention for research data collection was high, with 75% of the sample attending the 12-month follow-up assessment, and is comparable to other Smartphone app studies.[50] Completion rates of the SES by EIP service clinicians were much lower at the 12-month follow in comparison to baseline, potentially due to staff changes and participants being discharged from services. Recruitment strategies were largely successful, however data is lacking on overall proportion of caseload recruited, reasons for non-inclusion and the numbers that were assessed for eligibility, thus limiting the conclusions we can make regarding trial feasibility.

For peer review only

The trial was not powered to detect effectiveness, and, as expected with our small number of participants, we found no significant differences between groups on any outcomes, with confidence intervals generally including substantial effects in either direction. Accordingly we cannot draw any conclusions regarding the potential impact of My Journey 3 as a mental health intervention. The proposed primary outcome for a full-scale trial, relapse as defined by use of an acute mental health service during the trial period, was marked by low event rates. Only five participants (12.5%) experienced a relapse during the one year follow-up period, compared with expected levels of 12% to 47%.[51] Consideration should be given to whether relapse, or our measure of relapse, is an appropriate outcome for a future RCT of this intervention. Symptom severity or alternatively patient-valued outcomes of personal recovery that self-management interventions have been shown to benefit may be more suitable primary outcomes in a future large scale trial.[12]

Strengths and limitations

My Journey 3 has been developed with extensive stakeholder input, and the intervention has been tested through lab-testing and a field study prior to the feasibility RCT. In comparison to previous studies,[50] participants had access to the app for a longer period of time. Participants' app use and usage data may be more reflective of real-world use as a result. Participant data were also collected from a wide range of methods including from participant assessments and patient records. The proposed primary outcome for a future RCT (relapse) was measured objectively and data were obtained for 95% of participants.

We recruited until the required number of participants was obtained rather than screening caseloads objectively: as a result we are not aware of the proportion eligible who were recruited, reasons for non-eligibility and how many EIP service users declined to take part and why. This

limits our understanding of how feasible conducting a large scale trial of this intervention would be. In addition there were issues with the usage data, which impacts the reliability of our conclusions regarding how often participants engaged with My Journey 3.

The trial did not feature an active digital placebo for the control group, meaning that non-specifics of Smartphone use could not be controlled for. Furthermore data was not collected during the study period from either group regarding frequency of completing recovery work such as relapse prevention plans, recovery plans or crisis plans either in paper-and-pen or digital format, limiting our understanding of whether access to My Journey 3 facilitated increased access to self-management activities.

Although clinicians were encouraged to support participants with My Journey 3, support was not manualised and clinicians did not have personal access to the app or associated data, potentially limiting the level and quality of the support offered and therefore user engagement. Future developments of My Journey 3 should focus on effective implementation and delivery within healthcare settings, and there should be considerations on how to facilitate secure data-sharing between My Journey 3 and healthcare records or other secure web-based platforms dependent on user consent, which is likely to increase clinician engagement with the app and its utility.[52]

We did also not define pre-specified criteria for assessing the feasibility of a RCT and the acceptability of My Journey 3. Instead we will consider all findings from the trial, app usage data and feedback from qualitative interviews yet to be reported in determining whether My Journey 3 will be evaluated in a full-scale trial. This allows all data from the RCT to be thoroughly considered, but may be a less objective approach in determining feasibility than using pre-defined criteria. Although the trial was not designed to assess intervention effectiveness, participants and

For peer review only

trial researchers were not blinded to group allocation, and as such could have led to an inflation of any observed effects.

Finally the sample consisted of Android Smartphone users who were generally stable and in an appropriate stage of recovery to consider using a self-management Smartphone app. Participants may therefore not be representative of all EIP service users. Furthermore contact with a researcher within a trial context could have led to increased intervention engagement that would not occur in a real-world clinical environment.

Conclusions

We developed and delivered a self-management Smartphone app for first-episode psychosis in a trial context. Participants were successfully recruited, most engaged at least to some extent with the intervention, and they had high follow-up rates over the one year trial period. Based on the data presented the trial methods appear feasible. My Journey 3 was shown to be safe, but the level of use was lower than anticipated thus potentially limiting its utility, although usage levels were higher than reported for downloaded apps in the general population.

If My Journey 3 is to be further tested in a research setting, attention needs to be given to engagement, a challenge associated with many digital tools in mental health.[53] Further usability testing in lab and field settings may also be a means to improving engagement. Other potential strategies including making more efforts to engage clinicians as well as service users with My Journey 3 by giving them access to the tool and to aspects of the planning and monitoring that service users conduct through it. The app could also potentially be offered as part of a blended approach to self-management, with pen and paper tools also used and as a whole service strategy for implementation of self-management. Refinements required before participating to a full trial

For peer review only

including participant and assessor blinding and manualised clinician support should be considered prior to conducting a future RCT.

For peer review only

Acknowledgements: The ARIES research team are grateful to their software collaborators MyOxygen for their technical development and hosting of My Journey 3 and to Ali Mousa for his valuable contribution to the development of the original My Journey app. We are grateful to Max Birchwood for his permission to incorporate 'Back in the Saddle' in to My Journey 3. We are grateful to Rachel Perkins for her permission to adapt the Personal Recovery Plan resource and incorporate in to My Journey 3.

Author Contributions: SJ is the Chief Investigator, based at University College London, DO the co-Chief Investigator, and TS the project manager. The trial design was developed by SJ, DO, BLE and PO. SA, HR, PO and ME have led on the development of the intervention. TS conducted the statistical analysis, with advice from RJ. TS wrote the draft of the paper, which was revised and approved by all authors. All authors approved the final manuscript.

Funding: The research is funded by the National Institute for Health Research (NIHR) Collaboration for Leadership in Applied Health Research and Care North Thames at Barts Health NHS Trust (NIHR CLAHRC North Thames). The views expressed in this article are those of the authors and not necessarily those of the NHS, the NIHR or the Department of Health and Social Care. SJ, DO and BLE are supported by the NIHR Mental Health Research Policy Unit, the NIHR Collaboration for Leadership in Applied Health Research and Care (CLAHRC) North Thames and the UCLH Biomedical Research Centre.

Data statement: The datasets generated during and/or analysed during the current study will be made available two years after the trial end.

Competing interests: None declared.

For peer review only

Legends:

Figure 1. Screenshots of the My Journey 3 app. a) The homescreen, b) the 'My goals' section of the recovery plan, c) the 'Coping with early warning signs' section of the relapse prevention plan, d) an example item from the Symptom Tracker, e) the Information section.

Figure 2. CONSORT diagram of the ARIES feasibility trial. Note: DNA, did not attend.

Figure 3. Bar chart displaying how long after the training session participants disengaged with My Journey 3.

For peer review only

REFERENCES

1 Bird V, Premkumar P, Kendall T, et al. Early intervention services, cognitive–behavioural therapy and family intervention in early psychosis: systematic review. *Brit J Psychiat*. 2010;197:350-6.

2 Tsiachristas A, Thomas T, Leal J, et al. Economic impact of early intervention in psychosis services: results from a longitudinal retrospective controlled study in England. *BMJ Open* 2016;6:e012611.

3 Alvarez-Jiménez M, Parker AG, Hetrick SE, et al. Preventing the second episode: a systematic review and meta-analysis of psychosocial and pharmacological trials in first-episode psychosis. *Schizophrenia Bull*. 2009;37:619-30.

4 Kam SM, Singh SP, Upthegrove R. What needs to follow early intervention? Predictors of relapse and functional recovery following first-episode psychosis. *Early Interv Psychia*. 2015;9:279-83.

5 Puntis S, Oke J, Lennox B. Discharge pathways and relapse following treatment from early intervention in psychosis services. *BJPsych open* 2018;4:368-74.

6 Leucht S, Heres S. Epidemiology, clinical consequences, and psychosocial treatment of nonadherence in schizophrenia. *J Clin Psychiatry* 2006;67:3-8.

7 Haddock G, Eisner E, Boone C, et al. An investigation of the implementation of NICE-recommended CBT interventions for people with schizophrenia. *J Ment Health* 2014:1;23:162-5.

8 Mueser KT, Meyer PS, Penn DL, et al. The Illness Management and Recovery program: rationale, development, and preliminary findings. *Schizophrenia Bull* 2006;32:S32-43.

For peer review only

9 Mueser KT, Corrigan PW, Hilton DW et al. Illness management and recovery: A review of the research. *Psychiat Serv* 2002;53:1272-84.

10 Cook JA, Copeland ME, Jonikas JA, et al. Results of a randomized controlled trial of mental illness self-management using Wellness Recovery Action Planning. *Schizophrenia Bull* 2011;38:881-91.

11 Färdig R, Lewander T, Melin L, et al. A randomized controlled trial of the illness management and recovery program for persons with schizophrenia. *Psychiat Serv* 2011;62:606-12.

12 Lean M, Fornells-Ambrojo M, Milton A, et al. Self-management interventions for people with severe mental illness: systematic review and meta-analysis. *Brit J Psychiat* 2019;22:1-9.

13 NICE Guidance - Psychosis and schizophrenia in adults: prevention and management. https://www.nice.org.uk/guidance/cg178/ifp/chapter/peer-support-and-self-management [accessed 2019-01-30]

14 Whitehead L, Seaton P. The effectiveness of self-management mobile phone and tablet apps in long-term condition management: a systematic review. *J Med Internet Res* 2016;18:e97.

15 Poushter J. 2016 Smartphone ownership and internet usage continues to climb in emerging economies. URL: http://www.pewglobal.org/2016/02/22/smartphone-ownership-and-internet-usage-continues-to-climb-in-emerging-economies/ [accessed 2019-01-30]

16 Wigginton C, Curran M, Broedeur C. 2017. Global mobile consumer trends, 2nd edition. URL: https://www2.deloitte.com/content/dam/Deloitte/us/Documents/technology-media-telecommunications/us-global-mobile-consumer-survey-second-edition.pdf. [accessed 2019-07-04]

For peer review only

17 Firth J, Cotter J, Torous J, et al. Mobile phone ownership and endorsement of "mHealth" among people with psychosis: a meta-analysis of cross-sectional studies. *Schizophrenia Bull* 2015;42:448-55.

18 Robotham D, Satkunanathan S, Doughty L, Wykes T. Do We Still Have a Digital Divide in Mental Health? A Five-Year Survey Follow-up. *J Med Internet Res* 2016;18:e309.

19 Naslund JA, Aschbrenner KA, Bartels SJ. How people with serious mental illness use smartphones, mobile apps, and social media. *Psychiatr Rehabil J* 2016;39:364-367.

20 Aref-Adib G, O'Hanlon P, Fullarton K, et al. A qualitative study of online mental health information seeking behaviour by those with psychosis. *BMC Psychiatry* 2016;16:232.

21 Berry N, Lobban F, Bucci S. A qualitative exploration of service user views about using digital health interventions for self-management in severe mental health problems. *BMC Psychiatry* 2019;19:35.

22 Proudfoot J. The future is in our hands: the role of mobile phones in the prevention and management of mental disorders. *Aust NZ J Psychiat* 2013;47:111-3.

23 Gottlieb JD, Romeo KH, Penn DL, et al. Web-based cognitive–behavioral therapy for auditory hallucinations in persons with psychosis: a pilot study. *Schizophr Res* 2013;145:82–87.

24 Granholm E, Ben-Zeev D, Link PC, et al. Mobile Assessment and Treatment for Schizophrenia (MATS): a pilot trial of an interactive text-messaging intervention for medication adherence, socialization, and auditory hallucinations. *Schizophr Bull* 2011;38:414-25.

25 Ben-Zeev D, Kaiser SM, Brenner CJ, et al. Development and usability testing of FOCUS: A smartphone system for self-management of schizophrenia. *Psychiatr Rehabil J* 2013;36:289-296.

For peer review only

26 Alvarez-Jimenez M, Bendall S, Lederman R, et al. On the HORYZON: moderated online social therapy for long-term recovery in first episode psychosis. *Schizophr Res* 2013;143:143-9.

27 Bonet L, Izquierdo C, Escartí MJ, et al. Use of mobile technologies in patients with psychosis: A systematic review. *Rev Psiquiatr Salud Ment* 2017;10:168-78.

28 Rus-Calafell M, Schneider S. Are we there yet?!—a literature review of recent digital technology advances for the treatment of early psychosis. *mHealth* 2019. doi: 10.21037/mhealth.2019.09.14

29 Camacho E, Levin L, Torous J. Smartphone Apps to Support Coordinated Specialty Care for Prodromal and Early Course Schizophrenia Disorders: Systematic Review. *J Med Internet Res* 2019;21:e16393.

30 Bucci S, Barrowclough C, Ainsworth J, et al. Actissist: Proof-of-Concept Trial of a Theory-Driven Digital Intervention for Psychosis. *Schizophrenia Bull* 2018; sby032.

31 JMIR. URL: https://support.jmir.org/hc/en-us/articles/115001389307-Does-my-trial-RCT-have-to-be-registered- [accessed 2019-10-08]

32 Steare T, O'Hanlon P, Eskinazi M, et al. App to support Recovery in Early Intervention Services (ARIES) study: protocol of a feasibility randomised controlled trial of a self-management Smartphone application for psychosis. *BMJ Open* 2019;9:e025823.

33 Eldridge SM, Chan CL, Campbell MJ, et al. CONSORT 2010 statement: extension to randomised pilot and feasibility trials. *BMJ* 2016;355:i5239.

34 National Institute for Health and Care Excellence. Implementing the Early Intervention in Psychosis Access and Waiting Time Standard: Guidance. London: NHS England Publications. 2016. p. 1-57.

For peer review only

35 Julious SA. Sample size of 12 per group rule of thumb for a pilot study. *Pharm Stat* 2005;287-91.

36 Plaistow J, Birchwood M. Back in the saddle: a guide to relapse prevention. In: Birchwood M, Fowler D, Jackson C, editors. Early Intervention in Psychosis: A Guide to Concepts, Evidence and Interventions. Chichester: McGraw-Hill; 1996. p. 239-244.

37 Perkins R, Rinaldi M. Taking back control: a guide to planning your own recovery. South West London: St George's Mental Health NHS Trust, 2007.

38 Priebe S, Watzke S, Hansson L, et al. Objective social outcomes index (SIX): a method to summarise objective indicators of social outcomes in mental health care. *Acta Psychiat Scand* 2008;118:57-63.

39 Carpinello SE, Knight EL, Markowitz FE, et al. The development of the Mental Health Confidence Scale: A measure of self-efficacy in individuals diagnosed with mental disorders. *Psychiatr Rehabil J* 2000;23:236-243.

40 Neil ST, Kilbride M, Pitt L, et al. The questionnaire about the process of recovery (QPR): a measurement tool developed in collaboration with service users. *Psychosis* 2009;1:145-55.

41 Tennant R, Hiller L, Fishwick R, et al. The Warwick-Edinburgh mental well-being scale (WEMWBS): development and UK validation. *Health Qual Life Out* 2007;5:63.

42 Priebe S, McCabe RO, Bullenkamp J, et al. Structured patient–clinician communication and 1-year outcome in community mental healthcare. *Brit J Psychiat* 2007;191:420-6.

43 Kay SR, Fiszbein A, Opfer LA. The positive and negative syndrome scale (PANSS) for schizophrenia. *Schizophrenia Bull* 1987;13:261-76.

For peer review only

44 Tait L, Birchwood M, Trower P. A new scale (SES) to measure engagement with community mental health services. *J Ment Health* 2002;11:191-8.

45 Johnson S, Lamb D, Marston L, et al. Peer-supported self-management for people discharged from a mental health crisis team: a randomised controlled trial. *Lancet* 2018;392:409-18.

46 Kirkbride JB, Fearon P, Morgan C, et al. Heterogeneity in incidence rates of schizophrenia and other psychotic syndromes: findings from the 3-center AeSOP study. Arch Gen Psychiat. 2006:63;250-8.

47 Adamson V, Barrass E, McConville S, et al. Implementing the access and waiting time standard for early intervention in psychosis in the United Kingdom: An evaluation of referrals and post-assessment outcomes over the first year of operation. *Early Interv Psychia* 2018:12;979-86.

48 Localytics. 21% of users abandon an app after one use. http://info.localytics.com/blog/21-percent-of-users-abandon-apps-after-one-use. [accessed 2019-05-16]

49 Beard C, Silverman AL, Forgeard M, Wilmer MT, Torous J, Björgvinsson T. Smartphone, social media, and mental health app use in an acute transdiagnostic psychiatric sample. *JMIR mHealth uHealth* 2019;7(:e13364.

50 Firth J, Torous J. Smartphone apps for schizophrenia: a systematic review. *JMIR Mhealth Uhealth* 2015;3.

51 Alvarez-Jimenez M, Priede A, Hetrick SE, et al. Risk factors for relapse following treatment for first episode psychosis: a systematic review and meta-analysis of longitudinal studies. *Schizophr Res* 2012;139:116-28.

For peer review only

52 Torous J, Woodyatt J, Keshavan M, Tully LM. A new hope for early psychosis care: the evolving landscape of digital care tools. *British J Psychiat* 2019;214:269-72.

53 Aref-Adib G, McCloud T, Ross J, et al. Factors affecting implementation of digital health interventions for people with psychosis or bipolar disorder, and their family and friends: a systematic review. *Lancet Psychiat* 2018;6:257-66.

For peer review only

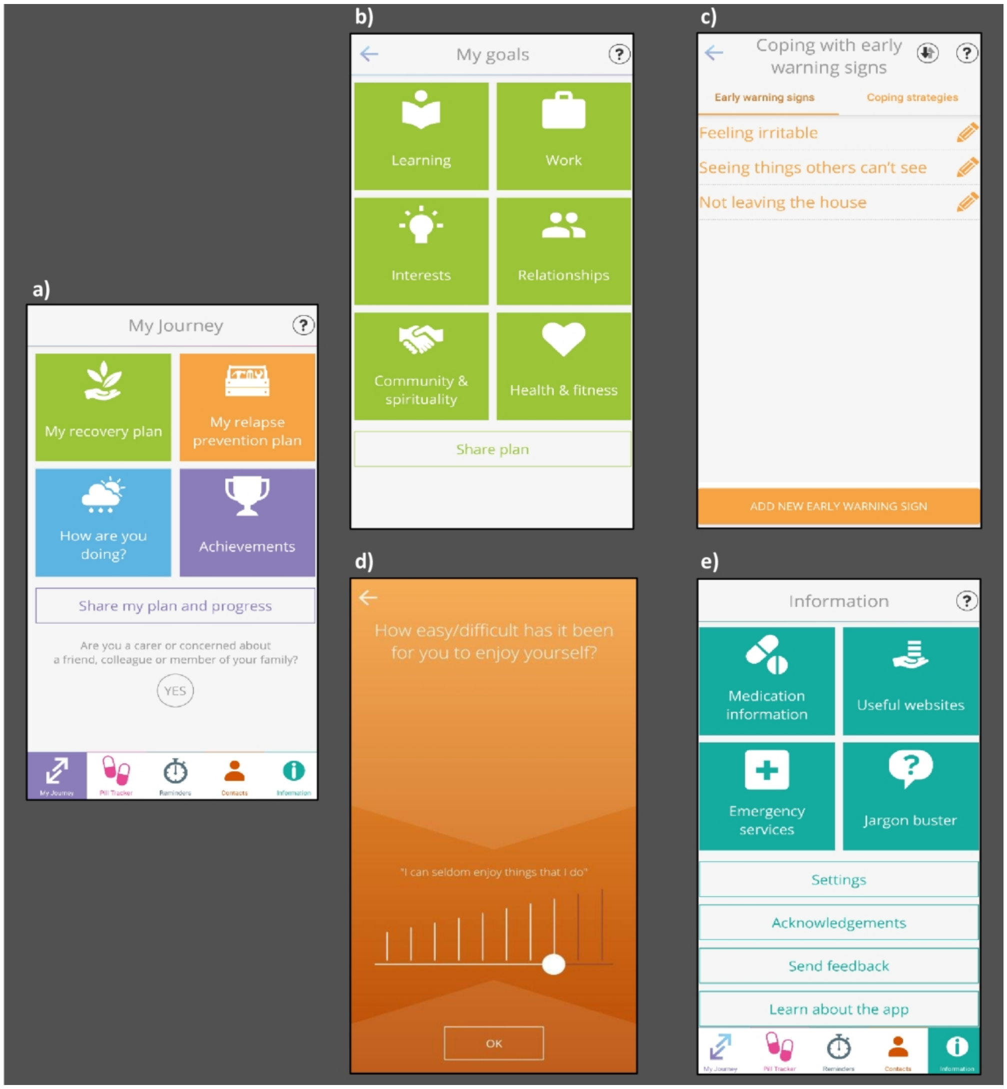

Figure 1. Screenshots of the My Journey 3 app. a) The homescreen, b) the 'My goals' section of the recovery plan, c) the 'Coping with early warning signs' section of the relapse prevention plan, d) an example item from the Symptom Tracker, e) the Information section.

169x182mm (300 x 300 DPI)

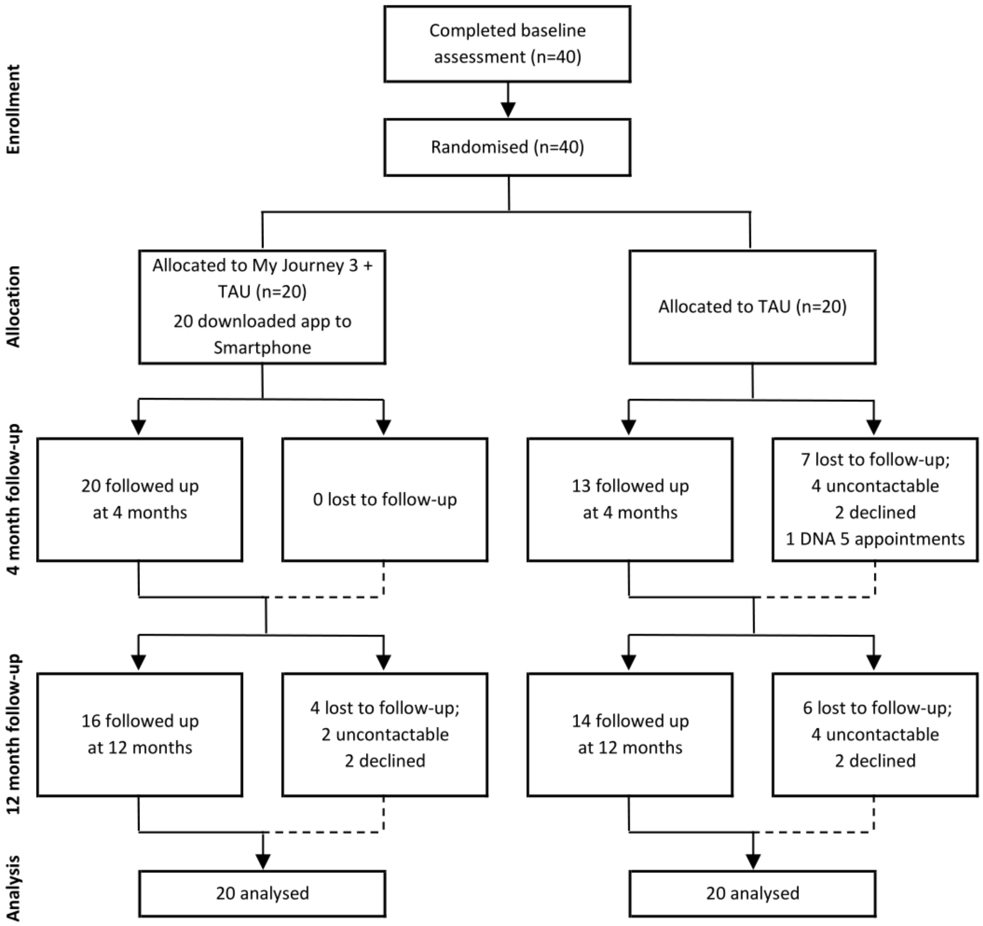

Figure 2. CONSORT diagram of the ARIES feasibility trial. Note: DNA, did not attend.

90x85mm (300 x 300 DPI)

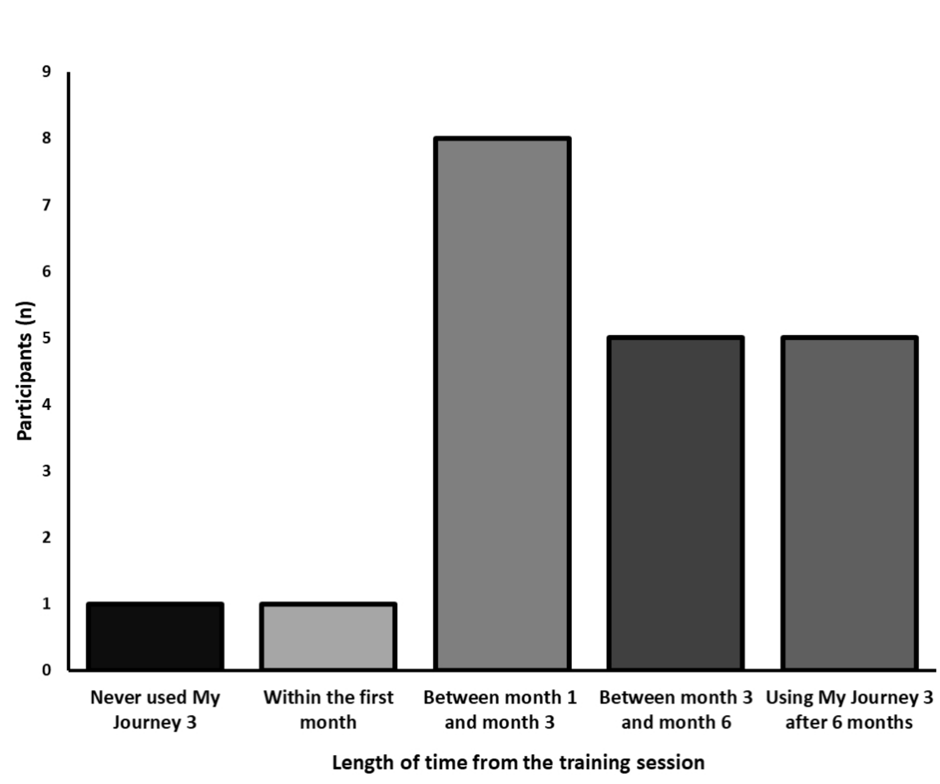

Figure 3. Bar chart displaying how long after the training session participants disengaged with My Journey 3.

101x79mm (300 x 300 DPI)

Service user participant consent form

Study Title: App to support Recovery In Early intervention Services (the ARIES study): Pilot randomised controlled trial of a self-management smartphone application

Principal Investigators: Professor Sonia Johnson and Professor David Osborn

Please Initial Each Box

1. I confirm that I have read and understood the Participant Information Sheet V5 dated 29/05/2017 for the above study and have had the opportunity to ask questions about the study. ☐
2. I understand that my participation is voluntary and that I am free to withhold personal information or to withdraw my participation at any time, without giving any reason, and without my medical care or legal rights being affected. ☐
3. I understand that if I choose to withdraw from the study that any data that I have already provided for the purposes of the research will be kept and used by the research team. ☐
4. I give permission for my General Practitioner (GP) and my Early Intervention team to be told I am participating in this study. ☐
5. I understand that relevant sections of my medical notes and data collected during the study may be looked at by regulatory authorities or from the NHS Trust, where it is relevant to my taking part in this research. I give permission for these individuals to have access to my records. ☐
6. I understand that I will be given a £20 gift as cash for my participation in each study assessment. ☐
7. I agree to the research team consulting NHS electronic records to investigate my diagnosis, medication, and mental health service use, and give them permission to do so even if I choose to no longer participate in the intervention, or they are not able to carry out further study interviews with me. ☐
8. I understand that in the event that I disclose information which may indicate new risk to myself or others, the researcher will be obliged to follow NHS Trust risk procedures that may require release of my personal data. ☐
9. I give permission for findings from the study to be written up for publication. Any publication will not identify me. ☐
10. I give permission to be audio recorded where required for the purposes of the study. I understand these audio-recordings will be transcribed and anonymised and audio recordings destroyed after the study. I give permission for direct quotations taken from this interview to be included in papers written for publication. Any quotation would not identify me. ☐
11. I give permission for the research team to collect data from the My Journey 3 app regarding the frequency, duration, and pattern of my use of it. I understand that no personal information will be collected from the app. ☐
12. I give permission for non-identifiable data to be shared with other research teams for research purposes. ☐

For peer review only - http://bmjopen.bmj.com/site/about/guidelines.xhtml

13. I agree to take part in this study. ☐

______________________ ______________ ______________________
Name of participant Date Signature

______________________ ______________ ______________________
Name of Researcher taking consent Date Signature

For peer review only

For peer review only - http://bmjopen.bmj.com/site/about/guidelines.xhtml

CONSORT 2010 checklist of information to include when reporting a pilot or feasibility trial*

Section/Topic	Item No	Checklist item	Reported on page No
**Title and abstract**			
	1a	Identification as a pilot or feasibility randomised trial in the title	Title Page
	1b	Structured summary of pilot trial design, methods, results, and conclusions (for specific guidance see CONSORT abstract extension for pilot trials)	Abstract
**Introduction**			
Background and objectives	2a	Scientific background and explanation of rationale for future definitive trial, and reasons for randomised pilot trial	Introduction
	2b	Specific objectives or research questions for pilot trial	Introduction
**Methods**			
Trial design	3a	Description of pilot trial design (such as parallel, factorial) including allocation ratio	Methods (design)
	3b	Important changes to methods after pilot trial commencement (such as eligibility criteria), with reasons	N/A
Participants	4a	Eligibility criteria for participants	Methods (participants)
	4b	Settings and locations where the data were collected	Methods (setting)
	4c	How participants were identified and consented	Methods (recruitment strategy)
Interventions	5	The interventions for each group with sufficient details to allow replication, including how and when they were actually administered	Methods (interventions)
Outcomes	6a	Completely defined prespecified assessments or measurements to address each pilot trial objective specified in 2b, including how and when they were assessed	Methods (outcomes)
	6b	Any changes to pilot trial assessments or measurements after the pilot trial commenced, with reasons	N/A
	6c	If applicable, prespecified criteria used to judge whether, or how, to proceed with future definitive trial	N/A
Sample size	7a	Rationale for numbers in the pilot trial	Methods (participants)

For peer review only

	7b	When applicable, explanation of any interim analyses and stopping guidelines	Methods (analysis)
Randomisation:			
Sequence generation	8a	Method used to generate the random allocation sequence	Methods (randomisation)
	8b	Type of randomisation(s); details of any restriction (such as blocking and block size)	Methods (randomisation)
Allocation concealment mechanism	9	Mechanism used to implement the random allocation sequence (such as sequentially numbered containers), describing any steps taken to conceal the sequence until interventions were assigned	Methods (randomisation)
Implementation	10	Who generated the random allocation sequence, who enrolled participants, and who assigned participants to interventions	Methods (randomisation)
Blinding	11a	If done, who was blinded after assignment to interventions (for example, participants, care providers, those assessing outcomes) and how	Methods (randomisation)
	11b	If relevant, description of the similarity of interventions	N/A
Statistical methods	12	Methods used to address each pilot trial objective whether qualitative or quantitative	Methods (analysis)
**Results**			
Participant flow (a diagram is strongly recommended)	13a	For each group, the numbers of participants who were approached and/or assessed for eligibility, randomly assigned, received intended treatment, and were assessed for each objective	Results (feasibility of trial design)
	13b	For each group, losses and exclusions after randomisation, together with reasons	Results (feasibility of trial design)
Recruitment	14a	Dates defining the periods of recruitment and follow-up	Results (feasibility of trial design)
	14b	Why the pilot trial ended or was stopped	N/A
Baseline data	15	A table showing baseline demographic and clinical characteristics for each group	Results (sample characteristics)
Numbers analysed	16	For each objective, number of participants (denominator) included in each analysis. If relevant, these numbers should be by randomised group	Results (participant outcomes)

For peer review only

Outcomes and estimation	17	For each objective, results including expressions of uncertainty (such as 95% confidence interval) for any estimates. If relevant, these results should be by randomised group	Results (participant outcomes)
Ancillary analyses	18	Results of any other analyses performed that could be used to inform the future definitive trial	N/A
Harms	19	All important harms or unintended effects in each group (for specific guidance see CONSORT for harms)	Results (participant outcomes)
	19a	If relevant, other important unintended consequences	N/A
**Discussion**			
Limitations	20	Pilot trial limitations, addressing sources of potential bias and remaining uncertainty about feasibility	Discussion (strength and limitations)
Generalisability	21	Generalisability (applicability) of pilot trial methods and findings to future definitive trial and other studies	Discussion (strength and limitations)
Interpretation	22	Interpretation consistent with pilot trial objectives and findings, balancing potential benefits and harms, and considering other relevant evidence	Discussion (conclusions)
	22a	Implications for progression from pilot to future definitive trial, including any proposed amendments	Discussion (conclusions)
**Other information**			
Registration	23	Registration number for pilot trial and name of trial registry	Abstract
Protocol	24	Where the pilot trial protocol can be accessed, if available	Additional file 3
Funding	25	Sources of funding and other support (such as supply of drugs), role of funders	Funding
	26	Ethical approval or approval by research review committee, confirmed with reference number	Methods (design)

Citation: Eldridge SM, Chan CL, Campbell MJ, Bond CM, Hopewell S, Thabane L, et al. CONSORT 2010 statement: extension to randomised pilot and feasibility trials. BMJ. 2016;355.
*We strongly recommend reading this statement in conjunction with the CONSORT 2010, extension to randomised pilot and feasibility trials, Explanation and Elaboration for important clarifications on all the items. If relevant, we also recommend reading CONSORT extensions for cluster randomised trials, non-inferiority and equivalence trials, non-pharmacological treatments, herbal interventions, and pragmatic trials. Additional extensions are forthcoming: for those and for up to date references relevant to this checklist, see www.consort-statement.org.

For peer review only

BMJ Open

Smartphone-delivered self-management for first-episode psychosis: the ARIES feasibility randomised controlled trial

Journal:	*BMJ Open*
Manuscript ID	bmjopen-2019-034927.R3
Article Type:	Original research
Date Submitted by the Author:	27-Jun-2020
Complete List of Authors:	Steare, Thomas; University College London, Division of Psychiatry<br>O'Hanlon, Puffin; University College London, Division of Psychiatry<br>Eskinazi, Michelle; University College London, Division of Psychiatry<br>Osborn, David; University College London, Division of Psychiatry; Camden and Islington NHS Foundation Trust<br>Lloyd-Evans, Brynmor ; University College London, Division of Psychiatry; Camden and Islington NHS Foundation Trust<br>Jones, Rebecca; University College London, Division of Psychiatry<br>Rostill, Helen; University of Surrey; Surrey and Borders Partnership NHS Foundation Trust<br>Amani, Sarah; Early Intervention in Psychosis Programme (South of England)<br>Johnson, Sonia; University College London, Division of Psychiatry; Camden and Islington NHS Foundation Trust
**Primary Subject Heading**:	Mental health
Secondary Subject Heading:	Health services research
Keywords:	Schizophrenia & psychotic disorders < PSYCHIATRY, MENTAL HEALTH, Clinical trials < THERAPEUTICS

SCHOLARONE™
Manuscripts

I, the Submitting Author has the right to grant and does grant on behalf of all authors of the Work (as defined in the below author licence), an exclusive licence and/or a non-exclusive licence for contributions from authors who are: i) UK Crown employees; ii) where BMJ has agreed a CC-BY licence shall apply, and/or iii) in accordance with the terms applicable for US Federal Government officers or employees acting as part of their official duties; on a worldwide, perpetual, irrevocable, royalty-free basis to BMJ Publishing Group Ltd ("BMJ") its licensees and where the relevant Journal is co-owned by BMJ to the co-owners of the Journal, to publish the Work in this journal and any other BMJ products and to exploit all rights, as set out in our licence.

The Submitting Author accepts and understands that any supply made under these terms is made by BMJ to the Submitting Author unless you are acting as an employee on behalf of your employer or a postgraduate student of an affiliated institution which is paying any applicable article publishing charge ("APC") for Open Access articles. Where the Submitting Author wishes to make the Work available on an Open Access basis (and intends to pay the relevant APC), the terms of reuse of such Open Access shall be governed by a Creative Commons licence – details of these licences and which Creative Commons licence will apply to this Work are set out in our licence referred to above.

Other than as permitted in any relevant BMJ Author's Self Archiving Policies, I confirm this Work has not been accepted for publication elsewhere, is not being considered for publication elsewhere and does not duplicate material already published. I confirm all authors consent to publication of this Work and authorise the granting of this licence.

For peer review only

TITLE PAGE

Title: Smartphone-delivered self-management for first-episode psychosis: the ARIES feasibility randomised controlled trial

Authors:

Thomas Steare

Division of Psychiatry, University College London, London, UK

Puffin O'Hanlon

Division of Psychiatry, University College London, London, UK

Michelle Eskinazi

Division of Psychiatry, University College London, London, UK

David Osborn

Division of Psychiatry, University College London, London, UK

R&D Department, Camden and Islington NHS Foundation Trust, London, UK

Brynmor Lloyd-Evans

Division of Psychiatry, University College London, London, UK

R&D Department, Camden and Islington NHS Foundation Trust, London, UK

Rebecca Jones

Division of Psychiatry, University College London, London, UK

Helen Rostill

University of Surrey, UK

Surrey and Borders Partnership NHS Foundation Trust, Leatherhead, Surrey, UK

Sarah Amani

EIP Programme (South of England), NHS England, Oxford, Oxfordshire, UK

For peer review only

Sonia Johnson (corresponding author)

Address: UCL Division of Psychiatry, 6th Floor, Wing B, Maple House, 149 Tottenham Court Road, London W1T 7NF

Division of Psychiatry, University College London, London, UK

R&D Department, Camden and Islington NHS Foundation Trust, London, UK

s.johnson@ucl.ac.uk

02076799453

Word count: 6,098

Keywords: Psychosis, Self-management, Smartphone, Feasibility study, Randomised controlled trial

For peer review only

ABSTRACT

Objectives: To test the feasibility and acceptability of a randomised controlled trial (RCT) to evaluate a Smartphone-based self-management tool in Early Intervention in Psychosis (EIP) services.

Design: A two-arm unblinded feasibility RCT.

Setting: Six NHS EIP services in England.

Participants: Adults using EIP services that own an Android Smartphone. Participants were recruited until the recruitment target was met (n=40).

Interventions: Participants were randomised with a 1:1 allocation to one of two conditions: (1) treatment as usual from EIP services (TAU) or (2) TAU plus access to My Journey 3 on their own Smartphone. My Journey 3 features a range of self-management components including access to digital recovery and relapse prevention plans, medication tracking and symptom monitoring. My Journey 3 use was at the users' discretion, and was supported by EIP service clinicians. Participants had access for a median of 38.1 weeks.

Primary and secondary outcome measures: Feasibility outcomes included recruitment, follow-up rates and intervention engagement. Participant data on mental health outcomes were collected from clinical records and from research assessments at baseline, 4 months and 12 months.

Results: 83% and 75% of participants were retained in the trial at the 4- and 12-month assessments. All treatment group participants had access to My Journey 3 during the trial, but technical difficulties caused delays in ensuring timely access to the intervention. The median

For peer review only

number of My Journey 3 uses was 16.5 (IQR 8.5 to 23) and median total minutes spent using My Journey 3 was 26.8 (IQR 18.3 to 57.3). No serious adverse events were reported.

Conclusions: Recruitment and retention were feasible. Within a trial context My Journey 3 could be successfully delivered to adults using EIP services, but with relatively low usage rates. Further evaluation of the intervention in a larger trial may be warranted, but should include attention to implementation.

Trial Registration: ISRCTN10004994

ARTICLE SUMMARY

Strengths and limitations of this study:

- Participant data was collected from a wide range of sources including questionnaires, patient records and from the app
- Participants were followed up for a 12-month period; longer than the majority of feasibility trials investigating Smartphone apps for psychosis
- We were not able to blind researchers or participants to their treatment allocation
- The study recruited users of Early Intervention in Psychosis services that own an Android Smartphone, limiting sample representativeness
- This is a feasibility study, and therefore does not have the statistical power to conclude the effectiveness of the intervention.

For peer review only

INTRODUCTION

Early Intervention in Psychosis (EIP) services have been established across the United Kingdom to provide care to adults during the three years following an initial episode of psychosis. There is evidence that such services are effective and cost-effective,[1,2] resulting in improvement in a range of outcomes yet challenges remain. Relapse rates for EIP service users are high [3] particularly after discharge [4,5] and limited adherence with antipsychotic medication is common [6]. There are also difficulties accessing psychosocial interventions,[7] including supported self-management.

Illness self-management is an approach designed to support people to manage long-term health conditions by developing their ability to recognise and monitor symptoms and early warning signs of relapse, identify and avoid stressors, make plans for achieving their own recovery and to effectively use coping strategies.[8] For people with psychosis, self-management tools have been shown to reduce psychological distress, improve medication adherence and reduce the likelihood of future hospital admissions.[9-11] In a recent meta-analysis, self-management interventions for severe mental illness were also found to have a significant benefit on patient-valued outcomes of personal recovery, hope and self-efficacy.[12] Despite clinician-supported self-management programmes being mandated in current UK treatment guidelines for first-episode psychosis,[13] there is a lack of well-evaluated tools to support delivery within EIP services. There is a clear need to overcome implementation barriers affecting the delivery of self-management to those likely to benefit from it.[12] A potentially convenient and economical way of achieving this is via the use of digital technology such as Smartphones.[14]

Smartphones can run advanced software known as apps that hold promise as an effective tool to assist the monitoring and treatment of mental health problems. Smartphone ownership is

For peer review only

rapidly growing world-wide [15] with a significant number of developed countries with ownership rates of more than 80%.[16] Adults with severe mental health problems have comparable Smartphone ownership rates to the general population,[17-19] and there is a growing consensus that adults with psychosis are open to using Smartphones to access mental health interventions.[20,21] Smartphones also provide high accessibility to the internet and are commonly carried on the person, meaning apps can be easily accessed at times and locations convenient for the user. Accordingly Smartphones have the capacity to deliver time-unlimited mental health interventions, such as self-management tools, and ultimately the potential to increase access to effective care and reduce healthcare costs.[22] The benefits of Smartphone apps may also extend beyond the original treatment period with a community team, and could be a valuable tool following discharge where the risk of relapse is increased.[4,5]

The majority of digital health interventions that have been developed for psychosis have been based on existing psychological therapies such as Cognitive Behavioural Therapy,[23,24] or other evidence-based interventions,[25,26] yet very little is known regarding their effectiveness when delivered in EIP services. A growing number of self-management apps for psychosis have been tested for feasibility and acceptability, including those delivered independent of a clinical setting and those embedded within clinical care.[27-29] These have shown promising levels of adoption and use in research contexts yet little is known about their clinical efficacy.

To date only one trial of a self-management app delivered in EIP services has published results regarding the interventions impact on clinical outcomes.[30] In the proof-of-concept trial an active self-management app "Actissist" was found to confer benefits over a passive control app. The study suggests that participants that received Actissist had better outcomes regarding their mood and general and negative symptoms post-treatment in comparison to control participants.

For peer review only - http://bmjopen.bmj.com/site/about/guidelines.xhtml

Actissist features a range of components including self-assessment questions focused on cognitive appraisals, emotions, behaviours and belief convictions and suggests appropriate coping strategies, but does not feature some major cornerstones of self-management such as relapse and recovery plans. Regardless results from this study suggest that such digital self-management interventions could potentially improve outcomes of people using EIP services. Further trials are needed before firm conclusions can be made regarding the feasibility of conducting RCTs in this field and of the therapeutic benefits of self-management apps for first-episode psychosis delivered in clinical settings.

We aimed to address this evidence gap by conducting a feasibility RCT of a supported self-management Smartphone app, "My Journey 3" designed to help EIP service users recognise early warning signs of illness, recognise and monitor symptoms and create plans for their recovery. My Journey 3 has been designed to be initially set up in EIP services and used with clinician support, but to also be suitable for independent use. The results of the feasibility RCT are a potential step towards a full-scale trial to assess the effectiveness of the intervention.

The objectives of this study were as follows:

1. To determine the acceptability of the My Journey 3 self-management app for use in an EIP service context
2. To determine the feasibility of trial procedures for a definitive trial, including recruitment, intervention enrolment and trial attrition.
3. To test procedures for evaluating intervention engagement and participant outcomes.

For peer review only

METHODS

Design

The App to support Recovery in Early Intervention Services (ARIES) study was an unblinded feasibility RCT with a nested qualitative study comparing a supported self-management Smartphone app (My Journey 3) in addition to Treatment As Usual (TAU), with a control group receiving TAU only. Participants were randomly allocated to one of the two trial arms in a 1:1 ratio. Since this was a feasibility trial, it was not designed to have sufficient statistical power to assess the effectiveness of the My Journey 3 intervention.

Ethical approval was obtained from the London Brent National Research Ethics Service Committee (Ref 15/LO/1453). The trial was retrospectively registered (ISRCTN10004994). As the study was a feasibility trial prospective registration was not required.[31] Further details of the methodology are available in the protocol paper.[32] We have followed the Consolidated Standards of Reporting Trials (CONSORT) statement extension for pilot and feasibility randomised trials for reporting.[33] A copy of the CONSORT checklist is provided as Additional file 1.

Setting

The trial was conducted in six EIP services across three NHS Foundation Trusts in England. EIP services are multi-disciplinary community mental health services that provide care coordination to people in the first three years of a first-episode psychosis, focusing on engagement, achieving social and clinical recovery and delivering a full range of pharmacological, psychological and social interventions.[34] The six EIP services as mandated in England provide care for people up to the age of 65, with the potential for adults above the age range to access EIP services if clinically appropriate although these cases are rare . Two of the participating Trusts are

For peer review only

located in inner London. The third Trust is located in a county outside of London with both urban and rural areas. Assessments were conducted face-to-face at EIP services, at participants' homes or at University College London.

Participants

Participants were recruited from the participating EIP services over seven months. We assumed a conservative 40% attrition rate and accordingly set the target sample size as 40 participants to ensure the trial retained twelve completer participants per group (as recommended to assess trial feasibility).[35] Participants were eligible if they were aged ≥16 years, had experienced at least one episode of psychosis, were currently on the caseload of an EIP service and owned a Smartphone with an Android operating system. People were excluded from the trial if they lacked capacity to consent to participation, were unable to communicate and understand English, or were considered by their EIP service to pose a high risk to researchers during meetings, even on NHS premises. Familiarity and competence in using digital technology or Smartphones was not an eligibility criterion.

Recruitment strategy

Clinicians at the participating EIP services were briefed by the research team, and were asked to make initial contact with eligible EIP service users. Clinicians explained the trial to service users, and enquired whether the service user would be willing to speak to a researcher about participating in the trial. The researcher then made contact with eligible and potentially willing service users, and arranged a face-to-face meeting where the trial was explained further. The researcher provided the trial information sheet (Additional file 2), and assessed the participant's capacity to provide informed consent. Service users had at least 24 hours after

For peer review only

receiving the information sheet to consider their participation. Participants then gave written informed consent to take part, prior to completing the baseline assessment. No participants were recruited via online methods.

Randomisation

Following the baseline assessment, participants were randomly allocated in a 1:1 ratio to either the intervention (n=20) or the control group (n=20) by an independent statistician. The treatment group had access to My Journey 3 in addition to TAU, whilst the control group received TAU only. An independent researcher held the allocation list and did not disclose participants' allocation to the trial researcher until after completion of the baseline assessments, allowing the researcher to remain blinded during recruitment and whilst carrying out the baseline assessments.

Due to the nature of the intervention, participants were not blinded to their group allocation. During the recruitment process, participants would have been aware that My Journey 3 was the intervention of interest. As a single researcher carried out the majority of data collection, it was not practical for the allocation of participants to be concealed from the research team. Participants were informed of their allocation by the researcher via a telephone call.

Interventions

My Journey 3

My Journey 3 is a Smartphone app developed for adults accessing EIP services. The aim of the intervention is to develop users' self-management skills to help them to achieve self-determined recovery goals and avoid future relapses. My Journey 3 is suitable for independent use, but also designed to be used with support from EIP service clinicians who will be able to assist with the completion of the self-management components and initial set-up. It is the developers'

For peer review only

aspiration for My Journey 3 to be used initially in collaboration with EIP service clinicians, and for it to support continuing self-management after users have been discharged from EIP services.

The development of My Journey 3 has been through several iterations. The first version (My Journey 1) was created by Surrey and Borders Partnership NHS Foundation Trust with leadership from Sarah Amani, for EIP service users to track their symptoms, set reminders for appointments and share their progress with EIP service clinicians. In developing the current version of My Journey 3 we have drawn on existing paper-and-pen self-management intervention components [36,37] to allow users to track recovery goals and personalise relapse prevention plans – important cornerstones of illness self-management. The design and the content of My Journey 3 was led by a collaboration of researchers, digital health experts, EIP service clinicians and service users. A private app development company based in the UK (MyOxygen; https://myoxygen.uk) led the technical development of My Journey 3. To limit costs My Journey 3 is only compatible with Smartphones with Android operating systems at this stage of testing.

My Journey 3 features four key elements of self-management, an approach with demonstrated efficacy in improving social and clinical outcomes for people with psychosis.[12] Screenshots of the key components are displayed in figure 1. Users have the ability to create a relapse prevention plan, where there is the opportunity to identify and list triggers, early warning signs of relapse and personalised coping strategies to refer to as required and to create a plan to follow if experiencing a crisis. Via the 'My Recovery Plan' section users are able to set recovery goals, list actions they can do to encourage well-being, and set reminders on their Smartphone to encourage engagement in these activities. Users can also use a tracker to monitor and rate their symptoms and early warning signs over time. In the Symptom Tracker users are presented with seventeen different symptoms and behaviours and are asked to respond via a "Yes/No" format as

For peer review only

to whether they have recently experienced these. Users that respond with a "Yes" are then presented with a 10-point scale (4-point scale for the early warning sign tracker) to rate the severity or frequency of the associated symptoms, with advice on how to manage these symptoms displayed. Psycho-education on mental health, medication and mental health services is provided in an 'Information' section. To encourage adherence with medication, users are encouraged to log and track their medication in the 'Pill Tracker' section. Users are able to set daily alerts to remind them to log whether they have taken their medication. My Journey 3 also features weekly discrete notifications to encourage engagement with the app, which can be disabled at the users' preference. The key components of My Journey 3 are summarized in Table 1, with further details available in the protocol paper.[32]

Prior to the feasibility trial reported in this paper, My Journey 3 was tested by EIP service users in lab-based usability tests and in a one-month field study. The final content of My Journey 3 was then refined based on feedback from individual interviews with the participating EIP service users and clinicians. No changes were made to the content of My Journey 3 during the feasibility RCT. A major technical update to My Journey 3 was carried out in January 2018 to fix compatibility issues with older versions of Android operating systems. This did not require any changes to the trial design.

For peer review only

Table 1. Key sections of the My Journey 3 Smartphone app.

Section	Features	Purpose
My recovery plan	Things I can do to keep well<br><br>My goals	To encourage users to have regular routines, track activities, set reminders and plan how to achieve long-term goals
My relapse prevention plan	Coping with triggers<br><br>Coping with early warning signs<br><br>Coping with a crisis<br><br>Crisis contacts	To help users' identify, monitor and cope with triggers and early warning signs<br><br>To help users create a "relapse plan" to follow in times of crisis
How are you doing?	My mood<br><br>My early warning signs<br><br>My tracker	For users to monitor symptoms, behaviours and early warning signs and track these experiences over time
Pill tracker		To log whether users' have taken their medication each day
Information	Medication information<br><br>Useful websites<br><br>Emergency services<br><br>Jargon buster	To provide users with useful information and external links on medication and mental health<br><br>To identify local emergency services in a time of crisis<br><br>To provide a glossary of terms that are commonly used in mental health care

Delivery

Following assignment to the treatment group, participants engaged in individual training sessions with a trial researcher and a supporting EIP service clinician. Training sessions were intended to take place within six weeks of the participants' initial baseline assessment, and lasted

For peer review only

for approximately 2 hours. During these sessions the researcher downloaded My Journey 3 onto the participants' Smartphone and gave a demonstration of the app and its main functions. Participants were then encouraged to input appropriate information to specific sections of My Journey 3 with the help of the supporting EIP service clinician in attendance. Following this session it was hoped that all participants had initial personal recovery plans, relapse prevention plans and crisis plans stored on My Journey 3.

Participants had access to My Journey 3 on their own Smartphone from the training session till the 12-month time-point. Researchers recommended that participants used My Journey 3 at least once a week, but participants had a free choice in how and when they used My Journey 3. Participants did not receive any financial incentives to use My Journey 3, and were free to withdraw from using the app or decline the installation of it on to their Smartphone. At the training session participants were informed by the researcher that My Journey 3 would be not suitable for seeking urgent medical care whilst in crisis, and that it is not a substitute for human support.

To encourage user engagement with My Journey 3 during the trial, supporting EIP service clinicians were asked to provide regular support and encouragement to service users who had access to My Journey 3. Clinicians were asked to discuss recovery goals and relapse prevention plans in routine appointments with participants, and assist with entering these into the appropriate My Journey 3 sections. Clinicians had an existing understanding of self-management approaches from their clinical training and practice, and would be able to provide appropriate advice with the intervention components of My Journey 3 but they received no formal training on how to implement My Journey 3 into their clinical work. Clinicians' understanding of operating My Journey 3 was from the training sessions only. Clinician support for My Journey 3 as part of the trial was not manualised or incentivised.

For peer review only

Participants were encouraged to contact the trial researcher in the case of technical problems with My Journey 3. The researcher contacted participants a week after the training session to check that My Journey 3 had been functioning without issues, and invited any questions about the app. No further prompts were instigated by the researcher during the trial.

Treatment as usual

All participants received TAU regardless of group allocation. TAU for a person under the care of EIP services typically involves regular meetings with a care co-ordinator, access to a psychiatrist, psychiatric medication, and a range of psychological interventions. EIP services are encouraged to deliver self-management programmes, that includes advice on symptom management, crisis planning and relapse prevention, generally delivered with paper-and-pen tools if at all.[34] None of the participating EIP services offered digital interventions or Smartphone apps as part of routine care during the study period, and structured self-management support, including the relapse prevention work recommended in EIP contexts, is inconsistently implemented.

Patient and Participant Involvement

The development of My Journey 3 has been guided by the input of people with lived experience of psychosis. Initial development of the design and content involved a collaboration between researchers, experts in digital health and service users. Service users provided further input into the design and functionality of My Journey 3 from providing feedback after taking part in lab-based tests and a field study.

For peer review only

Outcomes

Participant data were collected from numerous sources including participant assessments, patient records and anonymous My Journey 3 usage reports. There were no pre-specified criteria for assessing trial feasibility and intervention acceptability.

Questionnaire measures

Proposed outcome measures for a future trial were assessed at structured face-to-face assessments with a trained researcher at three time points; baseline, 4-months post baseline and 12-months post baseline. At all meetings participants completed self-report questionnaires that have been previously used with people with first-episode psychosis. Participants were given £20 as a thank you for completing the assessment at each time point.

At each assessment we collected sociodemographic data including age, gender, ethnicity, accommodation and living situation, employment status, educational attainment, Smartphone use, and use of other mental health apps. The following self-report measures were also collected: social outcomes (Social Outcomes Index (SIX),[38] score 0-6: higher score= better social outcomes), self-efficacy (Mental Health Confidence Scale (MHCS),[39] score 16-96: higher score= greater empowerment), self-rated recovery (Questionnaire about the Process of Recovery (QPR),[40] intrapersonal score 0-68, interpersonal score 0-20: higher score= greater recovery), mental well-being (Warwick-Edinburgh Mental Well-Being Scale (WEMWBS),[41] score 14-70: higher score= greater well-being) and quality of life and satisfaction with treatment (DIALOG scale,[42] score 1-7: higher score= greater quality of life/satisfaction with treatment).

For peer review only

Clinical structured interviews were also conducted with each participant by the researcher, to assess psychopathology, using the Positive and Negative Syndrome Scale (PANSS).[43] Higher PANSS scores are indicative of greater severity of each symptom domain.

Participants' engagement with EIP services were measured using the Service Engagement Scale (SES),[44] completed by EIP service clinicians known to each participant, typically care co-coordinators. Clinicians completed the SES at baseline and 12 months later, regardless of whether participants attended the 12-month assessment. Higher SES scores are indicative of poorer user engagement with EIP services.

Patient records

Clinical data were extracted from patient records at baseline and at the 12-month time point. Clinical measures included most recent diagnosis and use of EIP services, other community mental health teams and acute mental health services in the previous 12 months.

The proposed primary outcome for a future RCT (relapse of psychosis) was operationalised as an admission to an acute mental health service (inpatient psychiatric ward, crisis house, crisis resolution team or acute day care service) during the 12-month trial period as indicated in patient records. This definition of relapse has been used previously in a recent trial of a self-management intervention.[45]

My Journey 3 use

To assess acceptability of the intervention and user engagement, My Journey 3 usage data were collected for all participants in the treatment group from the training session until the 12-month time-point. Whenever users had Wi-Fi internet access on their Smartphone My Journey 3 automatically uploaded encrypted usage data to a secure server. Data collected included a record

For peer review only

of each time the user opened My Journey 3, whether this was in response to a prompt, and which components they used. To ensure confidentiality, personal or identifiable data such as text or responses to each sections were not collected.

Acceptability

Feedback was obtained through semi-structured interviews as part of a nested qualitative study. Individual interviews were conducted at the 4-month time-point with both service user participants that received My Journey 3 and supporting clinical staff.

Analysis

Participant demographic and clinical characteristics, My Journey 3 usage, and rates of participant recruitment and retention were summarised using descriptive statistics. As this was a feasibility RCT, it was not powered to assess the effectiveness of the intervention. Statistical analyses of participant outcome measures were conducted to pilot the methods of analysis for a fully powered effectiveness trial. Logistic regression was used to explore the impact of the My Journey 3 intervention on relapse. Linear regression was used to examine the potential effect of the intervention on continuous outcome measures at 4 months and 12 months separately. We report the effect estimates and corresponding 95% confidence intervals (CI) only for unadjusted analyses and for analyses adjusting for the baseline measure of the outcome in question. All analyses were performed using STATA V.14 after completion of the final participant assessment. No interim analyses were conducted.

Qualitative data was coded to themes based on the Acceptability of Healthcare Interventions framework.[46] Results of the nested qualitative study exploring the acceptability of

For peer review only

My Journey 3 and drivers of engagement and non-adherence will be reported in full elsewhere. Here we provide a short summary of findings.

RESULTS

Feasibility of trial design

Participant flow is detailed in the CONSORT diagram (figure 2). A total of 40 participants was recruited and randomised (20 to My Journey 3, 20 to TAU) over a 7-month period from March 2017 to September 2017. Participants were recruited until the required number of 40 was obtained: we do not therefore have a full assessment of the proportion of the teams' caseload who could have been recruited to a full trail, nor do we know the proportion of approached EIP services users that did not meet eligibility criteria or declined involvement in the trial.

Among those recruited to the trial, attrition rates were generally low: 83% (33/40) and 75% (30/40) of participants successfully attended and completed follow-ups at 4 months and 12 months respectively. At both time points the follow-up rate was lower in the control group (4-months: 65% compared to 100%, 12-months: 70% compared to 80%). Patient record data were available for all participants at baseline and for 95% of the sample (38/40) at the 12-month time-point. Completion rates of the SES by clinicians were higher at baseline (90%) than at the 12-month time-point (67.5%). Follow-up assessments were conducted from July 2017 to October 2018.

All participants in the treatment group attended a training session with a researcher, and had access to My Journey 3 during the trial. Issues with Smartphone compatibility initially prevented three participants from downloading My Journey 3. Following an update to the system two of the participants were able to install and access My Journey 3 on their own Smartphones. Two participants were provided with Smartphones with My Journey 3 pre-installed (the app was

For peer review only

still incompatible on one participant's Smartphone despite the update; another participant no longer owned an Android Smartphone after entering the trial). The median length of time from trial enrolment to having access to My Journey 3 was 14 weeks (IQR 11 to 17), longer than the planned time of 6 weeks. Participants had access to My Journey 3 for a median of 38.1 weeks (IQR 34.8 to 40.7). There were no reported privacy breaches.

My Journey 3 usage data were collected for all participants following the training session, with 500 different data entries available for analysis. Within the 500 data entries, 27 (5.4%) were corrupt and were subsequently removed from the analysis. The unusable data can grouped into two types. The first, duplicates of previous data entries that were subsequently removed. The second, entries where the times were implausible (for example, the end time of using My Journey 3 was recorded as occurring before the start time). In addition, a further issue caused errors with accurately recording My Journey 3 usage data of 'My Recovery Plan' and 'My Relapse Plan' sections. As a result we were unable to accurately conclude how often participants used these sections.

One participant randomised to the control group was wrongly given access to My Journey 3. For the purpose of the statistical analysis they are classed as a control participant.

Sample characteristics

A summary of demographic and clinical characteristics of the sample is displayed in Table 2. The sample was predominantly male (n=28, 70%). The mean age of the sample was 29.7 years (SD = 9.78) and similar to that of UK cohorts of EIP service users at first presentation.[47,48] Six participants were over the age of 35, with these participants spread evenly across the two groups. Most participants had a diagnosis of a schizophrenia, schizotypal or delusional disorder (ICD code

F20-F29) and were not in paid employment. A quarter of the sample (n=10, 25%) had completed a university degree. Eight (20%) participants had previously used a mental health app.

Table 2. Key demographic and clinical characteristics of the sample at baseline.

	Control (n=20)	My Journey 3 (n=20)
**Age (years) – mean (SD), [min, max]**	30 (10.1), [18.8, 64.7]	29.4 (9.7), [17.6, 52.4]
**Gender**		
Female	7 (35%)	5 (25%)
**Ethnicity**		
White British	6 (30%)	8 (40%)
Any other white/Mixed white	2 (10%)	1 (5%)
Black African	5 (25%)	3 (15%)
Black Caribbean	1 (5%)	1 (5%)
Black Other	1 (5%)	0
Asian Indian	1 (5%)	0
Asian Other	1 (5%)	2 (10%)
Other/Mixed other	3 (15%)	3 (15%)
**Education**		
Undergraduate degree	6 (30%)	4 (20%)
Some University but no degree	3 (15%)	2 (10%)
Higher National Degree or professional qualification	2 (10%)	1 (5%)
A Levels or equivalent	3 (15%)	4 (20%)
GCSEs or equivalent	4 (20%)	6 (30%)
No qualifications	1 (5%)	3 (15%)
Missing	1 (5%)	0
**Employment status**		
Employed – more than 16 hours a week	4 (20%)	4 (20%)
Employed – less than 16 hours a week	0	2 (10%)
Voluntary work	3 (15%)	3 (15%)
In study or training	1 (5%)	1 (5%)
Unemployed or exempt due to disability	8 (40%)	8 (40%)
Missing	4 (20%)	2 (10%)
**Primary diagnosis (ICD-10 code)**		
F10-F19: Mental and behavioural disorder due to psychoactive substance use	1 (5%)	0
F20-F29: Schizophrenia, schizotypal and delusional disorder	16 (80%)	13 (65%)
F30-F39: Mood disorder	1 (5%)	5 (25%)
Missing	2 (10%)	2 (10%)
**Admission to an acute mental health service in previous year**		
Yes	11 (55%)	10 (50%)
**SIX – mean (SD), [min, max]**	3.2 (1.5), [0, 6]	3.6 (1.5), [1, 6]
**MHCS – mean (SD), [min, max]**	59.7 (17.8), [16, 82]	61.2 (12.6), [38, 78]
**QPR – mean (SD), [min, max]**		
Intrapersonal	45.7 (12), [22, 68]	42.2 (10.6), [24, 60]
Interpersonal	13.7 (2.7), [9, 19]	12.9 (3.4), [5, 19]
**WEMWBS – mean (SD), [min, max]**	43.4 (11.6), [25, 69]	40.3 (10.2), [23, 57]
**DIALOG – mean (SD), [min, max]**		
Quality of life	4.5 (1), [2.8, 6.5]	4.4 (0.8), [3, 5.7]
Treatment satisfaction	5.4 (0.7), [4.3, 7]	4.8 (0.7), [3.7, 6]

For peer review only

**PANSS – mean (SD), [min, max]**		
Positive	10.9 (5), [7, 22]	11.3 (4.2), [7, 19]
Negative	10.7 (2.5), [7, 19]	11.8 (4.5), [7, 20]
General	26.6 (6), [17, 39]	26.2 (8), [16, 46]
**SES – mean (SD), [min, max]**	11.3 (7.9), [0, 26]	9.6 (7), [0, 23]

All statistics are reported N (%) unless otherwise specified. Missing data: PANSS scores – one control group participant, SES – three control group participants, one treatment group participant.

My Journey 3 use

The level of My Journey 3 use was highly skewed. The median number of times My Journey 3 was used per participant during the trial was 16.5 (IQR 8.5 to 23). Participants accessed My Journey 3 a median of 3.22% (IQR 1.89 to 6.36) of the days it was available to them, equating to My Journey 3 being used on average once every 31 days (IQR 15.7 to 52.9). Participants spent a median of 26.8 minutes (IQR 18.3 to 57.3) in total using My Journey 3 over the course of the trial. Eight participants (40%) used My Journey 3 for longer than 30 minutes in total.

Five participants (25%) were still using My Journey 3 six months after downloading it, however one participant never used the app after the training session (figure 3). Half of the participants (n=10) stopped using My Journey 3 within the first three months after the training session.

The average number of uses by participants for each My Journey 3 component is displayed in table 3. The most frequently accessed section was the "How are you doing?" Symptom Tracker section (median uses 3; IQR 1 to 6), however data on how frequently users accessed 'My Recovery Plan' and 'My Relapse Plan' is unavailable. The 'Information' section was accessed the fewest times, with 25% (n=5) of participants in the treatment group never using that section following the

For peer review only

training session. Just over 7% of My Journey 3 uses were initiated following a prompt from the app.

Table 3. Participant use of My Journey 3 and various sections.

	Number of times used per participant	Days used whilst having access to My Journey 3 (%)	Participants that did not use app or section – n (%)
My Journey 3	16.5 (8.5 to 23)	3.22 (1.89 to 6.36)	1 (5%)
How are you doing?	3 (1 to 6)	1.08 (0.4 to 2.12)	3 (15%)
Pill tracker	2 (1 to 3.5)	0.73 (0.36 to 1.07)	3 (15%)
Information	1 (0 to 2.5)	0.48 (0.18 to 0.7)	5 (25%)

All median (IQR), except when stated

My Journey 3 acceptability

Qualitative interviews were conducted with all participants that received My Journey 3 and the majority of clinical staff that supported its delivery. In general most service user participants found My Journey 3 to be acceptable, and a number of participants reported a clear benefit from using it. Barriers affecting use were identified including a lack of clinician support and concerns around data privacy. A key theme for staff was that they often did not have the time to provide regular support to participants with My Journey 3.

Participant outcomes

No research-related serious adverse events were recorded. Psychotic and general symptoms (measured by the PANSS) were generally low at all times for both groups suggesting a stable sample. Summary statistics and estimated effect sizes of participant outcomes are displayed in table 4. Inspection of the effect sizes and confidence intervals suggest that were no obvious differences for any outcome measure between the treatment and control group at either time-point.

For peer review only

Of the 38 participants whose patient records data were available, only five experienced a relapse during the trial, as indicated by using an acute mental health service. In the treatment group 15% of participants (3/20) experienced a relapse during the trial period compared with 11% (2/18) in the control group. We found no evidence of a difference in relapse between the two groups (odds ratio: 1.41; 95% CI: 0.21 to 9.58), but did not have sufficient power for an informative test.

Table 4. Summary statistics and unadjusted and adjusted treatment effects.

	Control (N = 13)	My Journey 3 (N = 20)	Unadjusted analysis		Analysis adjusted for baseline score	
**4-month scores**	Mean (SD)	Mean (SD)	Estimated difference	95% CI	Estimated difference	95% CI
**SIX** (Social Outcomes)	3.3 (1.9)	3.6 (1.3)	0.29	-0.84 to 1.43	0.16	-0.6 to 0.92
**MHCS** (Mental Health Confidence)	66.4 (12.7)	63 (15.8)	-3.43	-14.1 to 7.25	-4.81	-14.88 to 5.25
**QPR** (Recovery)						
Intrapersonal	47.8 (10.6)	43.2 (12.2)	-4.57	-13 to 3.87	-2.01	-8.43 to 4.49
Interpersonal	13.9 (2.4)	13.2 (2.3)	-0.72	-2.39 to 0.95	-0.42	-1.97 to 1.13
**MHCS** (Mental Health Confidence)	46.1 (9.9)	44 (11.3)	-2.08	-9.9 to 5.74	-0.19	-7.28 to 6.9
**DIALOG**						
Quality of life	4.4 (1.2)	4.5 (0.6)	0.07	-0.58 to 0.71	0.18	-0.38 to 0.74
Treatment satisfaction	5.4 (0.7)	5 (0.5)	-0.38	-0.83 to 0.06	-0.17	-0.6 to 0.25
**PANSS** (Symptom severity)						
**Positive**	9.3 (2.9)	11.4 (5.1)	2.09	-1.24 to 5.4	1.9	-0.49 to 4.3
**Negative**	10 (2.3)	11.1 (3.9)	1.05	-1.51 to 3.62	0.54	-1.6 to 2.67
**General**	23 (4)	24 (6.7)	1.21	-3.19 to 5.61	1.35	-2.68 to 5.37
**12-month scores**	Control (N = 14)	My Journey 3 (N = 16)	Unadjusted analysis		Analysis adjusted for baseline score	
	Mean (SD)	Mean (SD)	Estimated difference	95% CI	Estimated difference	95% CI
**SIX** (Social Outcomes)	3.2 (1.9)	3.5 (1.5)	0.29	-0.97 to 1.54	0.29	-0.73 to 1.3
**MHCS** (Mental Health Confidence)	66.2 (14.1)	71.1 (12.1)	4.81	-5 to 14.62	3.03	-6.04 to 12.1
**QPR** (Recovery)						
Intrapersonal	47.3 (11.5)	49.5 (11.1)	2.2	-6.25 to 10.7	3.21	-4.12 to 10.5
Interpersonal	13.6 (3.4)	15.1 (3.3)	1.44	-1.09 to 3.96	1.62	-0.89 to 4.12
**MHCS** (Mental Health Confidence)	45.6 (11.3)	49.3 (9.7)	3.61	-4.24 to 11.46	5.03	-1.67 to 11.7
**DIALOG**						
Quality of life	4.7 (0.9)	5 (0.7)	0.28	-0.31 to 0.87	0.24	-0.33 to 0.81
Treatment satisfaction	5.3 (1)	5.2 (1.2)	-0.12	-0.93 to 0.69	0.31	-0.42 to 1.04

For peer review only

**PANSS** (Symptom severity)						
Positive	9.5 (2.1)	10.2 (2.1)	0.69	-0.98 to 2.36	0.88	-0.62 to 2.38
Negative	10.2 (2.2)	10.9 (3.3)	0.77	-1.51 to 3.05	0.14	-1.56 to 1.84
General	23.5 (5.4)	22.1 (3.5)	-1.38	-4.82 to 2.07	-1	-4.57 to 2.55
**SES** (Engagement with services)	10 (6.2)	9.5 (8)	-0.4	-6.08 to 5.28	3.11	-1.57 to 7.79

Estimated differences and associated 95% Confidence Intervals from linear regression models with the control group as reference. Missing data: 4-month PANSS scores – one control group participant, one treatment group participant. 12-month PANSS scores – two control group participants. Note: 12-month SES data available for 13 control group participants, and 14 treatment group participants.

DISCUSSION

The present study examined the feasibility of conducting a RCT of a supported self-management Smartphone app in EIP services. My Journey 3 aims to facilitate recovery and prevent relapse primarily via the digital delivery of previously developed paper-and-pen self-management tools. The trial indicates that recruitment and retention in a RCT evaluating My Journey 3 is feasible, and that My Journey 3 can be delivered in EIP services. The level of My Journey 3 use was relatively low across the trial period.

Building on from extensive preliminary work with NHS staff and service users, adults with lived experience of psychosis and experts in digital health we were able to successfully develop a self-management Smartphone app that can be used in EIP services. My Journey 3 appeared to be safe with no related serious adverse events reported. My Journey 3 was successfully delivered to all participants in the treatment group, however technical problems with the intervention caused significant delays in providing access. Prior to any future evaluations technical problems with My Journey 3 will need to be identified and fixed to ensure the intervention is implemented as intended.

For peer review only

My Journey 3 use varied considerably between participants, with only a small proportion of participants frequently engaging with the app after obtaining access to it. This raises questions about whether use was at a level where it is likely that useful self-management activities were taking place: certainly not enough time was spent regularly enough for participants to be engaging in detailed monitoring of symptoms and early warning signs, tracking medication and activities and referring to crisis or recovery plans. Despite that, 40% of participants used My Journey 3 for a minimum of 30 minutes which could be an adequate amount of time for users to effectively monitor relapse signs and follow a crisis plan when needed. We have not found evidence on how regularly EIP service users make use of pen and paper self-management interventions delivered in routine settings, and this was not measured in our trial. Long-term engagement with My Journey 3 appears a challenge, but low levels of app use is a common phenomenon with market research showing that 62% of users stop using Smartphone apps after ten or fewer uses.[49]

Age has been shown to be an important factor linked to engagement with mental health apps and general Smartphone use,[50] and could partially explain differences in user engagement of My Journey 3. The treatment group however featured only a small number of participants from older age groups. We therefore lack informative data regarding app engagement for older participants and we are accordingly unable to explore if engagement and pattern of use of My Journey 3 varied between age groups.

Participant retention for research data collection was high, with 75% of the sample attending the 12-month follow-up assessment, and is comparable to other Smartphone app studies.[51] Completion rates of the SES by EIP service clinicians were much lower at the 12-month follow in comparison to baseline, potentially due to staff changes and participants being discharged from services. Recruitment strategies were largely successful, however data is lacking

For peer review only

on overall proportion of caseload recruited, reasons for non-inclusion and the numbers that were assessed for eligibility, thus limiting the conclusions we can make regarding trial feasibility.

The trial was not powered to detect effectiveness, and, as expected with our small number of participants, we found no significant differences between groups on any outcomes, with confidence intervals generally including substantial effects in either direction. Accordingly we cannot draw any conclusions regarding the potential impact of My Journey 3 as a mental health intervention. The proposed primary outcome for a full-scale trial, relapse as defined by use of an acute mental health service during the trial period, was marked by low event rates. Only five participants (12.5%) experienced a relapse during the one year follow-up period, compared with expected levels of 12% to 47%.[52] Consideration should be given to whether relapse, or our measure of relapse, is an appropriate outcome for a future RCT of this intervention. Symptom severity or alternatively patient-valued outcomes of personal recovery that self-management interventions have been shown to benefit may be more suitable primary outcomes in a future large scale trial.[12]

Strengths and limitations

My Journey 3 has been developed with extensive stakeholder input, and the intervention has been tested through lab-testing and a field study prior to the feasibility RCT. In comparison to previous studies,[51] participants had access to the app for a longer period of time. Participants' app use and usage data may be more reflective of real-world use as a result. Participant data were also collected from a wide range of methods including from participant assessments and patient records. The proposed primary outcome for a future RCT (relapse) was measured objectively and data were obtained for 95% of participants.

For peer review only

We recruited until the required number of participants was obtained rather than screening caseloads objectively: as a result we are not aware of the proportion eligible who were recruited, reasons for non-eligibility and how many EIP service users declined to take part and why. This limits our understanding of how feasible conducting a large scale trial of this intervention would be. In addition there were issues with the usage data, which impacts the reliability of our conclusions regarding how often participants engaged with My Journey 3.

The trial did not feature an active digital placebo for the control group, meaning that non-specifics of Smartphone use could not be controlled for. Furthermore data was not collected during the study period from either group regarding frequency of completing recovery work such as relapse prevention plans, recovery plans or crisis plans either in paper-and-pen or digital format, limiting our understanding of whether access to My Journey 3 facilitated increased access to self-management activities.

Although clinicians were encouraged to support participants with My Journey 3, support was not manualised and clinicians did not have personal access to the app or associated data, potentially limiting the level and quality of the support offered and therefore user engagement. Future developments of My Journey 3 should focus on effective implementation and delivery within healthcare settings, and there should be considerations on how to facilitate secure data-sharing between My Journey 3 and healthcare records or other secure web-based platforms dependent on user consent, which is likely to increase clinician engagement with the app and its utility.[53]

We did also not define pre-specified criteria for assessing the feasibility of a RCT and the acceptability of My Journey 3. Instead we will consider all findings from the trial, app usage data and feedback from qualitative interviews yet to be reported in determining whether My Journey 3

For peer review only

will be evaluated in a full-scale trial. This allows all data from the RCT to be thoroughly considered, but may be a less objective approach in determining feasibility than using pre-defined criteria. Although the trial was not designed to assess intervention effectiveness, participants and trial researchers were not blinded to group allocation, and as such could have led to an inflation of any observed effects.

Finally the sample consisted of Android Smartphone users who were generally stable and in an appropriate stage of recovery to consider using a self-management Smartphone app. Participants may therefore not be representative of all EIP service users. Furthermore contact with a researcher within a trial context could have led to increased intervention engagement that would not occur in a real-world clinical environment.

Conclusions

We developed and delivered a self-management Smartphone app for first-episode psychosis in a trial context. Participants were successfully recruited, most engaged at least to some extent with the intervention, and they had high follow-up rates over the one year trial period. Based on the data presented the trial methods appear feasible. My Journey 3 was shown to be safe, but the level of use was lower than anticipated thus potentially limiting its utility, although usage levels were higher than reported for downloaded apps in the general population.

If My Journey 3 is to be further tested in a research setting, attention needs to be given to engagement, a challenge associated with many digital tools in mental health.[54] Further usability testing in lab and field settings may also be a means to improving engagement. Other potential strategies including making more efforts to engage clinicians as well as service users with My Journey 3 by giving them access to the tool and to aspects of the planning and monitoring that

For peer review only

service users conduct through it. The app could also potentially be offered as part of a blended approach to self-management, with pen and paper tools also used and as a whole service strategy for implementation of self-management. Refinements required before participating to a full trial including participant and assessor blinding and manualised clinician support should be considered prior to conducting a future RCT.

For peer review only

Acknowledgements: The ARIES research team are grateful to their software collaborators MyOxygen for their technical development and hosting of My Journey 3 and to Ali Mousa for his valuable contribution to the development of the original My Journey app. We are grateful to Max Birchwood for his permission to incorporate 'Back in the Saddle' in to My Journey 3. We are grateful to Rachel Perkins for her permission to adapt the Personal Recovery Plan resource and incorporate in to My Journey 3.

Author Contributions: SJ is the Chief Investigator, based at University College London, DO the co-Chief Investigator, and TS the project manager. The trial design was developed by SJ, DO, BLE and PO. SA, HR, PO and ME have led on the development of the intervention. TS conducted the statistical analysis, with advice from RJ. TS wrote the draft of the paper, which was revised and approved by all authors. All authors approved the final manuscript.

Funding: The research is funded by the National Institute for Health Research (NIHR) Collaboration for Leadership in Applied Health Research and Care North Thames at Barts Health NHS Trust (NIHR CLAHRC North Thames). The views expressed in this article are those of the authors and not necessarily those of the NHS, the NIHR or the Department of Health and Social Care. SJ, DO and BLE are supported by the NIHR Mental Health Research Policy Unit, the NIHR Collaboration for Leadership in Applied Health Research and Care (CLAHRC) North Thames and the UCLH Biomedical Research Centre.

Data statement: The datasets generated during and/or analysed during the current study will be made available two years after the trial end.

Competing interests: None declared.

For peer review only

Legends:

Figure 1. Screenshots of the My Journey 3 app. a) The homescreen, b) the ‘My goals’ section of the recovery plan, c) the ‘Coping with early warning signs’ section of the relapse prevention plan, d) an example item from the Symptom Tracker, e) the Information section.

Figure 2. CONSORT diagram of the ARIES feasibility trial. Note: DNA, did not attend.

Figure 3. Bar chart displaying how long after the training session participants disengaged with My Journey 3. For the participants aged over 35, one participant disengaged in the first month (second column), one between 3 and 6 months (fourth column) and the other 35+ participant was still using My Journey 3 six months after the training session (fifth column).

For peer review only

REFERENCES

1 Bird V, Premkumar P, Kendall T, et al. Early intervention services, cognitive–behavioural therapy and family intervention in early psychosis: systematic review. *Brit J Psychiat*. 2010;197:350-6.

2 Tsiachristas A, Thomas T, Leal J, et al. Economic impact of early intervention in psychosis services: results from a longitudinal retrospective controlled study in England. *BMJ Open* 2016;6:e012611.

3 Alvarez-Jiménez M, Parker AG, Hetrick SE, et al. Preventing the second episode: a systematic review and meta-analysis of psychosocial and pharmacological trials in first-episode psychosis. *Schizophrenia Bull*. 2009;37:619-30.

4 Kam SM, Singh SP, Upthegrove R. What needs to follow early intervention? Predictors of relapse and functional recovery following first-episode psychosis. *Early Interv Psychia*. 2015;9:279-83.

5 Puntis S, Oke J, Lennox B. Discharge pathways and relapse following treatment from early intervention in psychosis services. *BJPsych open* 2018;4:368-74.

6 Leucht S, Heres S. Epidemiology, clinical consequences, and psychosocial treatment of nonadherence in schizophrenia*. J Clin Psychiatry* 2006;67:3-8.

7 Haddock G, Eisner E, Boone C, et al. An investigation of the implementation of NICE-recommended CBT interventions for people with schizophrenia. *J Ment Health* 2014:1;23:162-5.

8 Mueser KT, Meyer PS, Penn DL, et al. The Illness Management and Recovery program: rationale, development, and preliminary findings. *Schizophrenia Bull* 2006;32:S32-43.

For peer review only

9 Mueser KT, Corrigan PW, Hilton DW et al. Illness management and recovery: A review of the research. *Psychiat Serv* 2002;53:1272-84.

10 Cook JA, Copeland ME, Jonikas JA, et al. Results of a randomized controlled trial of mental illness self-management using Wellness Recovery Action Planning. *Schizophrenia Bull* 2011;38:881-91.

11 Färdig R, Lewander T, Melin L, et al. A randomized controlled trial of the illness management and recovery program for persons with schizophrenia. *Psychiat Serv* 2011;62:606-12.

12 Lean M, Fornells-Ambrojo M, Milton A, et al. Self-management interventions for people with severe mental illness: systematic review and meta-analysis. *Brit J Psychiat* 2019;22:1-9.

13 NICE Guidance - Psychosis and schizophrenia in adults: prevention and management. https://www.nice.org.uk/guidance/cg178/ifp/chapter/peer-support-and-self-management [accessed 2019-01-30]

14 Whitehead L, Seaton P. The effectiveness of self-management mobile phone and tablet apps in long-term condition management: a systematic review. *J Med Internet Res* 2016;18:e97.

15 Poushter J. 2016 Smartphone ownership and internet usage continues to climb in emerging economies. URL: http://www.pewglobal.org/2016/02/22/smartphone-ownership-and-internet-usage-continues-to-climb-in-emerging-economies/ [accessed 2019-01-30]

16 Wigginton C, Curran M, Broedeur C. 2017. Global mobile consumer trends, 2nd edition. URL: https://www2.deloitte.com/content/dam/Deloitte/us/Documents/technology-media-telecommunications/us-global-mobile-consumer-survey-second-edition.pdf. [accessed 2019-07-04]

For peer review only

17 Firth J, Cotter J, Torous J, et al. Mobile phone ownership and endorsement of "mHealth" among people with psychosis: a meta-analysis of cross-sectional studies. *Schizophrenia Bull* 2015;42:448-55.

18 Robotham D, Satkunanathan S, Doughty L, Wykes T. Do We Still Have a Digital Divide in Mental Health? A Five-Year Survey Follow-up. *J Med Internet Res* 2016;18:e309.

19 Naslund JA, Aschbrenner KA, Bartels SJ. How people with serious mental illness use smartphones, mobile apps, and social media. *Psychiatr Rehabil J* 2016;39:364-367.

20 Aref-Adib G, O'Hanlon P, Fullarton K, et al. A qualitative study of online mental health information seeking behaviour by those with psychosis. *BMC Psychiatry* 2016;16:232.

21 Berry N, Lobban F, Bucci S. A qualitative exploration of service user views about using digital health interventions for self-management in severe mental health problems. *BMC Psychiatry* 2019;19:35.

22 Proudfoot J. The future is in our hands: the role of mobile phones in the prevention and management of mental disorders. *Aust NZ J Psychiat* 2013;47:111-3.

23 Gottlieb JD, Romeo KH, Penn DL, et al. Web-based cognitive–behavioral therapy for auditory hallucinations in persons with psychosis: a pilot study. *Schizophr Res* 2013;145:82–87.

24 Granholm E, Ben-Zeev D, Link PC, et al. Mobile Assessment and Treatment for Schizophrenia (MATS): a pilot trial of an interactive text-messaging intervention for medication adherence, socialization, and auditory hallucinations. *Schizophr Bull* 2011;38:414-25.

25 Ben-Zeev D, Kaiser SM, Brenner CJ, et al. Development and usability testing of FOCUS: A smartphone system for self-management of schizophrenia. *Psychiatr Rehabil J* 2013;36:289-296.

For peer review only

26 Alvarez-Jimenez M, Bendall S, Lederman R, et al. On the HORYZON: moderated online social therapy for long-term recovery in first episode psychosis. *Schizophr Res* 2013;143:143-9.

27 Bonet L, Izquierdo C, Escartí MJ, et al. Use of mobile technologies in patients with psychosis: A systematic review. *Rev Psiquiatr Salud Ment* 2017;10:168-78.

28 Rus-Calafell M, Schneider S. Are we there yet?!—a literature review of recent digital technology advances for the treatment of early psychosis. *mHealth* 2019. doi: 10.21037/mhealth.2019.09.14

29 Camacho E, Levin L, Torous J. Smartphone Apps to Support Coordinated Specialty Care for Prodromal and Early Course Schizophrenia Disorders: Systematic Review. *J Med Internet Res* 2019;21:e16393.

30 Bucci S, Barrowclough C, Ainsworth J, et al. Actissist: Proof-of-Concept Trial of a Theory-Driven Digital Intervention for Psychosis. *Schizophrenia Bull* 2018; sby032.

31 JMIR. URL: https://support.jmir.org/hc/en-us/articles/115001389307-Does-my-trial-RCT-have-to-be-registered- [accessed 2019-10-08]

32 Steare T, O'Hanlon P, Eskinazi M, et al. App to support Recovery in Early Intervention Services (ARIES) study: protocol of a feasibility randomised controlled trial of a self-management Smartphone application for psychosis. *BMJ Open* 2019;9:e025823.

33 Eldridge SM, Chan CL, Campbell MJ, et al. CONSORT 2010 statement: extension to randomised pilot and feasibility trials. *BMJ* 2016;355:i5239.

34 National Institute for Health and Care Excellence. Implementing the Early Intervention in Psychosis Access and Waiting Time Standard: Guidance. London: NHS England Publications. 2016. p. 1-57.

For peer review only

35 Julious SA. Sample size of 12 per group rule of thumb for a pilot study. *Pharm Stat* 2005;287-91.

36 Plaistow J, Birchwood M. Back in the saddle: a guide to relapse prevention. In: Birchwood M, Fowler D, Jackson C, editors. Early Intervention in Psychosis: A Guide to Concepts, Evidence and Interventions. Chichester: McGraw-Hill; 1996. p. 239-244.

37 Perkins R, Rinaldi M. Taking back control: a guide to planning your own recovery. South West London: St George's Mental Health NHS Trust, 2007.

38 Priebe S, Watzke S, Hansson L, et al. Objective social outcomes index (SIX): a method to summarise objective indicators of social outcomes in mental health care. *Acta Psychiat Scand* 2008;118:57-63.

39 Carpinello SE, Knight EL, Markowitz FE, et al. The development of the Mental Health Confidence Scale: A measure of self-efficacy in individuals diagnosed with mental disorders. *Psychiatr Rehabil J* 2000;23:236-243.

40 Neil ST, Kilbride M, Pitt L, et al. The questionnaire about the process of recovery (QPR): a measurement tool developed in collaboration with service users. *Psychosis* 2009;1:145-55.

41 Tennant R, Hiller L, Fishwick R, et al. The Warwick-Edinburgh mental well-being scale (WEMWBS): development and UK validation. *Health Qual Life Out* 2007;5:63.

42 Priebe S, McCabe RO, Bullenkamp J, et al. Structured patient–clinician communication and 1-year outcome in community mental healthcare. *Brit J Psychiat* 2007;191:420-6.

43 Kay SR, Fiszbein A, Opfer LA. The positive and negative syndrome scale (PANSS) for schizophrenia. *Schizophrenia Bull* 1987;13:261-76.

For peer review only

44 Tait L, Birchwood M, Trower P. A new scale (SES) to measure engagement with community mental health services. *J Ment Health* 2002;11:191-8.

45 Johnson S, Lamb D, Marston L, et al. Peer-supported self-management for people discharged from a mental health crisis team: a randomised controlled trial. *Lancet* 2018;392:409-18.

46 Sekhon M, Cartwright M, Francis JJ. Acceptability of healthcare interventions: An overview of reviews and development of a theoretical framework. *BMC Health Serv Res* 2017;17(1):1–13.

47 Kirkbride JB, Fearon P, Morgan C, et al. Heterogeneity in incidence rates of schizophrenia and other psychotic syndromes: findings from the 3-center AeSOP study. Arch Gen Psychiat. 2006:63;250-8.

48 Adamson V, Barrass E, McConville S, et al. Implementing the access and waiting time standard for early intervention in psychosis in the United Kingdom: An evaluation of referrals and post-assessment outcomes over the first year of operation. *Early Interv Psychia* 2018:12;979-86.

49 Localytics. 21% of users abandon an app after one use. http://info.localytics.com/blog/21-percent-of-users-abandon-apps-after-one-use. [accessed 2019-05-16]

50 Beard C, Silverman AL, Forgeard M, Wilmer MT, Torous J, Björgvinsson T. Smartphone, social media, and mental health app use in an acute transdiagnostic psychiatric sample. *JMIR mHealth uHealth* 2019;7(:e13364.

51 Firth J, Torous J. Smartphone apps for schizophrenia: a systematic review. *JMIR Mhealth Uhealth* 2015;3.

For peer review only

52 Alvarez-Jimenez M, Priede A, Hetrick SE, et al. Risk factors for relapse following treatment for first episode psychosis: a systematic review and meta-analysis of longitudinal studies. *Schizophr Res* 2012;139:116-28.

53 Torous J, Woodyatt J, Keshavan M, Tully LM. A new hope for early psychosis care: the evolving landscape of digital care tools. *British J Psychiat* 2019;214:269-72.

54 Aref-Adib G, McCloud T, Ross J, et al. Factors affecting implementation of digital health interventions for people with psychosis or bipolar disorder, and their family and friends: a systematic review. *Lancet Psychiat* 2018;6:257-66.

For peer review only

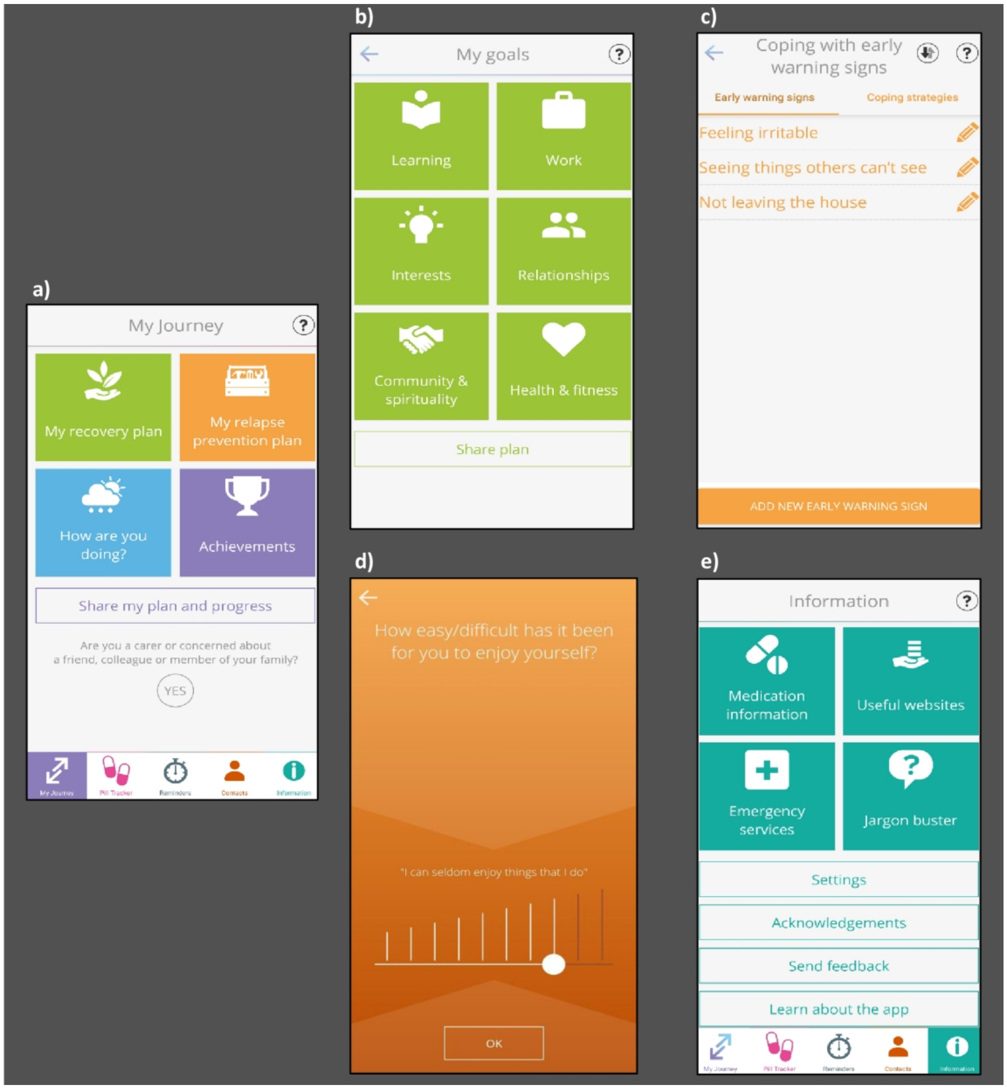

Figure 1. Screenshots of the My Journey 3 app. a) The homescreen, b) the 'My goals' section of the recovery plan, c) the 'Coping with early warning signs' section of the relapse prevention plan, d) an example item from the Symptom Tracker, e) the Information section.

169x182mm (300 x 300 DPI)

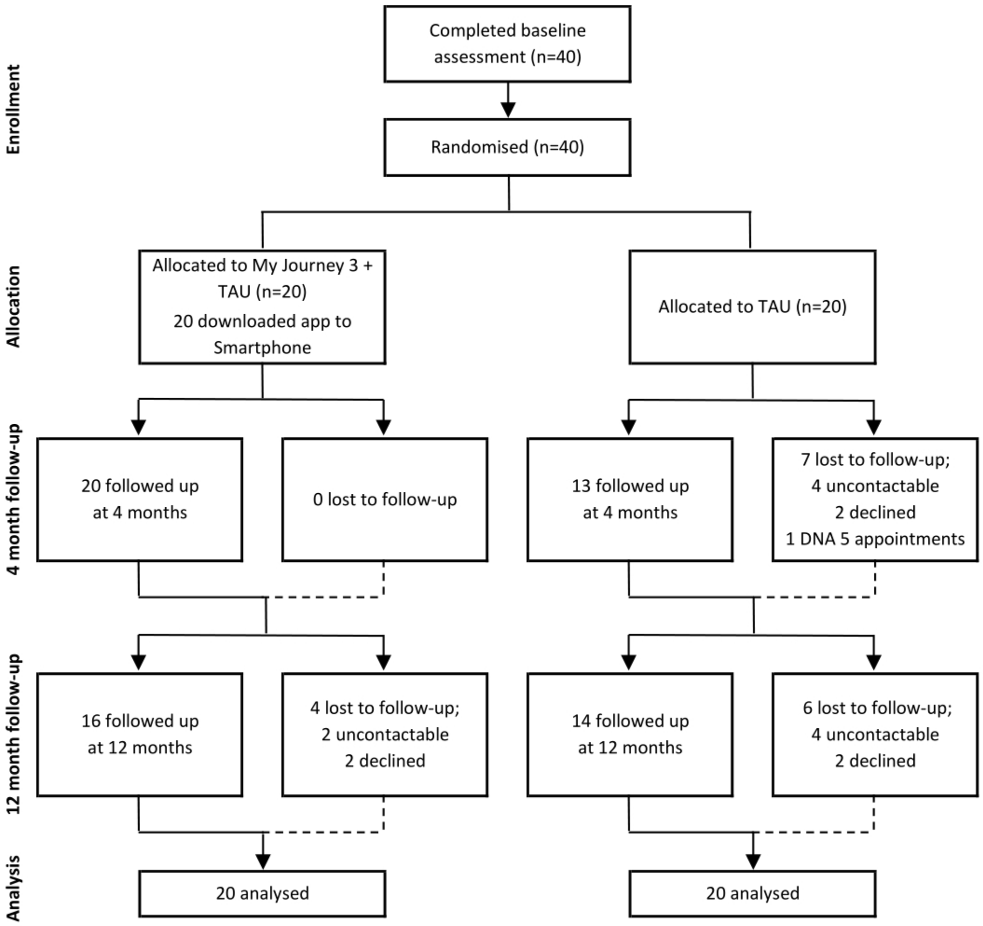

Figure 2. CONSORT diagram of the ARIES feasibility trial. Note: DNA, did not attend.

90x85mm (300 x 300 DPI)

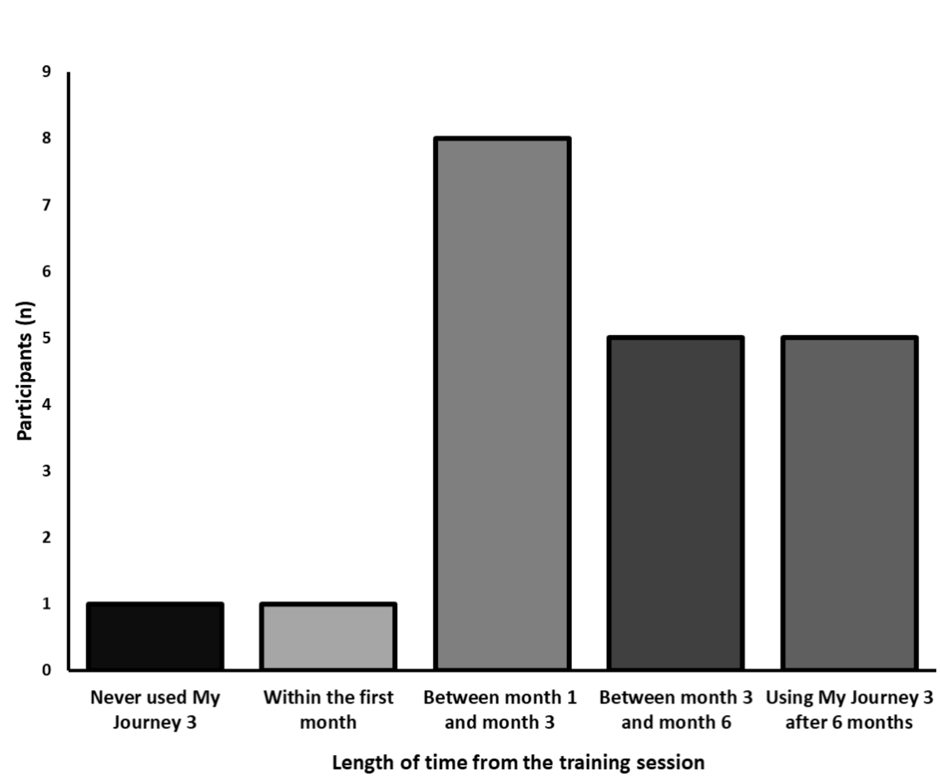

Figure 3. Bar chart displaying how long after the training session participants disengaged with My Journey 3.

101x79mm (300 x 300 DPI)

Service user participant consent form

Study Title: App to support Recovery In Early intervention Services (the ARIES study): Pilot randomised controlled trial of a self-management smartphone application

Principal Investigators: Professor Sonia Johnson and Professor David Osborn

Please Initial Each Box

1. I confirm that I have read and understood the Participant Information Sheet V5 dated 29/05/2017 for the above study and have had the opportunity to ask questions about the study. ☐

2. I understand that my participation is voluntary and that I am free to withhold personal information or to withdraw my participation at any time, without giving any reason, and without my medical care or legal rights being affected. ☐

3. I understand that if I choose to withdraw from the study that any data that I have already provided for the purposes of the research will be kept and used by the research team. ☐

4. I give permission for my General Practitioner (GP) and my Early Intervention team to be told I am participating in this study. ☐

5. I understand that relevant sections of my medical notes and data collected during the study may be looked at by regulatory authorities or from the NHS Trust, where it is relevant to my taking part in this research. I give permission for these individuals to have access to my records. ☐

6. I understand that I will be given a £20 gift as cash for my participation in each study assessment. ☐

7. I agree to the research team consulting NHS electronic records to investigate my diagnosis, medication, and mental health service use, and give them permission to do so even if I choose to no longer participate in the intervention, or they are not able to carry out further study interviews with me. ☐

8. I understand that in the event that I disclose information which may indicate new risk to myself or others, the researcher will be obliged to follow NHS Trust risk procedures that may require release of my personal data. ☐

9. I give permission for findings from the study to be written up for publication. Any publication will not identify me. ☐

10. I give permission to be audio recorded where required for the purposes of the study. I understand these audio-recordings will be transcribed and anonymised and audio recordings destroyed after the study. I give permission for direct quotations taken from this interview to be included in papers written for publication. Any quotation would not identify me. ☐

11. I give permission for the research team to collect data from the My Journey 3 app regarding the frequency, duration, and pattern of my use of it. I understand that no personal information will be collected from the app. ☐

12. I give permission for non-identifiable data to be shared with other research teams for research purposes. ☐

For peer review only - http://bmjopen.bmj.com/site/about/guidelines.xhtml

13. I agree to take part in this study. ☐

Name of participant	Date	Signature
Name of Researcher taking consent	Date	Signature

For peer review only

For peer review only - http://bmjopen.bmj.com/site/about/guidelines.xhtml

App to support Recovery In Early intervention Services (the ARIES study): Usability testing and pilot randomised controlled trial of a supported self-management smartphone application for psychosis

RESEARCH PROTOCOL

**Study Title:**	App to support Recovery In Early intervention Services (the ARIES study): Usability testing and pilot randomised controlled trial of a self-management smartphone application
**Study Acronym:**	ARIES: App to support Recovery In Early intervention Services

**Principal Investigators**:	Professor Sonia Johnson [a,b]	s.johnson@ucl.ac.uk
	Professor David Osborn [a,b]	d.osborn@ucl.ac.uk
**Research Team**:	Dr Bryn Lloyd-Evans [a]	b.lloyd-evans@ucl.ac.uk
	Dr Golnar Aref-Adib [a,b]	g.aref-adib@ucl.ac.uk
	Dr Helen Rostill [c]	Helen.Rostill@sabp.nhs.uk
	Sharon Dean [c]	Kate.Sigov@sabp.nhs.uk
	Christine Gee [c]	Christine.Gee@sabp.nhs.uk
	Puffin O'Hanlon [a,b]	p.hanlon@ucl.ac.uk
	Dr Michelle Eskinazi [a,b]	Michelle.eskinazi.15@ucl.ac.uk

[a] *Division of Psychiatry, University College London*

[b] *Camden and Islington NHS Foundation Trust*

[c] *Surrey and Borders NHS Foundation Trust*

Camden and Islington NHS Foundation Trust

Surrey and Borders Partnership NHS Foundation Trust

NHS National Institute for Health Research

Collaboration for Leadership in Applied Health Research and Care North Thames

For peer review only

For peer review only - http://bmjopen.bmj.com/site/about/guidelines.xhtml

Contents

1. Background ... 3
2. Phase I: Usability testing ... 6
  2.2 Setting ... 6
  2.3 Participants ... 6
  2.4 The intervention: My Journey 3 App ... 7
  2.5 Lab-based usability testing procedures ... 10
    2.5.1 Recruitment and consent ... 10
2.6 Field study procedure ... 12
  2.6.1 Field study Measures ... 12
  2.6.2 Recruitment and consent ... 13
  2.6.3 Baseline assessment ... 14
  2.6.4 Intervention Procedure ... 14
  2.6.5 Four week follow-up assessment ... 15
  2.6.6 Clinician interview ... 15
  2.7 Analysis ... 15
3. Phase II: Pilot RCT ... 16
  3.1 Setting ... 16
  3.2 Sample size ... 16
  3.3 Participants ... 16
  3.4 Measures ... 17
  3.5 Procedures ... 18
    3.5.1 Recruitment and consent ... 18
    3.5.2 Randomisation and blinding ... 19
    3.5.3 Assessment ... 20
    3.5.4 Control procedure ... 21
    3.5.5 Intervention procedure ... 21
    3.5.6 Clinician interview (intervention arm only) ... 21
    3.5.8 Data from patient records ... 22
  3.6 Analysis ... 22
4. Data storage ... 23
5. App safety, security, and maintenance ... 23
6. Research governance and oversight ... 24
7. Dissemination ... 25
8. Timeline ... 25

For peer review only - http://bmjopen.bmj.com/site/about/guidelines.xhtml

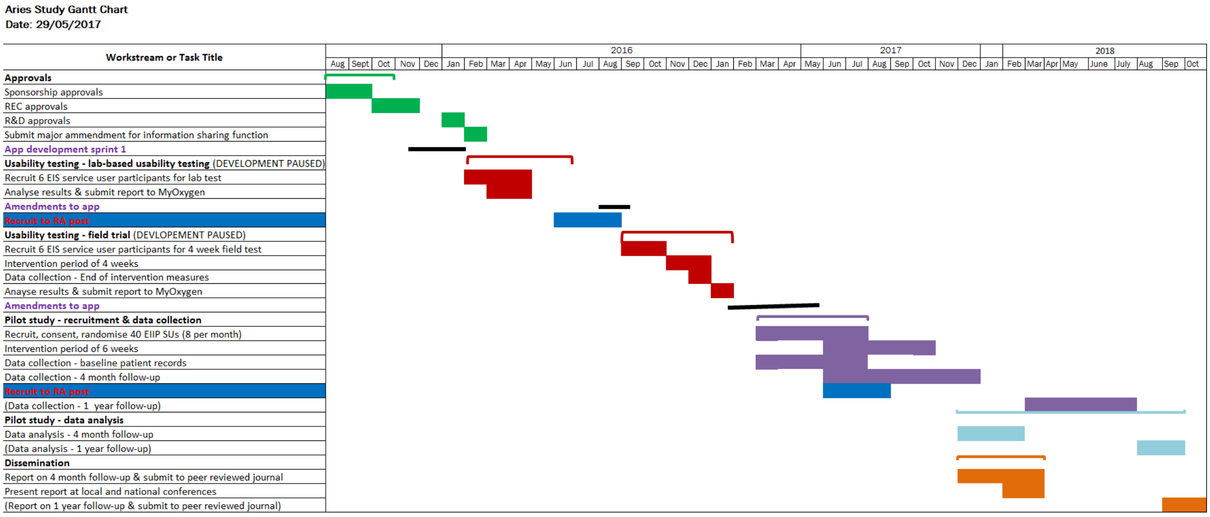

.... **Error! Bookmark not defined.**

9. References 25

Keywords

Psychosis, recovery, self-management, recovery, relapse prevention, smartphone application, early intervention

1. Background

Despite improved outcomes associated with Early Intervention Services (EIS) ((Bird, Premkumar et al. 2010)), substantial challenges remain. Firstly, many young people do not attain functional recovery following early intervention for a first episode of psychosis (Alverez-Jimenez et al., 2012). Secondly, relapse rates remain high (Alvarez-Jimenez, 2011): Between 35% and 75% of first episode psychosis patients will experience a relapse (Addington & Addington, 2007). Evidence suggests that the benefits of EIS may not persist after discharge (Gafoor et al., 2010), and the five year 'critical period' of risk for relapse after psychosis onset (Norman et al., 2011) extends well beyond the availability of EIS (typically two to three years). Finally, the long-term engagement of young people in relapse-prevention interventions is a significant issue (Alvarez-Jimenez et al., 2009). There is a need to respond innovatively to these challenges in order to extend the effectiveness of Early Intervention Services.

Psychosocial interventions, including self-management, have demonstrated benefits above and beyond those of antipsychotic medication, including reduced risk of relapse and improved functional recovery (Mueser et al., 2013). Self-management is designed to empower people as active agents in their own recovery by enabling them to develop skills such as monitoring symptoms and medication, identifying and avoiding stressors, and using coping strategies (Mueser et al., 2006). However, these interventions reach only a small

For peer review only - http://bmjopen.bmj.com/site/about/guidelines.xhtml

proportion of those who might benefit. Barriers to access include high delivery costs and the stigma attached to seeking treatment (Alvarez-Jimenez et al., 2012a).

The rapid development and uptake of digital technologies has transformed the way in which people communicate, learn and, increasingly, seek health information (OfCom 2013). A growing body of evidence suggests that people with psychosis are adopting digital technology in a similar way to the general population (Schrank et al., 2010; Highton-Williamson et al., 2014). In this context, there is an opportunity to leverage digital technologies to deliver cost-effective, time-unlimited, non-stigmatising support for people using EIS services.

Findings from four systematic reviews of emerging research suggest that ICT-based interventions are acceptable and feasible for people with psychosis and show promise in improving various outcomes including psychotic and mood symptoms, medication adherence, hospital admissions and social connectedness (Valimaki et al., 2012; Alvarez-Jimenez et al., 2014; Kasckow et al., 2014; Van der Krieke et al., 2014).

However, although young people are the biggest consumers of information and communication technologies (ICTs; OfCom, 2014) and appear to have positive attitudes to e-health services (Lederman et al., 2011, Birnbaum et al., 2015), very few studies have investigated digital technology enabled interventions for young people with first episode psychosis. No existing studies have evaluated a self-management smartphone application for young people recovering from first episode psychosis.

Smartphones are accessible, portable, increasingly affordable, and used by 88-90% of 16-34 year olds in the UK. As such, they may be particularly suited to the provision of time-unlimited support for young people with psychosis in real-world contexts, when it is most needed. Mobile applications, or apps – computer programs that enhance the functionality of mobile devices – offer highly sophisticated platforms for the provision of interactive, personalised self-management interventions. Emerging evidence suggests that psychosocial interventions delivered via apps are acceptable and potentially beneficial for people experiencing mental health problems, including young people (Seko et al., 2014) and adults with persistent symptoms of psychosis (Ben-Zeev et al., 2014).

Based on the preliminary evidence above, we have developed a product specification for further development of the *My Journey* smartphone application. The first version of My Journey was designed with and for young people with first episode psychosis by Surrey and Borders Partnership NHS FT and was made freely available for download in April 2013. Several recommendations have been put forward for the development of acceptable, safe and effective e-health interventions for psychosis (Rotundi et al., 2007, Valimaki et al, 2008, Depp et al., 2010; Sharkey et al., 2011). These include careful consideration of users' needs, the active involvement of stakeholders throughout development, and the use of usability and

For peer review only - http://bmjopen.bmj.com/site/about/guidelines.xhtml

pilot testing. In consultation with e-Health experts, EIS clinicians and people with lived experience of psychosis, we have adapted existing paper-and-pen self-management intervention components used widely in NHS services to be suitable for delivery in an app format. This resulted in several iterations of a product specification for the new version of My Journey. The app will comprise of four main intervention components: information and advice about psychosis, mental health, and mental health services; self-monitoring of symptoms and medication adherence; identifying things to do to keep well and setting and tracking personal recovery goals; and relapse prevention and crisis planning.

1.1. Aims

The proposed study has two phases. In phase I we will conduct lab-based usability testing and a four week field study of the My Journey 3 app with participants accessing Early Intervention Services. The three main objectives of this phase are:

1. To identify whether the My Journey app is usable and acceptable to Early Intervention Service users;

2. To identify any necessary alterations or enhancements to the design and content of the app prior to final programming of the My Journey 3 app for deployment in the subsequent pilot RCT;

3. To explore the usage, acceptability, and perceived usefulness of the My Journey 3 app in Early Intervention service users' daily lives;

4. To explore the feasibility and acceptability of the assessment and intervention procedures prior to the pilot trial.

Phase II will be a prospective pilot randomised controlled trial (RCT) to test a supported self-management app plus treatment as usual (TAU) compared to TAU alone (see figure 1). The three main objectives are:

1. Piloting the intervention: To identify whether the My Journey app is feasible and acceptable to Early Intervention service users and to identify any necessary modifications to the intervention content, design or delivery prior to a definitive RCT.

2. Piloting the trial procedures: To test the feasibility and acceptability of trial parameters (recruitment and retention rates, eligibility criteria, assessment, randomisation and allocation procedures) for a definitive RCT.

For peer review only - http://bmjopen.bmj.com/site/about/guidelines.xhtml

3. Piloting the methods of analysis: To test procedures for evaluating intervention engagement and outcomes, to inform the design of a definitive RCT. It is not appropriate to engage in hypothesis testing in a pilot study (Arain et al., 2010; Leon et al, 2011) as the study is likely, by definition, to be underpowered. Rather, the pilot trial will inform the selection of primary and secondary outcome measures and sample size calculation for a future fully-powered trial.

2. Phase I: Usability testing

2.2 Setting

Participants will be recruited from four Early Intervention Service teams across Camden and Islington NHS Foundation Trust and East London NHS Foundation Trust.

2.3 Participants

Service User Participants

Participants will be people with psychosis, aged 16 or older who are currently engaged with an Early Intervention Service within Camden and Islington NHS Foundation Trust or East London NHS Foundation Trust. Six participants will be recruited to take part in each of the two usability testing phases.

Early Intervention Services accept people onto their caseload who 1) have developed symptoms of a psychotic illness for the first time, and 2) are experiencing positive psychotic symptoms that have persisted for at least a week that are accompanied by significant risk and/or decline in functioning.

Participants will only be eligible for the ARIES study (the lab-based usability testing, field trial and pilot randomised controlled trial) if they own an Android smartphone. My Journey 3 will only be developed for Android at this stage of development, in order to evaluate its usability, acceptability and usefulness before seeking further funding to develop the app for other platforms (e.g. windows, iOS). Regrettably, we do not have sufficient resources in the study to cover the cost of providing participants with a smartphone for the trial. However, the advantage of using participants' own phones is that they are likely to be more familiar with and adept at using the device. In the general population in the UK, 88% of 16-24 year olds and 84% of 25-34 year olds use a smartphone, with Android holding the largest share of the market (OfCom, 2014). Whether this is the case for people accessing EIS services is unclear. Demographic questions in the interview schedules are designed to allow us to examine the

For peer review only - http://bmjopen.bmj.com/site/about/guidelines.xhtml

For peer review only

representativeness of the sample, and this will be taken into account when considering the generalisability of the results.

Inclusion criteria

Participants will be eligible for the study if they:

1) Are aged 16 or older
2) Have a diagnosis of psychosis
3) Own an Android smartphone
4) Are on the caseload of an EIS service
5) Speak and understand English

Exclusion criteria

Participants will be excluded from the study on the basis that they:

1) Are aged 15 or younger
2) In the view of their EIS team, pose such a high risk to others that it would be unsafe for a researcher to meet with them, even in a mental health service setting
3) Lack capacity to provide informed consent to take part in the study

EIS clinician participants

Up to six EIS clinicians who have supported service user participants with the app intervention will be recruited to take part in a qualitative interview in the field study only. These clinicians members will be employed by Camden and Islington NHS Foundation Trust or East London NHS Foundation Trust in an EIS service.

Inclusion Criteria

Participants will be eligible to take part in a qualitative interview in the field study if they:

1) Are aged 18 or older
2) Are employed by a participating trust in an EIS service and are currently in work
3) Have supported an EIS service user participant with the app intervention in the field study

2.4 The intervention: My Journey 3 App

The My Journey smartphone application was developed by Surrey and Borders Partnership NHS Foundation Trust (SABP) with young people who have experienced psychosis and accessed the SABP Early Intervention in Psychosis service (EIIP). The first version of the app was launched in April 2013 and is freely available for download from the Google Play store. The app features a self-monitoring tool and tracker, advice on how to improve symptoms and functioning, a medication tracker, and information about mental health and mental health services.

For peer review only - http://bmjopen.bmj.com/site/about/guidelines.xhtml

My Journey 3 will be a further development of this app to incorporate personalised recovery and relapse prevention planning tools. The conceptual framework for My Journey 3 is grounded in the stress-vulnerability model of psychosis (Liberman et al., 1986), which proposes that the interaction between psychological and social stressors and biological and psychological vulnerabilities determines the course of psychosis. Self-management strategies based on the stress-vulnerability model aim to target this interaction through facilitating awareness of the impact of stressors and learning of coping strategies to ameliorate their impact. In designing the content of the My Journey 3 app, we have drawn on self-management material from two paper and pen self-management tools and adapted it to be suitable for delivery as a smartphone app.

In developing the product specification, we have taken recommendations from studies that have investigated the potential challenges of digital technology user interfaces for people who have experienced psychosis. These include taking steps to minimise the amount of text and information on each screen, unnecessary stimuli, jargon, and the need to remember previous steps in the navigation process (Rotondi et al., 2007; Valimaki et al., 2008; Schrank et al., 2010)

The development of My Journey 3 consists of the following stages:

Stage 1: Content translation from existing paper and pen tools to smartphone app

Two recovery and relapse prevention booklets were drawn on in developing the product specification for the My Journey 3 app. "My Personal Recovery Plan" was adapted for the UCL CORE study from recovery planning resources compiled by Dr Rachel Perkins and colleagues at South West London and St Georges NHS Foundation Trust, in turn informed by resources including the Wellness Recovery Action Plan (Cook et al., 2009). "Back in the Saddle" (Plaistow & Birchwood, 1996) was developed to support relapse prevention work in North Birmingham Mental Health Trust Early Intervention Service, and is widely used in Early Intervention Services nationally. The wireframe and product specification for My Journey 3 was developed after several iterative cycles of design and consultation with experts in the fields of e-health and human computer interaction, early intervention clinicians, and peer support workers, who have experience in delivering a supported self-management intervention. In addition to the components of the existing version of the My Journey App, My Journey 3 will include two structured sections focusing on recovery planning and relapse Prevention, which will enable users to interactively:

- Identify strategies and coping resources to maintain wellbeing
- Set and track progress towards personal recovery goals

For peer review only - http://bmjopen.bmj.com/site/about/guidelines.xhtml

- Identify personal early warning signs of relapse and strategies and coping mechanisms to put in place should they experience these

- Create a "relapse drill": an action plan to follow in times of crisis in order to avoid or mitigate relapse

The app will make use of push notifications to encourage engagement with the app. These notifications have been designed to be discreet so as not to compromise user privacy or become annoying (see wireframe). The user will receive 1) weekly notifications inviting them to engage with the app that will appear on the user's smartphone home screen: "My Journey: we haven't seen you for a while – would you like to log on?" Users will be able to set the time of day that they receive these notifications; 2) notifications to track whether they have taken their medication that will appear on the user's home screen as "Reminder from My Journey" (see wireframe) – their frequency will be determined by the frequency with which they have indicated they take prescribed medication; 3) Activity reminders (e.g. for steps towards personal goals or things to do to keep well) that will also appear on the user's home screen as "Reminder from my Journey" – again, their timing and frequency will be determined by user input.

There will be a sharing functionality within the MyJourney App should users wish to share some or all of their data with a trusted mental health worker, family member, friend or other trusted third party. This will use the built-in sharing functionality of the user's smart device (such as e-mail) and the participant information sheet makes it clear that it is the users' responsibility to ensure this meets their own data security requirements. Both the app and the study participant information advises participants that all content and information available or shared through the My Journey app is for reference and information purposes only, and is not designed as a substitute for seeking professional medical advice, diagnosis, or treatment.

Stage 2: Technical development of My Journey 3

The app will be programmed by an app development company with experience in the development of health and wellbeing apps for mental health populations according the specification developed in Stage 1. Programming will proceed iteratively, in consultation with the research team. Firstly, high-fidelity paper wireframes will be developed to illustrate the app design and user interface. Feedback on these will be invited from EIS clinicians and service users and will inform the programming of the first prototype of the My Journey 3 native smartphone app. This prototype will be further developed, informed by the results of the Lab-based usability testing and field trial, before final programming prior to the pilot trial.

For peer review only - http://bmjopen.bmj.com/site/about/guidelines.xhtml

My Journey 3 will be a native smartphone app developed for an Android platform. An Android platform was selected for this stage of development and testing as the majority of UK smartphone owners use the Android operating system (http://uk.kantar.com/tech/mobile/). If results of the ARIES study suggest that the My Journey 3 app is acceptable, feasible, and potentially beneficial, the research team will seek further funding to develop the app for other platforms, including iOS.

2.5 Lab-based usability testing procedures

2.5.1 Recruitment and consent

1) Researchers will seek help from clinicians in participating EISs to identify EIS service users who meet the study's inclusion criteria. At this stage, clinicians will screen out service users who meet the study exclusion criteria or who are not interested in taking part in the study.
2) Clinicians who are known to the potential participant will make initial contact with them to give a brief explanation of the study and to ask if they are willing for the clinician to pass their name and preferred contact details to the research team so that they can be contacted by a researcher to discuss participation in more detail.
3) Clinicians will pass on the name and contact details of service users who meet the study's inclusion criteria and are willing to be contacted by a researcher. At this point, the researcher will ask the clinician whether there are any known risks that should be taken into account in arrangements to meet the potential participant. The clinician will also be asked to make a note of the potential participant's agreement to have their contact details passed on to the research team and to be contacted by a researcher. Names and contact details of potential participants and the clinician with whom they agreed to be contacted about the study will be kept in a password-protected document on a secure UCL database.
4) A study researcher will contact potential participants to explain the study, what taking part would involve and to answer any questions. For those potential participants who express an interest in taking part, the study researcher will send them a copy of the study participant information sheet.
5) If the participant is still interested, the researcher will arrange a convenient time and place for the potential participant to meet with them to complete the informed consent process and to take part in an individual usability testing session.
6) At this meeting, the researcher will invite any questions the service user might have about participating, assess the service user's capacity to provide informed consent, and seek written, informed consent before starting the usability testing session.

For peer review only - http://bmjopen.bmj.com/site/about/guidelines.xhtml

2.5.2 Lab-based usability testing session

The lab-based usability testing will comprise the first cycle of usability testing will be completed with 6 participants using a low-fidelity version of My Journey, accessed via the participant's own smartphone for the duration of the testing session only. The lab-based sessions will be facilitated by a study researcher, and will take place on NHS or UCL premises with access to wifi. User feedback from this cycle of testing will then be used to make changes to the design of the app before programming the native application. In the session, users will be asked to complete a brief demographic questionnaire, an audio-recorded "think aloud" task, and a semi-structured interview. The think-aloud approach, where the user gives a continuous commentary on their thoughts while using a system, is commonly used in usability testing in order to record users' immediate reactions to the system and enable an evaluation of how easily a system can be operated and to highlight any design issues. Semi-structured interviews will allow us to explore user experiences of My Journey 3.

Brief questionnaire

Following provision of written consent, participants will complete a short baseline demographic questionnaire. This questionnaire will ask participants about their use of digital technology, demographic data including age, gender, ethnicity and education level, and their use of mental health services.

Think aloud protocol

Participants will be asked to access the My Journey 3 app prototype on their own smartphone, and the research assistant will give an introduction to the app and its main functions. The research assistant will introduce the think aloud task using the instruction sheet. The information sheet and instructions will make it clear to the participant that the purpose of this task is to test the usability of the app, not the participant's abilities. Participants will be advised that they can input fictional information into the app if they wish, and that all information entered by the user into the app will be erased at the end of the session. When the participant is ready, the research assistant will start the audio-recording and ask the participant to try as best they can to complete the tasks while giving a think aloud commentary. The research assistant will make observational notes about the participant's interactions with the app. Prompts and support from the research assistant will be kept to a minimum during the task. The task should take no longer than 45 minutes.

Semi-structured interview

When the participant has finished the tasks, the research assistant will ask them to complete a short, audio-recorded, semi-structured interview about their experience of using the app. Questions will focus on the perceived ease of use and usefulness of the system, any concerns

For peer review only - http://bmjopen.bmj.com/site/about/guidelines.xhtml

they might have, to identify helpful, unhelpful and missing aspects of the app, and any suggestions for improvements. The interview should take no longer than 20 minutes.

The session will take no longer than 2 hours, and participants will be offered regular breaks. A full debrief will be given by the study researcher at the end of the session. Participants will be offered a £20 gift of cash to acknowledge their time and participation in the study.

2.6 Field study procedure

In this field study, 6 participants will test My Journey 3 smartphone app on their smartphones for a period of one month. Results of the field testing will be used to identify any necessary improvements or adjustments to the interview schedule, protocol for the intervention, or the app prior to the pilot RCT.

2.6.1 Field study Measures

The following measures will be included in the interview schedule for the pilot trial. In the field study, the interview schedule will be completed as a structured interview with a researcher at baseline and 4-week follow-up.

I. **Descriptive information** relating to social and demographic characteristics: age, gender, ethnicity, accommodation and living situation, employment status, educational attainment, internet and digital technology use, past service use, diagnosis, current prescribed psychiatric medication.
II. **Questionnaire on the Process of Recovery** (QPR; Neil et al., 2009) – a 22-item measure of self-rated recovery developed collaboratively by service user researchers and clinicians specifically for people with experience of psychosis.
III. **The Mental Health Confidence Scale** (MHCS; Carpinello et al., 2000) – is a 16-item service user-rated measure of self-efficacy beliefs of people diagnosed with mental health problems.
IV. **Warwick-Edinburgh Mental Well-Being Scale** (WEMWBS; NHS Health Scotland, University of Warwick & University of Edinburgh, 2007) – a 14-item scale comprised of only positively worded items relating to positive mental health that has demonstrated robust psychometric properties and is sensitive to change in mental health populations, including people with psychosis.
V. **The DIALOG scale** (DIALOG; Priebe et al., 2007) – an 11 item scale assessing satisfaction with 8 different life domains - physical health, mental health, job situation, accommodation, friendships, leisure activities, partner/family, and personal safety - and 3 treatment domains – medication, consultations with mental health professionals, and practical help received – rated by the service user on a Likert scale

For peer review only - http://bmjopen.bmj.com/site/about/guidelines.xhtml

ranging from 1 (=couldn't be worse) to 7 (=couldn't be better). It has demonstrated good psychometric properties in people with psychosis living in the community (Preibe et al., 2012).

VI. **The Positive and negative syndrome scale** (PANSS; Kay et al., 1987) – a widely used measure of positive and negative symptoms as well as general psychopathology.

The following information will also be collected at the specified time points:

i. **Usability, acceptability, and satisfaction**

Service users: At 4-week follow-up, participants will complete an audio-recorded semi-structured interview relating to the usability and acceptability of the app, and about their satisfaction with the support they received from the researcher and from their clinician. This will take no longer than 20 minutes.

Clinicians: After the main 4-week follow-up interview, a researcher will contact the EIS clinician who has been supporting the service user participant with the app intervention and ask them to complete a short semi-structured interview relating their experience of providing support with the app. This will take no longer than 20 minutes.

ii. **App usage**

Usage data will be collected for all participants in the intervention arm. The My Journey 3 app will automatically upload participant usage data (frequency, duration, and pattern of use) to a secure study server. The data collected will be a record of each time the user opens the app, whether this was in response to a prompt or not, and which components of the app they use. Data collected will not include any personal information (i.e. any text input or responses to self-rated questions).

2.6.2 Recruitment and consent

1) Researchers will seek help from clinicians in participating EISs to identify EIS service users who 1) meet the study's inclusion criteria and 2) are being seen by a clinician who is willing to attend a training session in using the app and to support the participant with using the app during their routine appointments. At this stage, clinicians will screen out service users who meet the study exclusion criteria, service users who are not interested in taking part in the study, and service users who do not have regular contact with a clinician who is willing to support them with using the app.
2) The recruitment procedure will then follow steps 2 – 4 from the lab-based usability testing (see section 2.5.1.2).
3) See 2.5.1.3
4) See 2.5.1.4

For peer review only - http://bmjopen.bmj.com/site/about/guidelines.xhtml

5) For potential participants who are still interested in taking part after reading the information sheet, the researcher will arrange a convenient time and place to meet with them to complete the informed consent process and baseline interview.
6) At this meeting, the researcher will invite any questions the service user might have about participating, assess the service user's capacity to provide informed consent, and seek written, informed consent before starting the structured baseline interview. In order to minimise loss to follow-up, and on receipt of written, informed consent, the participant will be asked 1) their preferred contact details and 2) their permission for researchers to contact a nominated close other and staff members whom staff could contact if unable to contact the participant directly, for further contact regarding their participation in the study.

2.6.3 Baseline assessment

Following provision of written consent, the researcher will complete the baseline interview with the participant. This will take no longer than one hour. Participants will be offered a £20 gift in cash in recognition of their time and participation.

2.6.4 Intervention Procedure

After completion of the baseline assessment, a researcher will arrange to meet with the participant and an appropriate EIS clinician at a suitable time and place, according to any risk limitations. The researcher will ensure that wifi access is available in the building in which the meeting takes place. At this session, the participant will be asked to download the My Journey 3 app onto their smartphone. The researcher will then give a demonstration of the app and its main functions to the participant and their clinician. Participants will be given the opportunity to practice using the app, with the support of their clinician if appropriate, and to ask any questions. The participant will then be invited to input at least one personal goal and the steps and support needed to achieve it, one thing to do to keep well, one early warning sign and one trigger and corresponding actions to take, and to input an emergency plan. Participants will also be encouraged to input key support contacts and any medication they are currently taking. The researcher, participant and their clinician will review the participant's most typical daily routine and discuss what time of day it would be most helpful to receive reminders to engage with the app. Participants will then be given the opportunity to ask any questions they might have. Support from the researcher and clinician will be provided as appropriate. This meeting will take no longer than 2 hours.

In the initial meeting, the researcher will encourage the participant to keep their smartphone with them as they go about their everyday lives for the following four weeks. They will be encouraged to discuss their recovery goals and relapse prevention plan in their next

For peer review only - http://bmjopen.bmj.com/site/about/guidelines.xhtml

appointment with their clinician. The researcher will arrange to send a reminder to this effect to the clinician and the service user prior to their next appointment, with their agreement. Participants will be asked to contact the research team to report any technical issues: contact details will be included within the app and provided at the initial meeting with the researcher. The participant will be given contact details for the study researcher, and informed that they will be available for support with technical issues during office hours. In addition, a researcher will contact the participant 7 days after the initial training session to ask them if they have had any technical problems or difficulties with the app.

2.6.5 Four week follow-up assessment

Four weeks after the first assessment, and as soon as possible after this, a researcher will contact the participant to arrange a follow-up meeting. At the follow-up meeting, the researcher will seek informed consent from the participant and then complete the follow-up assessment interview with participant. Participants will be offered a further £20 gift of cash to acknowledge their time and participation in the study.

2.6.6 Clinician interview

Four weeks after the first assessment with a participant, a researcher will contact the clinician who has been supporting the participant with the intervention to ask if they are willing to complete a short interview with a researcher. They will be provided a copy of the clinician study information sheet, and if they still interested, a researcher will seek their written or audio-recorded verbal consent to take part in an interview.

On receipt of informed consent, a researcher will contact the clinician and ask them to complete an audio-recorded semi-structured interview in which they will be asked questions about their experience of supporting the participant with the app intervention, and about helpful, unhelpful, and missing aspects of the app and the training session with the research assistant. This interview will take no longer than 20 minutes.

2.7 Analysis

In line with the aims of the usability study, data analysis will involve:

1) Analysis of baseline demographic data using descriptive statistics;

2) Thematic analysis of qualitative data from the think-aloud testing and semi-structured interviews.

For peer review only - http://bmjopen.bmj.com/site/about/guidelines.xhtml

For the field study, analysis will additionally involve:

3) Analysis of usage data using descriptive statistics

3. Phase II: Pilot RCT

3.1 Setting

Participants will be recruited from five Early Intervention Service teams across Camden and Islington NHS Foundation Trust, East London NHS Foundation Trust, and Surrey and Borders Partnership NHS Foundation Trust.

3.2 Sample size

Julious (2005) recommends that pilot RCTs include 12 completer participants per trial arm. We have conservatively assumed a 40% attrition rate from the proposed study and therefore aim to recruit 20 participants in total (20 per trial arm).

3.3 Participants

Participants will be 40 people, aged 16 or older, with first-episode psychosis who are currently engaged with one of the following Early Intervention Services: Camden and Islington NHS Foundation Trust Early Intervention Service, East London Early Intervention Services (Newham Early Intervention Service, Tower Hamlets Early intervention Service, City & Hackney Early Intervention Service), and Surrey and Borders NHS Foundation Trust Early Intervention in Psychosis Service.

Inclusion criteria

Participants will be eligible for the study if they:

1) Are aged 16 or older
2) Have a diagnosis of psychosis
3) Own an Android smartphone
4) Are being seen by clinicians in an EIS
5) Speak and understand English

Exclusion criteria

Participants will be excluded from the study on the basis that they:

1) Are aged 15 or younger
2) Do not own an Android smartphone
3) Are not currently being seen by clinicians in an EIS

For peer review only - http://bmjopen.bmj.com/site/about/guidelines.xhtml

4) In the view of their EIS team, pose such a high risk to others that it would be unsafe for a researcher to meet with them, even in a mental health service setting.
5) Lack capacity to provide informed consent to take part in the study

3.4 Measures

The following measures will be included in a structured interview that will be conducted by a researcher with all participants at baseline, post-intervention (four-month follow-up; four months post baseline), and twelve-month follow-up (twelve months post baseline).

I. **Descriptive information** relating to social and demographic characteristics: age, gender, ethnicity, accommodation and living situation, employment status, use of the internet and digital technology, educational attainment, service use, diagnosis, and current prescribed psychiatric medication.
II. **Questionnaire on the Process of Recovery** (QPR; Neil et al., 2009)
III. **The Mental Health Confidence Scale** (MHCS; Carpinello et al., 2000)
IV. **Warwick-Edinburgh Mental Well-Being Scale** (WEMWBS; NHS Health Scotland, University of Warwick & University of Edinburgh, 2007)
V. **The DIALOG scale** (DIALOG; Priebe et al., 2007)
VI. **The Positive and negative syndrome scale** (PANSS; Kay et al., 1987)

The following information will also be collected at the specified time points:

i. Usability, acceptability, and satisfaction

Service users: **After the main four-month follow-up interview, a researcher will contact service user** participants in the intervention arm and ask them to complete an audio-recorded semi-structured interview relating to the usability and acceptability of the app, and about the support they received from the researcher and from their clinician. This will take no longer than 20 minutes.

Clinicians: **After the main four-month follow-up interview, a researcher will contact the clinicians** who have been supporting service user participants in the intervention arm and ask them to complete a short semi-structured interview relating to their experience of providing support with the app. They will also be asked to provide basic demographic data (including ethnicity, job title, and age group). This will take no longer than 20 minutes.

ii. App usage

Usage data will be collected for all participants in the intervention arm for the duration of their participation in the trial **(baseline to 12 month follow-up).** The My Journey 3 app will automatically upload participant usage data (frequency, duration, and pattern of use) to a secure study server. The data collected will be a record of each time the user opens the app, whether this was in response to a prompt or not, and which

For peer review only - http://bmjopen.bmj.com/site/about/guidelines.xhtml

components of the app they use. Data collected will not include any personal information (i.e. any text input or responses to self-rated questions).

iii. Service engagement

At baseline and at **12-month** follow-up, a study researcher will contact the participant's EIS care coordinator and ask them to complete **The Service Engagement Scale (SES; Tait et al., 2002)** – a 14 item questionnaire completed by the service user's clinician that measures engagement on four dimensions: availability for appointments, collaboration, help-seeking, and treatment adherence.

In addition, the following information will be collected from patient records at baseline and at one year after entry into the study:

a. Current diagnosis
b. Current care cluster
c. Use of mental health services during the previous year (number of admissions to acute care, inpatient bed days, use of community mental health services (i.e. services used and number of kept and missed appointments))
d. Care plan approach status

3.5 Procedures

3.5.1 Recruitment and consent

Researchers will provide all sites with posters and leaflets to advertise the study and encourage interested service users to register their interest with a member of the EIS team. Researchers will also provide all clinicians in the participating EIS service with the clinician information sheet. Researchers will seek help from clinicians in participating EISs to identify EIS service users who 1) meet the study's inclusion criteria and 2) are being seen by a clinician who is willing to attend a training session in using the app and to support the participant with using the app during their routine appointments. Clinicians who are known to the potential participant will make initial contact with them to give a brief explanation of the study and to ask if they are willing to be contacted by a researcher to discuss participation in more detail. At this stage, clinicians will screen out service users who meet the study exclusion criteria or who are not interested in taking part in the study. Clinicians will pass on the name and contact details of service users who meet the study's inclusion criteria and are willing to be contacted by a researcher. At this point, the researcher will ask the clinician whether there are any known risks that should be taken into account in arrangements to meet the potential participant. The clinician will also be asked to make a note of the potential participant's agreement to have their contact details passed on to the research team and to be contacted by a researcher. Names and contact details of potential participants and the clinician with

For peer review only - http://bmjopen.bmj.com/site/about/guidelines.xhtml

whom they agreed to be contacted about the study will be kept in a password-protected document on a secure UCL database.

Clinicians will also be asked to keep a record of the number of people on their caseload who have been 1) screened out prior to introducing the study, and a brief, anonymised reason why they were considered unsuitable, 2) approached about the study; and 3) declined to be contacted by a researcher and a brief, anonymised reason why they declined.

If the participant is still interested, the researcher will arrange with them a time and place to meet, at the convenience of the service user and in line with any risk limitations. At this initial meeting, researchers will invite any questions the service user might have about participating, assess the service user's capacity to provide informed consent, and then seek written, informed consent. In order to minimise loss to follow-up, and on receipt of written, informed consent, the participant will be asked 1) their preferred contact details and 2) their permission for researchers to contact a nominated close other and staff members whom staff could contact if unable to contact the participant directly, for further contact regarding their participation in the study.

Following randomisation, the participant's GP and the EIS from which they were recruited will be informed in writing of their consent to participate in the study, provided with a copy of their signed consent form, and informed which group the participant has been allocated to.

3.5.2 Randomisation

Following baseline assessment, participants will be block randomised to intervention and control groups. Randomisation will be conducted by a researcher who is independent to the research team.

A researcher will contact participants to tell them which group they have been allocated to, and for those in the intervention group, to make arrangements for their intervention training session with a researcher.

It will not be possible to blind participants or researchers to their group allocation. Baseline interviews will be conducted prior to randomisation and will therefore be blind to allocation. A researcher will conduct randomisation, provide support with the intervention, inform GPs and EIS teams about participants' allocation, and conduct sections of the post-intervention interview that ask about the participant's experience of the intervention. The same researcher. will also conduct the main post-intervention and follow-up interviews.

For peer review only - http://bmjopen.bmj.com/site/about/guidelines.xhtml

3.5.3 Assessment

Baseline interview

After participants have provided written informed consent to participate in the study but before they have been randomised, a study researcher will complete the study baseline measures with all participants. The interview will last for approximately an hour, and will take place at the participant's home or on NHS or University premises, according to participant preference and within limits advised by the participant's EIS team. Participants will be offered regular breaks or to complete the interview over two or more meetings if necessary. Participants will be offered a £20 gift of cash to acknowledge their time and participation in the study.

Post-intervention interview

Four months after completion of baseline measures, or as soon as possible after this, a study researcher will contact the participant to invite them to attend a post-intervention interview, explain what the interview would involve, answer any questions, ask whether the participant is willing to attend, and arrange a time and place to meet. At this meeting, the researcher will again seek written, informed consent from the participant prior to commencing the interview. The interview will last around an hour, and participants will be offered a £20 gift of cash to acknowledge their time and participation. The study researcher will be blind to group allocation, and will ask the participant prior to the meeting not to reveal whether they received the intervention or not.

After the main four-month follow-up interview, a researcher will contact participants allocated to the treatment arm to complete a short semi-structured interview about their experience of the intervention, either in person or as a telephone interview.

Twelve month Follow-up interview

At least 12 months after completion of baseline measures, or as soon possible after this, a researcher will contact the participant to invite them to attend a follow-up interview, following the same protocol as for the post-intervention interview. Participants will be offered a £20 gift of cash to acknowledge their time and participation.

Researchers conducting research assessment interviews will seek to minimise missing data through manually checking data collection booklets during the assessment and prompting participants to complete all questions. Missing data will be clearly coded in the study database.

For peer review only - http://bmjopen.bmj.com/site/about/guidelines.xhtml

3.5.4 Control procedure

Participants in both arms of the trial will receive treatment as usual. This will consist of care from an Early Intervention Service in line with national and local service guidelines.

3.5.5 Intervention procedure

The intervention procedure as detailed for the field trial (see section 2.6.4) will be followed for all participants allocated to the intervention arm, but participants will be asked to engage with the app as they go about their everyday lives for 6 weeks after downloading the My Journey 3 app.

3.5.6 Clinician interview (intervention arm only)

Four months after the service user participant's completion of baseline measures, a researcher will contact the clinician who has been supporting the participant with the intervention to ask if they are willing to complete a short interview with a researcher. They will be provided with a copy of the clinician study information sheet, and if they still interested, a researcher will seek their written or audio-recorded verbal consent to take part in the interview.

On receipt of informed consent, a researcher will contact the clinician and ask them to complete an audio-recorded semi-structured interview in which they will be asked questions about their experience of supporting the participant with the app intervention, facilitators and barriers to providing support, and about helpful, unhelpful, and missing aspects of the app and the training session with the research assistant. This interview will take no longer than 20 minutes.

3.5.7 Service engagement data

Baseline data collection

As soon as possible after the baseline interview, a researcher will contact the service user's EIS care coordinator and ask them to complete the service engagement questionnaire in relation to the participant.

Twelve month data collection

At least twelve months after completion of baseline measures and as soon as possible after this, a researcher will contact the participant's EIS care coordinator and ask them to complete the service engagement questionnaire in relation to the participant.

For peer review only

For peer review only - http://bmjopen.bmj.com/site/about/guidelines.xhtml

3.5.8 Data from patient records

After the recruitment target has been met, a study researcher will contact an appropriate informatics or administration team in the participating trusts, and provide a standardised protocol of the information required for the study. The study researchers will provide a list of consenting participants' Trust identification number (e.g. RiO number) and study ID number. The appropriate Trust personnel will be asked to provide the data to the research team, with study ID numbers as the only identifying information to avoid any risks to data protection when transferring the information.

One year after all participants have been recruited into the study, a study researcher will again provide the appropriate department in the trust with a data collection schedule and follow the same procedure as above. Study researchers will attempt to obtain any data that is not available from Trust records from other NHS or voluntary services or from the participant, in accordance with the participants' written consent.

3.6 Analysis

A CONSORT diagram will be produced to report on the recruitment, retention, and progress of participants through the trial. Baseline demographic characteristics will be analysed using descriptive statistics. Baseline between-group differences on demographic variables and all outcome variables will be examined as a randomisation check. The pattern of missing data will be examined to check whether data is missing at random.

In line with the aims of the pilot study data analysis will involve:

1) Calculating the rate of recruitment as the number of participants referred per recruitment month.

2) Calculating the percentage of participants who drop out of the study.

3) Analysis of usability and usage data using descriptive statistics

4) Calculating the group x time effect size (using mixed ANOVA) and 95% confidence interval between the intervention and waitlist control on the QPR, MHCS, WEMWBS, DIALOG, PANSS, and SES. There will be one within-group independent variable (time: baseline, post-intervention and 12-month follow-up) and one between-group variable (group: intervention and wait-list control). Both per-protocol and intention-to-treat effect sizes (last observation carried forward) will be reported.

For peer review only - http://bmjopen.bmj.com/site/about/guidelines.xhtml

5) Thematic analysis of qualitative data from interviews with service user and staff participants. Analyses will be conducted collaboratively by a group of researchers within the team, to enhance the validity of the analysis.

4. Data storage

Data collection forms will not contain participant's names but will use a unique study ID that could not be linked to the participant by anyone outside the research team. The questionnaire responses will contain non-identifiable information including ethnicity, service use, and gender. A single password protected file stored on a secure University College London server will match study IDs with participant names and contact details. Paper copies of questionnaires will be kept in locked filing cabinets at UCL. Paper consent forms and any identifying information will be stored securely at University College London, separately from data collection forms.

Audio recordings (of usability testing sessions or qualitative interviews) will be transferred from audio-recorders into a folder only accessible to the research team on a secure server at University College London. Audio-recordings will be removed immediately from the audio-recorder.

A database of all quantitative study data will be stored securely on a secure University College London server accessible only to members of the research team using secure log-ins. Only study IDs will be used on this database.

Data will be stored securely at University College London for one year after the end of the study, before being archived securely in accordance with University College London data protection procedures.

Usage data from the My Journey app will be encrypted and uploaded directly onto a secure server when the user has internet access. This usage data will not contain personal information that the user has entered within the app.

5. App safety, security, and maintenance

A clear statement that the My Journey app is not intended to be a substitute for professional advice, diagnosis or treatment will be included within the app and within the user guide provided to all participants before the app is downloaded.

The user guide and the initial within-app setup instructions will advise participants to set a secure log-on to password protect their phone in order to protect participant confidentiality

For peer review only - http://bmjopen.bmj.com/site/about/guidelines.xhtml

and privacy should their phone be lost, stolen, or handled by others. This advice will also be given in the initial training session with a researcher.

All personal data (i.e. responses to self-report questions, text user input) will be stored within the app. There is a sharing functionality within the MyJourney App should users wish to share some or all of their data with a trusted mental health worker, family member, friend or other trusted third party. This uses the built-in sharing functionality of their smart device (such as e-mail) and the participant information sheet and in-app disclaimer makes it clear that it is the users' responsibility to ensure this meets their own data security requirements. Both the app and the study participant information advises participants that all content and information available or shared through the My Journey app is for reference and information purposes only, and is not designed as a substitute for seeking professional medical advice, diagnosis, or treatment.

We will endeavour to identify and resolve any unintended issues and assess whether they pose any safety or security risks to users during the usability testing stage, before the app is taken to pilot trial. In the field and pilot trials, a researcher will contact the participant 7 days after they have downloaded the app to check whether they have experienced any difficulties with it. In addition, a mechanism for providing feedback on the app and for users to report any safety or security issues or bugs will be provided within the app. This will be fed back to the developer, who will check for any reported issues regularly. All issue reports will be tracked and shared with the research team. A trial safety protocol specifying serious adverse events (SAEs) and reporting and reviewing procedures in the event of SAEs will be developed, and EIS care coordinators will be alerted to contact the study team in the event of SAEs involving trial participants during the active treatment phase of the trial. Applicant DO brings relevant expertise as a member of Priment Clinical Trials Unit and will advise on safety procedures.

6. Research governance and oversight

Ethical approval for the study and approvals from R&D departments and Early Intervention Services in participating trust will be sought before recruitment to the study begins. The study will be registered with ISRCTN before the study begins.

A study steering group, independent to the study research team, will be established. Members will include researchers with expertise in developing and testing e-health and m-health interventions, clinicians with experience of Early Intervention Services, people with lived experience of first episode psychosis and Early Intervention Services and a carer. The steering group will meet before approvals for the study are sought and then at six monthly intervals to oversee the conduct of the study.

For peer review only - http://bmjopen.bmj.com/site/about/guidelines.xhtml

If any amendments to the study protocol are required, approval for these will be sought from the Research Ethics Committee and participating Trusts.

7. Dissemination

We will report the results of the study in a peer-reviewed report to the study funders, the NIHR CLAHRC North Thames. We will seek to also report findings in peer-reviewed scientific journals and conferences, magazines or web publications which reach an audience of health professionals, service users and carers.

All participants will be asked at the point of entry into the study whether they would like to be informed of the outcomes of the study, and whether they would like to receive these results via email or the post. Results will be written up in plain English as a report to send to those participants who have indicated that they wish to receive them. We will consult with service user and carer members of the steering group to ensure the report is clear and easy to understand.

8. Timeline

Please see figure 1.

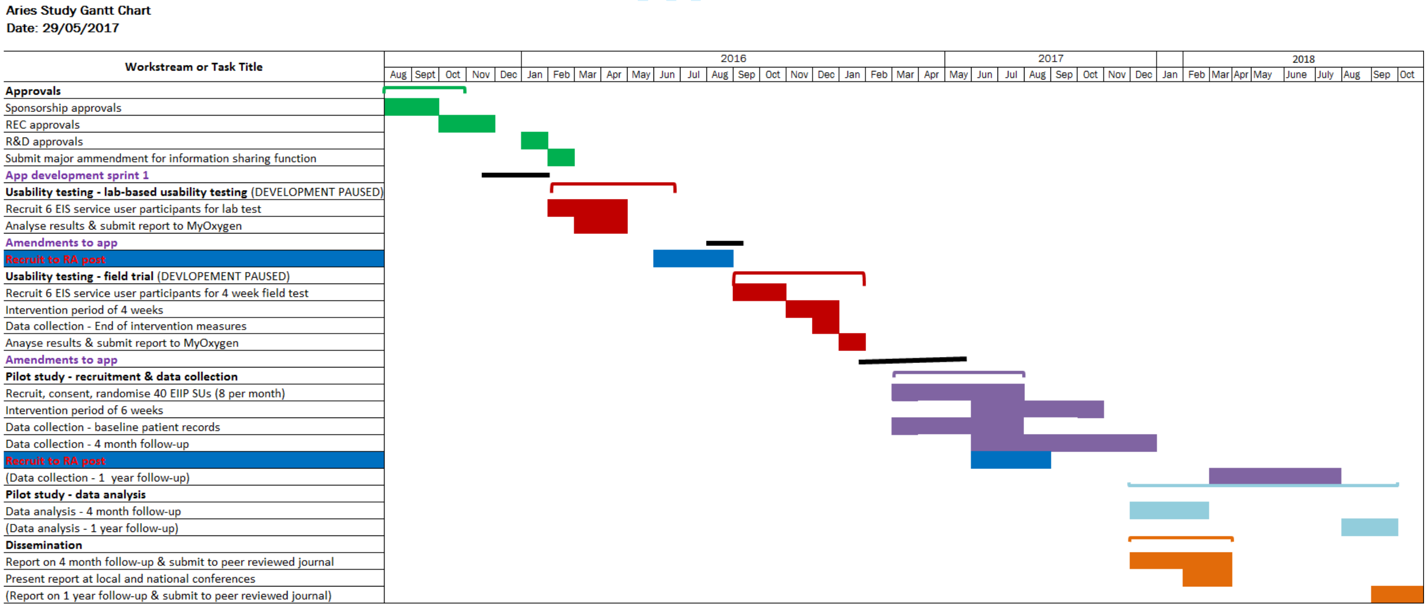

Figure 1: ARIES study GANNT chart

9. References

Addington, D., Addington, M. D. & Patten, S. (2007). Relapse rates in an early psychosis treatment service. *Acta Psychiatrica Scandinavia 115*(2), 126-131.

For peer review only - http://bmjopen.bmj.com/site/about/guidelines.xhtml

Alvarez-Jimenez, M., Gleeson, J. F., Cotton, S., Wade, D., Gee, D., Pearce, T., …McGorry, P.D. (2009). Predictors of adherence to cognitive-behavioural therapy in first-episode psychosis. *Canadian Journal of Psychiatry 54*(10), 710-718.

Alvarez-Jimenez, M., Parker A. G., Hetrick, S.E., McGorry, P.D., & Gleeson, J. F. (2011). Preventing the second episode: a systematic review and meta-analysis of psychosocial and pharmacological trials in first-episode psychosis. *Schizophrenia Bulletin 37*(3), 619-630.

Alvarez-Jimenez, M., Priede, A., Hetrick, S.E., Bendall, S., Killackey, E., Parker, A.G., …Gleeson, J.F., (2012a). Risk factors for relapse following treatment for first episode psychosis: a systematic review and meta-analysis of longitudinal studies. *Schizophrenia Research*, 139(1-3), 116-128.

Alvarez-Jimenez, M., Gleeson, J.F., Bendall, S., Lederman, R., Wadley, G., Killackey, E., McGorry, P.D., (2012b). Internet-based interventions for psychosis: a sneak-peek into the future. *Psychiatric Clinics of North America, 35*(3), 735–747.

Alvarez-Jimenez, M., Alcazar-Corcoles, M. A., Gonzalez-Blanch, C., Bendall, S., McGorry, P.D., Gleeson, J. F. (2014). Online, social media and mobile technologies for psychosis treatment: a systematic review on novel user-led interventions. *Schizophrenia Research 156*(1), 96-106.

Arain, M., Campbell M. J., Cooper, C.L., & Lancaster, G.A (2010). What is a pilot or feasibility study? A review of current practice and editorial policy. *BMC Medical Research Methodology 10*(67), 1471-2288.

Ben-Zeev, D., Brenner, C.J., Begale, M., Duffecy, J., Mohr, D.C., & Mueser, K.T. (2014). Feasibility, Acceptability, and preliminary efficacy of a smartphone intervention for schizophrenia. *Schizophrenia Bulletin, 20(6),* 1244-1253.

Bird, V., Premkumar, P., Kendall, T., Whittington, C., Mitchell, J., & Kuipers, E. (2010). Early intervention services, cognitive-behavioural therapy and family intervention in early psychosis: systematic review. *British Journal of Psychiatry, 197*(5): 350-356.

Birnbaum, M. L., Rizvi, A. F., Correll, C.U., & Kane, J.M. (2015). Role of social media and the Internet in pathways to care for adolescents and young adults with psychotic disorders and non-psychotic mood disorders. *Early Intervention Psychiatry 23*(10), 12237.

Carpinello, S.E., Knight, E.L., Markowitz, F.E., & Pease, E.A. (2000). The development of the mental health confidence scale: A measure of self-efficacy in individuals diagnoses with mental disorders. *Psychiatric Rehabilitation Journal, 23,* 236-243.

Cook, J.A., Copeland, M.E., Hamilton, M.M., Jonikas, J.A., Razzaro, L.A., Floyd, C.B., …Grey, D.D. (2009). Initial outcomes of a mental illness self-management program based on wellness recovery action planning. *Psychiatric Services,* 60(2), 246 - 249.

Depp, C. A., Mausbach B., Granhom, E., Cardenas, V., Ben-Zeev, D., Patterson, T.L., …Jeste, D.V. (2010). Mobile interventions for severe mental illness: design and preliminary data from three approaches. *The Journal of Nervous and Mental Disease 198*(10), 715-721.

For peer review only - http://bmjopen.bmj.com/site/about/guidelines.xhtml

Gafoor, R., Nitsch, D., McCrone, P., Craig, T. K., Garety, P.A., Power, P., & McGuire, P. (2010). Effect of early intervention on 5-year outcome in non-affective psychosis. *British Journal of Psychiatry, 196*(5), 372-376.

Highton-Williamson, E., Priebe S., & Giacco, D. (2015). Online social networking in people with psychosis: A systematic review. *International Journal of Social Psychiatry 61*(1), 92-101.

Julious, S.A. (2005). Sample size of 12 per group rule of thumb for a pilot study. *Pharmaceutical Statistics, 4,* 287-291.

Kasckow, J., Felmet, K., Appelt, C., Thompson, R., Rotondi, A. & Haas, G. (2014). Telepsychiatry in the assessment and treatment of schizophrenia. *Clinical Schizophrenia and Related Psychoses 8*(1), 021513.

Kay, S.R., Fiszbein, A., & Opier, L.A. (1987). The Positive and NEgative Syndrome Scale (PANSS) for schizophrenia. *Schizophrenia Bulletin, 12,* 261-276.

Lederman, R., Wadley, G., Gleeson, J., Alvarez-Jimenez, M., & Spiteri-Stained, A. (2011). Supporting young people with psychosis in the community: An ICT enabled relapse prevention tool. PACIS 2011 Procedings. Paper 104. Retrievable from: http://aisel.aisnet.org/pacis2011/104.

Leon, A.C., Davis, L.L., & Karemer, H.C. (2011). The role and interpretation of pilot studies in clinical research. *Journal of Psychiatric Research, 45*(5), 626-629.

Liberman, R.P. (1986). Coping and competence as protective factors in the vulnerability-stress model of schizophrenia. In M.J. Goldstein, I. Hand., & K. Hahlweg (eds.), *Treatment of Schizophrenia*. Berlin: Springer Berlin Heidelberg.

Mueser, K. T., Meyer P. S., Penn, D.L., Clancy, R., Clancy, D.M., Salyers, M.P. (2006). The Illness Management and Recovery program: rationale, development, and preliminary findings. *Schizophrenia Bulletin 32*(1), 9.

Mueser, K. T., Deavers F., Penn, D.L., & Cassisi, J.E. (2013). Psychosocial treatments for schizophrenia. *Annual Review of Clinical Psychology 9*, 465-497.

Neil, S., Kilbride, M., Pitt L., Nothard, S,. Welford, M., Sellwood, W., & Morrison, A. (2009). The questionnaire about the process of recovery (QPR): A measurement tool developed in collaboration with service users. *Psychosis, 1,* 145-155.

Norman, R. M., Manchanda R., MAlla, A.K., Windell, D., Harricharan, R., & Northcott, S. (2011). Symptom and functional outcomes for a 5 year early intervention program for psychoses. *Schizophrenia Research 129*(2-3), 111-115.

OfCom. (2013). The Communications Market Report. London: Ofcom

OfCom, (2014). The Communications Market Report. London: Ofcom

Plaistow J, Birchwood M. (1996). Back in the Saddle: A Guide to Relapse Prevention (unpublished manual). 1996

For peer review only - http://bmjopen.bmj.com/site/about/guidelines.xhtml

Priebe S, McCabe R, Bullenkamp J, Hansson L, Lauber C, Martinez-Leal R, et al. (2007). Structured patient-clinician communication and 1-year outcome in community mental healthcare—cluster randomised controlled trial. *British Journal of Psychiatry,19, 420-426.*

Priebe S, Golden E, McCabe R, Reininghaus U. (2012). Patient-reported outcome data generated in a clinical intervention in community mental health care—psychometric properties. *BMC Psychiatry (12)*, 113

Rotundi, A.J., Sinkule, J., Haas, G.L., Spring, M.B., Litschge, C.M., Newhill, C.E., …Anderson, C.M. (2007). Designing websites for persons with cognitive deficits: design and usability of a psychoeducational intervention for persons with severe mental illness. *Psychological Services, 4*(3), 202-224.

Schrank, B., Sibitz I., Unger, A., & Amering, M. (2010). How patients with schizophrenia use the internet: qualitative study. *Journal of Medical Internet Research, 12*(5).

Seko, Y., Kidd, S. Wiljer, D., & McKenzie, K. (2014). Youth mental health interventions via mobile phones: a scoping review. *Cyberpsychology, Behavavior, and Social Networking 17*(9), 591-602.

Tait, L., Birchwood, M., & Trower, P. (2002). A new scale (SES) to measure engagement with community mental health services. *Journal of Mental Health, 11*(2), 191-198.

Tennant, R., Hiller, L., Fishwick, R., Platt, S., Joseph, S., Weich, S., …Stewart-Brown, S. (2007). The Warwick-Edinburgh Mental Well-Being Scale (WEMWBS): Development and UK validation. *Health and Quality of Life Outcomes, 5*:63.

Valimaki, M., Anttila, M. et al. (2008). Design and development process of patient-centered computer-based support system for patients with schizophrenia spectrum psychosis. *Information, Health and Social Care 33*(2), 113-123.

Valimaki, M., Hatonen, H. Lahti, M., Kuosamen, L., & Adams, C.E. (2012). Information and communication technology in patient education and support for people with schizophrenia. *Cochrane Database of Systematic Reviews 17*(10).

van der Krieke, L., Wunderink L., Emerencia, A.C., de Jonge, P., & Sytema, S. (2014). E-mental health self-management for psychotic disorders: state of the art and future perspectives. *Psychiatric Services 65*(1), 33-49.

For peer review only - http://bmjopen.bmj.com/site/about/guidelines.xhtml

CONSORT 2010 checklist of information to include when reporting a pilot or feasibility trial*

Section/Topic	Item No	Checklist item	Reported on page No
**Title and abstract**			
	1a	Identification as a pilot or feasibility randomised trial in the title	Title Page
	1b	Structured summary of pilot trial design, methods, results, and conclusions (for specific guidance see CONSORT abstract extension for pilot trials)	Abstract
**Introduction**			
Background and objectives	2a	Scientific background and explanation of rationale for future definitive trial, and reasons for randomised pilot trial	Introduction
	2b	Specific objectives or research questions for pilot trial	Introduction
**Methods**			
Trial design	3a	Description of pilot trial design (such as parallel, factorial) including allocation ratio	Methods (design)
	3b	Important changes to methods after pilot trial commencement (such as eligibility criteria), with reasons	N/A
Participants	4a	Eligibility criteria for participants	Methods (participants)
	4b	Settings and locations where the data were collected	Methods (setting)
	4c	How participants were identified and consented	Methods (recruitment strategy)
Interventions	5	The interventions for each group with sufficient details to allow replication, including how and when they were actually administered	Methods (interventions)
Outcomes	6a	Completely defined prespecified assessments or measurements to address each pilot trial objective specified in 2b, including how and when they were assessed	Methods (outcomes)
	6b	Any changes to pilot trial assessments or measurements after the pilot trial commenced, with reasons	N/A
	6c	If applicable, prespecified criteria used to judge whether, or how, to proceed with future definitive trial	N/A
Sample size	7a	Rationale for numbers in the pilot trial	Methods (participants)

For peer review only

	7b	When applicable, explanation of any interim analyses and stopping guidelines	Methods (analysis)
Randomisation:			
Sequence generation	8a	Method used to generate the random allocation sequence	Methods (randomisation)
	8b	Type of randomisation(s); details of any restriction (such as blocking and block size)	Methods (randomisation)
Allocation concealment mechanism	9	Mechanism used to implement the random allocation sequence (such as sequentially numbered containers), describing any steps taken to conceal the sequence until interventions were assigned	Methods (randomisation)
Implementation	10	Who generated the random allocation sequence, who enrolled participants, and who assigned participants to interventions	Methods (randomisation)
Blinding	11a	If done, who was blinded after assignment to interventions (for example, participants, care providers, those assessing outcomes) and how	Methods (randomisation)
	11b	If relevant, description of the similarity of interventions	N/A
Statistical methods	12	Methods used to address each pilot trial objective whether qualitative or quantitative	Methods (analysis)
**Results**			
Participant flow (a diagram is strongly recommended)	13a	For each group, the numbers of participants who were approached and/or assessed for eligibility, randomly assigned, received intended treatment, and were assessed for each objective	Results (feasibility of trial design)
	13b	For each group, losses and exclusions after randomisation, together with reasons	Results (feasibility of trial design)
Recruitment	14a	Dates defining the periods of recruitment and follow-up	Results (feasibility of trial design)
	14b	Why the pilot trial ended or was stopped	N/A
Baseline data	15	A table showing baseline demographic and clinical characteristics for each group	Results (sample characteristics)
Numbers analysed	16	For each objective, number of participants (denominator) included in each analysis. If relevant, these numbers should be by randomised group	Results (participant outcomes)

For peer review only

Outcomes and estimation	17	For each objective, results including expressions of uncertainty (such as 95% confidence interval) for any estimates. If relevant, these results should be by randomised group	Results (participant outcomes)
Ancillary analyses	18	Results of any other analyses performed that could be used to inform the future definitive trial	N/A
Harms	19	All important harms or unintended effects in each group (for specific guidance see CONSORT for harms)	Results (participant outcomes)
	19a	If relevant, other important unintended consequences	N/A
**Discussion**			
Limitations	20	Pilot trial limitations, addressing sources of potential bias and remaining uncertainty about feasibility	Discussion (strength and limitations)
Generalisability	21	Generalisability (applicability) of pilot trial methods and findings to future definitive trial and other studies	Discussion (strength and limitations)
Interpretation	22	Interpretation consistent with pilot trial objectives and findings, balancing potential benefits and harms, and considering other relevant evidence	Discussion (conclusions)
	22a	Implications for progression from pilot to future definitive trial, including any proposed amendments	Discussion (conclusions)
**Other information**			
Registration	23	Registration number for pilot trial and name of trial registry	Abstract
Protocol	24	Where the pilot trial protocol can be accessed, if available	Additional file 3
Funding	25	Sources of funding and other support (such as supply of drugs), role of funders	Funding
	26	Ethical approval or approval by research review committee, confirmed with reference number	Methods (design)

Citation: Eldridge SM, Chan CL, Campbell MJ, Bond CM, Hopewell S, Thabane L, et al. CONSORT 2010 statement: extension to randomised pilot and feasibility trials. BMJ. 2016;355.
*We strongly recommend reading this statement in conjunction with the CONSORT 2010, extension to randomised pilot and feasibility trials, Explanation and Elaboration for important clarifications on all the items. If relevant, we also recommend reading CONSORT extensions for cluster randomised trials, non-inferiority and equivalence trials, non-pharmacological treatments, herbal interventions, and pragmatic trials. Additional extensions are forthcoming: for those and for up to date references relevant to this checklist, see www.consort-statement.org.

For peer review only
